# Predicting blood–brain barrier permeability of molecules with a large language model and machine learning

**DOI:** 10.1038/s41598-024-66897-y

**Published:** 2024-07-09

**Authors:** Eddie T. C. Huang, Jai-Sing Yang, Ken Y. K. Liao, Warren C. W. Tseng, C. K. Lee, Michelle Gill, Colin Compas, Simon See, Fuu-Jen Tsai

**Affiliations:** 1 NVIDIA AI Technology Center, NVIDIA Corporation, Santa Clara, USA; 2 Department of Medical Research, China Medical University Hospital, China Medical University, Taichung, Taiwan; 3 School of Chinese Medicine, College of Chinese Medicine, China Medical University, China Medical University Children’s Hospital, No. 2, Yude Road, Taichung, 404332 Taiwan; 4 China Medical University Children’s Hospital, Taichung, Taiwan

**Keywords:** Blood–brain barrier (BBB) permeability, Machine learning, Artificial intelligence (AI), Natural Products Research Laboratories (NPRL), Computational biology and bioinformatics, Mathematics and computing

## Abstract

Predicting the blood–brain barrier (BBB) permeability of small-molecule compounds using a novel artificial intelligence platform is necessary for drug discovery. Machine learning and a large language model on artificial intelligence (AI) tools improve the accuracy and shorten the time for new drug development. The primary goal of this research is to develop artificial intelligence (AI) computing models and novel deep learning architectures capable of predicting whether molecules can permeate the human blood–brain barrier (BBB). The in silico (computational) and in vitro (experimental) results were validated by the Natural Products Research Laboratories (NPRL) at China Medical University Hospital (CMUH). The transformer-based MegaMolBART was used as the simplified molecular input line entry system (SMILES) encoder with an XGBoost classifier as an in silico method to check if a molecule could cross through the BBB. We used Morgan or Circular fingerprints to apply the Morgan algorithm to a set of atomic invariants as a baseline encoder also with an XGBoost classifier to compare the results. BBB permeability was assessed in vitro using three-dimensional (3D) human BBB spheroids (human brain microvascular endothelial cells, brain vascular pericytes, and astrocytes). Using multiple BBB databases, the results of the final in silico transformer and XGBoost model achieved an area under the receiver operating characteristic curve of 0.88 on the held-out test dataset. Temozolomide (TMZ) and 21 randomly selected BBB permeable compounds (Pred scores = 1, indicating BBB-permeable) from the NPRL penetrated human BBB spheroid cells. No evidence suggests that ferulic acid or five BBB-impermeable compounds (Pred scores < 1.29423E−05, which designate compounds that pass through the human BBB) can pass through the spheroid cells of the BBB. Our validation of in vitro experiments indicated that the in silico prediction of small-molecule permeation in the BBB model is accurate. Transformer-based models like MegaMolBART, leveraging the SMILES representations of molecules, show great promise for applications in new drug discovery. These models have the potential to accelerate the development of novel targeted treatments for disorders of the central nervous system.

## Introduction

The blood–brain barrier (BBB) is a customized capillary bed that separates the brain from the circulatory system. It can protect the brain from pathogens, such as bacteria and viruses^1–4^. BBB-penetrating drugs are commonly used to treat central nervous system (CNS) disorders, such as Alzheimer’s disease, Parkinson’s disease, amyotrophic lateral sclerosis, brain tumors (glioblastoma), and CNS infections (e.g., *Neisseria meningitides* infection) using antibiotic agents, such as meningitis agents^1,5–7^. The BBB, with tight junction and efflux transporter proteins, prevents the entry of therapeutic agents into the brain, resulting in unsuccessful therapy for brain and CNS diseases^8–10^. Alternatively, compounds with targets in peripheral tissues should be investigated for their BBB permeability to prevent CNS adverse drug reactions, such as drowsiness, respiratory depression, nausea, vomiting, dizziness, trance, and anxiety^11^. Through the development of this model and rapid screening of the compound database, new compounds for treating CNS diseases can be developed, and unknown compounds can be predicted for absorption, distribution, metabolism, excretion, and toxicity^12–17^.

Developing a practical and accurate model for predicting the BBB permeability of compounds is important for brain and neuron therapeutic new drug discovery in silico^13,18^. These compounds have known BBB permeable compounds. A widely used database is LightBBB, which contains 7162 compounds with 5453 BBB permeable compounds (BBB +) and 1709 BBB impermeable compounds (BBB-)^19^. These 1155 compounds had Log*BB* (logarithm of drug concentration in the brain by the concentration in the blood) values (accession date: 2/20/2023). Another database is B3DB, which includes 7807 compounds with 4956 BBB permeable compounds (BBB+) and 2851 BBB impermeable compounds (BBB−), and the 1058 compounds are with Log*BB* values^20^. LightBBB has been included in the new B3DB database. DeePred-BBB collects 3605 compounds, including 2607 BBB permeable compounds (BBB+) and 998 BBB impermeable compounds (BBB−)^21–24^.

Inspired by natural language processing, transformer-based architectures for solving chemo-informatics tasks have become increasingly popular in recent years^25–27^. Because chemical structures are in a simplified molecular input line entry system (SMILES) format, they are similar to their own language^28^. Thus, SMILES strings can be trained using transformers for transformer models to learn different characteristics of chemical data, such as chemical properties and its structures^28–31^. Chemical data are often complex and high-dimensional, making it difficult to train a model from scratch using limited data^28^. Pre-training on abundant data using techniques that do not require labeling, such as pre-training through the use of auto-encoders, can help the model learn general representations that can be transferred to downstream tasks, leading to improved performance and faster convergence^32–34^. MegaMolBART^35^ is a small-molecule language model pre-trained using a bidirectional and autoregressive transformer (BART) architecture on the ZINC-15 dataset^36^. The encoder of the model can be used to extract molecular features for down-stream predictive models. MegaMolBART was implemented using NVIDIA’s NeMo Toolkit, which is a Python framework agnostic toolkit for creating artificial intelligence (AI) applications through reusability, abstraction, and composition^35^. The MegaMolBART framework is open source and extends the NeMo Toolkit’s functionalities to add chemistry-specific functions, such as SMILES masking and RDkit functionalities for training augmentation^37,38^. Previous research on predicting blood–brain barrier (BBB) permeability for small molecules has employed various features and machine learning techniques^11,20,39^. Physicochemical properties were calculated using software toolkits like Dragon and PaDEL^40,41^. Additionally, molecular fingerprints, substructure fingerprints, and 2D compound images generated by the RDKit package were utilized as input features^42,43^. These features were then used to train both traditional machine learning algorithms such as support vector machines (SVMs)^44,45^, k-nearest neighbors (kNNs)^46,47^, random forests^48,49^, and naive Bayes classifiers^50–52^, as well as deep learning methods including dense neural networks (DNNs)^53,54^, 1D convolutional neural networks (CNNs), and 2D CNNs^21,38,55^.

In this study, we hypothesized that a deep learning model can provide a quick method to determine if a novel compound design can cross the BBB. To achieve this, we used MegaMolBART as the SMILES encoder to identify if a molecule passes through the BBB. We compared the results with those of traditional molecular similarity methods called fingerprinting. Here, we use Morgan or Circular Fingerprints which apply the Morgan algorithm to a set of atom invariants^56,57^. We will also verify these results using newly created natural product compound libraries that are not currently included in any database, such as the Compound Library of the Natural Products Research Laboratories (NPRL) of China Medical University Hospital (CMUH) in Taiwan^58^. Furthermore, an in vitro liquid chromatography and mass spectrometry (LC–MS/MS) study was conducted to assess the integrity of BBB spheroids and the permeability of compounds from NPRL.

## Results and discussions

Supplementary Figure S1 shows the training and validation loss curves of training with PyTorch using the MegaMolBART embedding connected to the MegaMolBART encoder and then connected to a classifier layer. The training showed that the loss converged quickly, with over-fitting occurring at approximately 400 epochs. Supplementary Figure S2 shows the validation best area under curve (AUC) with and without the exponential moving average (EMA); the occurred immediately before the model started to over-fit (from the loss curve). We also tested different sizes of MegaMolBART, with training on the CMUH-NPRL test set with B3DB dataset (Supplementary Table S1), and B3DB test set with CMUH-NPRL dataset (Supplementary Table S2). These models exhibited validation AUC curves similar to those shown in Supplementary Fig. S2.

Since we believe that the small dataset caused MegaMolBART to over-fit the BBB datasets, we believe that the model did not take full advantage of the pre-training done on the ZINC-15 dataset. Thus, we attempted to use regression with XGBoost. When using regression, we compared it with the Morgan Fingerprints generated using RDKit with 2048 as the number of features^37–39^. The regression results are shown below. We also examined the accuracy of the results by converting the predicted Log*BB* value into accuracy using the formula shown in the previous section. The results of the regression with XGBoost in Supplementary Fig. S3 show that MegaMolBART embeddings work significantly better compared to Morgan Fingerprints, with the larger model showing the best performance. However, when the computed accuracy was compared using the predicted Log*BB*, the Morgan fingerprints performed slightly worse compared to the MegaMolBART embeddings. As the classification performed worse, the data distribution was checked using t-distributed stochastic neighbor embedding (t-SNE) on the NVIDIA GPU Cloud (NGC) MegaMolBART embeddings. The t-distributed stochastic neighbor embedding (t-SNE) distribution results shown in Supplementary Fig. S4 are that the data with Log*BB* are closely grouped together, whereas the data without Log*BB* are more spread out. This indicated that more data without Log*BB* were required to train a better model. Finally, we train the model with the XGBoost classifier using only the B3DB dataset. The results shown in Supplementary Fig. S5 indicate a significant improvement in the test dataset. However, this model was applied to the CMUH-NPRL dataset, the accuracy decreased by approximately 50%.

**Figure 1:**
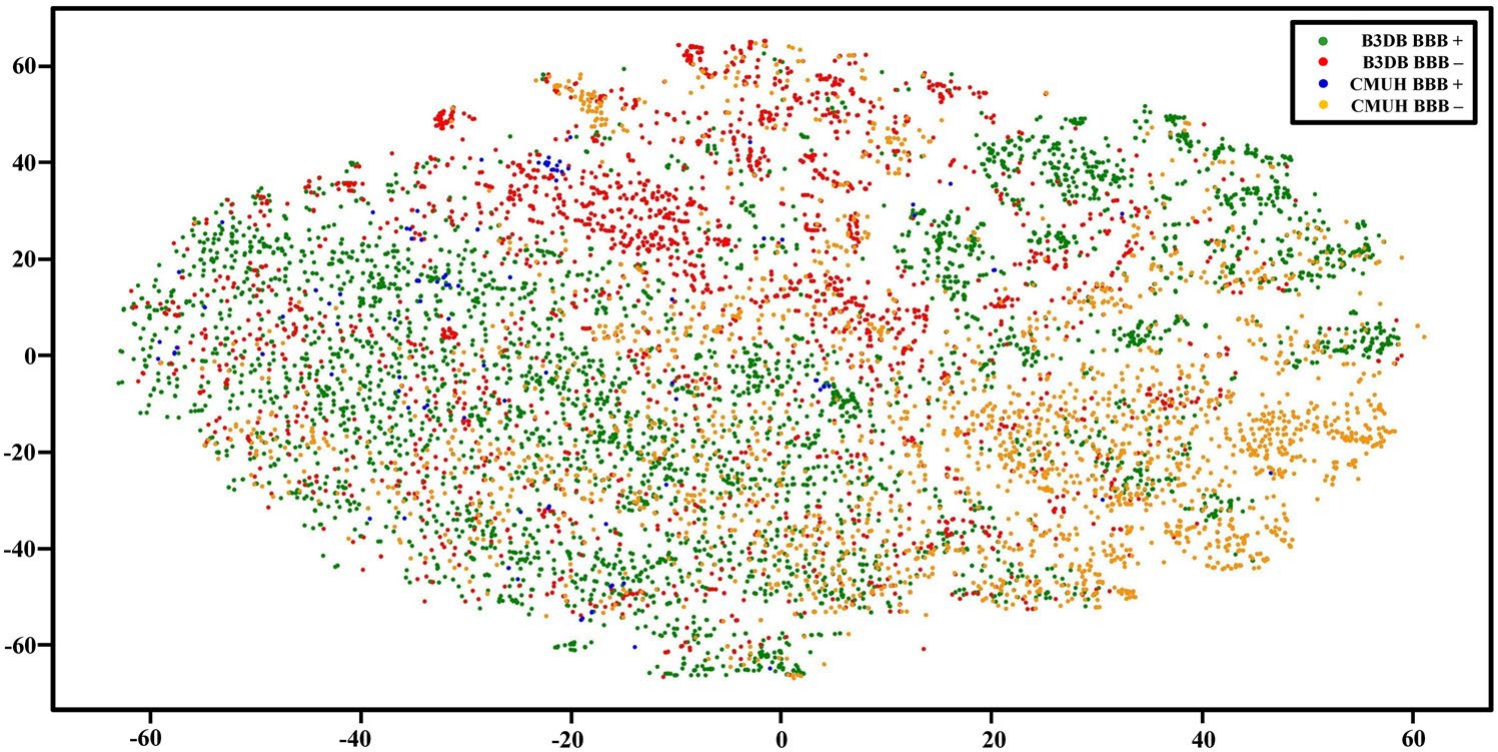
Data distribution of the molecule embeddings visualized using the t-distributed stochastic neighbor embedding (t-SNE) color coded by dataset and BBB+/BBB−.

**Figure 2:**
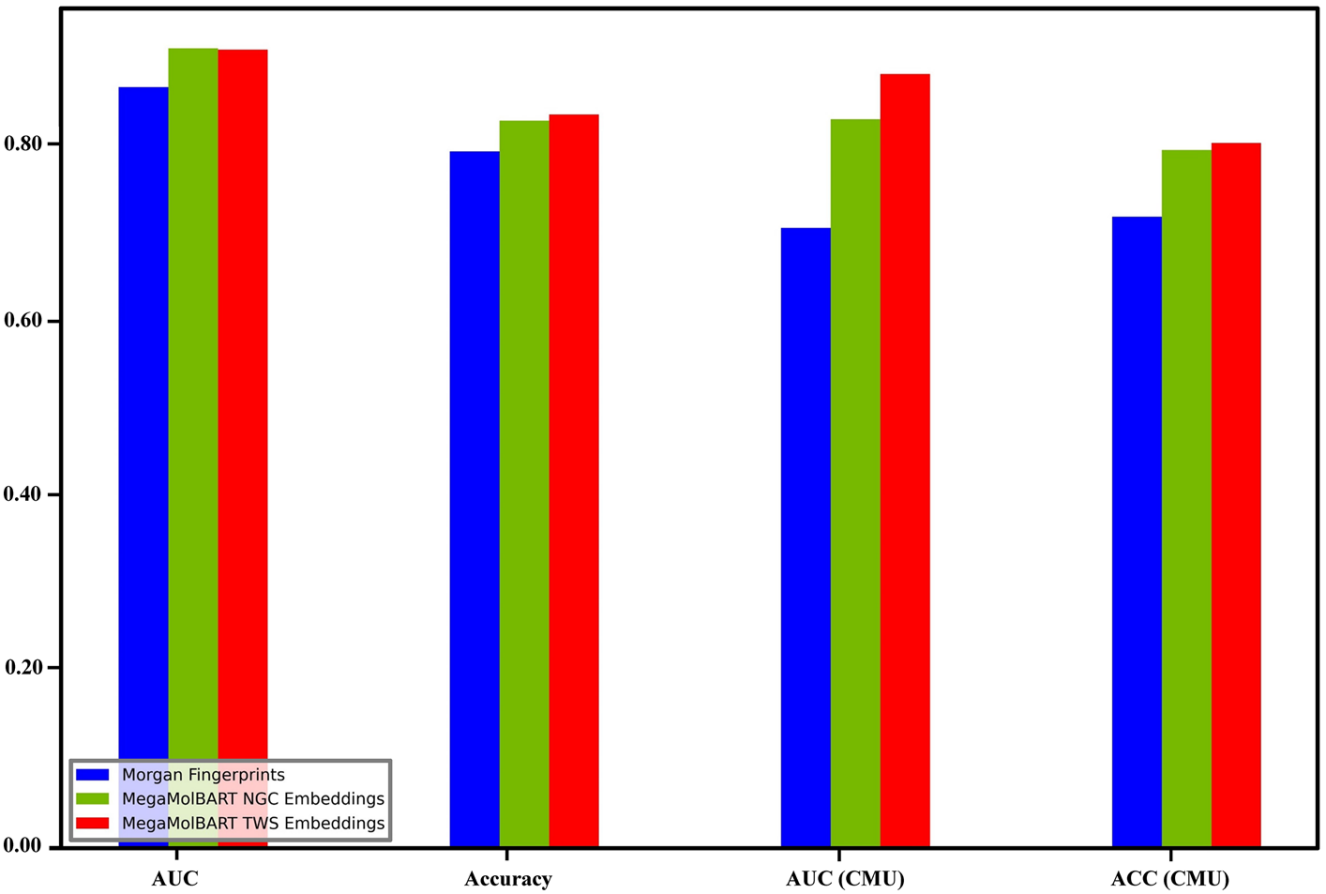
Classification AUC and accuracy of the test set from B3DB and CMUH and classification AUC and accuracy of only the CMUH test set.

Next, we checked the distribution of the CMUH and B3DB data. Figure 1 show the t-distributed stochastic neighbor embedding (t-SNE) applied to the CMUH-NPRL and B3DB data using the NVIDIA GPU Cloud (NGC) embeddings. Our results clearly shows that the CMUH-NPRL and B3DB data are distributed far apart; therefore, the next model would involve mixing both types of datasets together for training. Finally, using 80% of both datasets for training, 10% of both datasets for validation, and 10% of both datasets for testing, we achieved an AUC of 0.88 using MegaMolBART. We also compared the same classifier with the Morgan Fingerprints and found a significant difference between the Fingerprints and Embeddings, with the larger MegaMolBART model performing slightly better (Fig. 2). Furthermore, we performed a comparative analysis of previous machine learning models that use physicochemical properties of molecules for BBB permeability classification and our MegaMolBART transformer-based. The traditional machine learning models used were the LightGBM mentioned in the LightBBB paper^19^ and DNN in the DeePred paper^21–24^. Both were trained using various physicochemical properties of the molecules, including molecular weight, lipophilicity, and hydrogen bonding potential and polar surface area, calculated using Dragon software^59^ and PaDEL^60^ respectively. Our MegaMolBART transformer-based model is a variant of the BART transformer architecture, adapted for BBB permeability classification using the SMILES representation of molecules. The model was pre-trained using the ZINC-15 database, and the BERT encoder was used to transform molecules into embeddings, which were then used to train a large dataset of molecules with known BBB permeabilities and optimized using a combination of gradient descent and back propagation. For the comparative analysis, we used the datasets provided by the respective papers, analyzed the datasets using their described tenfold cross-validation method, and reported the AUC for comparison.

The results of our comparative analysis showed that on the LightBBB dataset, the AUC of our model was 0.93 compared to the LightBBB reported AUC of 0.94 (Supplementary Table S3). For the DeePred dataset, the AUC of our model was 0.96 compared to the DeePred dataset, which reported an AUC of 0.99 (Supplementary Table S4). However, the transformer-based model does not require pre-computation of SMILE features using other software tools. Calculating physicochemical properties of molecules requires significant computational resources and can be time-consuming^61–63^. Moreover, many properties may not be easily interpreted or available for all molecules^64^. This means that these models may be unsuitable for large-scale drug discovery applications in which the number of molecules considered can be in the millions. In contrast, our MegaMolBART transformer-based model, can handle large and diverse sets of molecules without requiring extensive feature engineering or computationally intensive calculations (Supplementary Fig. S6). SMILES is a widely used standard for representing molecular structures as strings of characters that can be easily input into a transformer-based model^65–69^. Furthermore, using SMILES allows for greater flexibility and generalization of the input data because it can capture various molecular structures and properties^66,67,70^. This makes the transformer-based models more robust and adaptable to new and diverse sets of molecules, which are critical for new drug discovery^71–73^. Another advantage of transformer-based models is their ability to learn complex patterns and relationships in the input data, which may not be easily captured through calculations of physicochemical properties or fingerprints^64,74^. Transformers use a self-attention mechanism that allows them to selectively attend to different parts of the input sequence and capture long-range dependencies and complex relationships among different parts of the SMILES sequence^75–77^.

**Figure 3:**
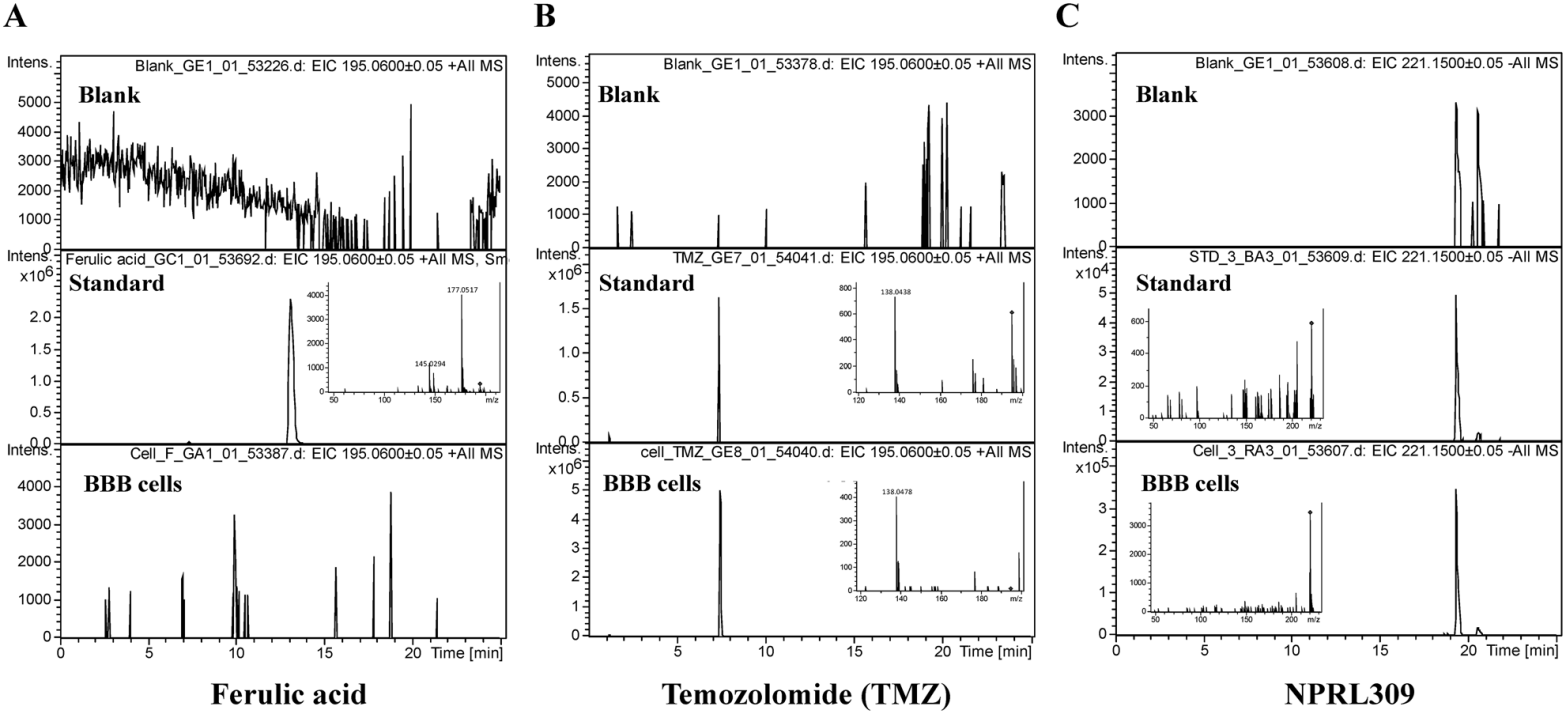
Human BBB spheroid cells were analyzed by LC–MS/MS, which shows that TMZ, ferulic acid, and NPRL-309 have standard peaks.

Using LC–MS/MS to assess BBB integrity has become an advanced technology in recent years^78–80^. We used LC–MS/MS on human BBB spheroid cells (consisting of human brain microvascular endothelial cells, brain vascular pericytes, and astrocytes) to analyze BBB permeability in vitro. We selected, at random, 21 (Pred scores = 1, indicating BBB-permeable compounds) and five (Pred scores < 1.29423E−05, indicating BBB-impermeable compounds) compounds of NPRL to be verified in vitro. Figure 3 and Supplementary Fig. S9 demonstrate TMZ and 21 BBB permeable compounds (BBB +) (predicted by NVIDIA’s NeMo Toolbox to be BBB-permeable) of NPRL penetrated human BBB spheroid cells. Ferulic acid and five BBB-impermeable compounds (Pred scores < 1.29423E−05) predicted by the NPRL were inaccessible to human BBB spheroid cells. To the best of our knowledge, this was the first study on the BBB permeability of compound libraries using abundant databases. Our method offers a novel cellular model for BBB permeability measurements. The results summarized in Table 1 provide evidence that the BBB permeable compounds (BBB+) of NPRL, predicted by NVIDIA’s NeMo Toolkit, can penetrate human brain microvascular endothelial cells and reach human BBB spheroid cells. The permeability coefficients validated these findings. The Natural Products Research Laboratories (NPRL) compound library was established by Professor Kuo-Hsiung Lees (The University of North Carolina at Chapel Hill) from China Medical University Hospital (CMUH) to determine the bioactivity of these treasured natural products and their synthesized derivatives^58,81^. Our research provides a fast and highly specific in silico and in vivo methods and a new bioactivity assay for NPRL compounds. This study provides a novel research method for building platforms for compound laboratories with large databases. In the future, we aim to use a human brain endothelial cell model (hCMEC/D3 human BBB cells) to further explore molecular and pharmacologic transport mechanisms of novel compounds entering the BBB^82^.

**Table 1:** In vitro permeability assay and in silico prediction outcomes for BBB spheroid cells.

Sample number	Sample	In silico prediction		In vitro study
		PRED SCORES	PRED LABEL	LC–MS/MS
Control	–	–	–	–
Negative control	Ferulic acid			BBB impermeable
Positive control	TMZ			BBB permeable
1	NPRL309	1.00	BBB +	BBB permeable
2	NPRL358	1.00	BBB +	BBB permeable
3	NPRL588	1.00	BBB +	BBB permeable
4	NPRL818	1.00	BBB +	BBB permeable
5	NPRL833	1.00	BBB +	BBB permeable
6	NPRL835	1.00	BBB +	BBB permeable
7	NPRL836	1.00	BBB +	BBB permeable
8	NPRL842	1.00	BBB +	BBB permeable
9	NPRL1089	1.00	BBB +	BBB permeable
10	NPRL1185	1.00	BBB +	BBB permeable
11	NPRL1188	1.00	BBB +	BBB permeable
12	NPRL1192	1.00	BBB +	BBB permeable
13	NPRL1195	1.00	BBB +	BBB permeable
14	NPRL1241	1.00	BBB +	BBB permeable
15	NPRL1958	1.00	BBB +	BBB permeable
16	NPRL2026	1.00	BBB +	BBB permeable
17	NPRL2029	1.00	BBB +	BBB permeable
18	NPRL2051	1.00	BBB +	BBB permeable
19	NPRL2059	1.00	BBB +	BBB permeable
20	NPRL2148	1.00	BBB +	BBB permeable
21	NPRL3767	1.00	BBB +	BBB permeable
22	NPRL2359	1.38848E−05	BBB−	BBB impermeable
23	NPRL2576	1.49735E−05	BBB−	BBB impermeable
24	NPRL2646	1.40275E−05	BBB−	BBB impermeable
25	NPRL3098	1.29423E−05	BBB−	BBB impermeable
26	NPRL3183	1.74123E−05	BBB−	BBB impermeable

Our study shows that pre-training can significantly accelerate the convergence of down-stream task models. The Large MegaMolBART pretrained on the ZINC-15 dataset shows the most promise and best accuracy on B3DB (Fig. 2), although more pre-training may be required to obtain a better accuracy score, and more Log*BB* data are required for a better regression accuracy score. The current distribution of the B3DB data is uneven. In addition, the classification of B3DB can reach up to 0.90 of AUC with our Taiwan Web Service (TWS) embedding and XGBoost regression (Supplementary Fig. S3). Classification can reach up to 90% AUC with TWS embeddings and XGBoost classification (Supplementary Fig. S5). The results of the classification can also been seen through the confusion matrices and evaluation metrics of the test set found in Supplementary Fig. S7. Additionally, in vitro experiments confirmed the accuracy of the in silico prediction of the small-molecule BBB permeation model (Supplementary Fig. S8). Our results in this studies demonstrated that the Transformer-based models that use SMILES representations of molecules offer several advantages over traditional machine learning models that rely on physicochemical properties. These advantages include greater computational efficiency, flexibility in handling diverse sets of molecules, and the ability to learn complex patterns and relationships from the input data. Supplementary Table S5 showed the raw data of MegaMolBART analysis on blood brain barrier (BBB) permeability of NPRL compounds. Therefore, these models are promising for drug discovery and can accelerate the development of new treatments for CNS disorders.

In conclusion, our study underscores the benefits of large language models like MegaMolBART over traditional machine learning approaches. A key advantage is the ability to predict blood–brain barrier (BBB) permeability directly from SMILES molecular representations, circumventing the need for additional physicochemical property calculations. Such calculations can be computationally expensive and time-consuming processes.

## Material and methods

### In silico study

For our dataset, we used a collection of molecules curated by Natural Products Research Laboratories (NPRL) from China Medical University Hospital (CMUH), which consisted of drugs approved by the Food and Drug Administration (FDA) that either cross or do not cross the BBB, with more than 512 characters removed and converted to their canonical forms. We also included an open source BBB database (B3DB) and similarly converted SMILES to their canonical forms (URL: https://github.com/theochem/B3DB). After preprocessing, the CMUH dataset consisted of 105 molecules that crossed the BBB (BBB+) and 2394 that did not (BBB−), whereas the B3DB dataset consisted of 4956 BBB+ molecules and 2851 BBB− molecules. First, we attached the MegaMolBART embedding and encoder layers to different classifiers in PyTorch, such as a linear and other 1D CNN-based classifiers. We pulled the pre-trained MegaMolBART model available on NVIDIA GPU Cloud (NGC)^35^ which was trained with data parallelism on 64 V100 GPUs (4 nodes × 16 GPUs) for eight epochs (approximately 160 k iterations or ~ 80 wall-clock hours), using a batch size of 32 molecules per GPU (micro batch) (URL: https://catalog.ngc.nvidia.com/orgs/nvidia/teams/clara/models/megamolbart). The Noam scheduler was used with peak learning rate values of 0.0005 and 8000 warm-up steps. FusedAdam optimization was used with the following parameters: beta 1 = 0.9; beta 2 = 0.999. Categorical cross-entropy loss is used to train the models. The model is trained using the ZINC-15 dataset. We experimented with different hyper-parameters, such as freezing the MegaMolBART parts and allowing them to undergo fine-tuning. For datasets, we split the B3DB into 80% training, 10% validation, and 10% testing and used the CMUH dataset as the test set, as well as combining both datasets with 80% + 80% train, 10% + 10% validation, and 10% + 10% testing. The results were all fairly similar, with the area under the receiver operating characteristic curve (AUC) ranging from 0.57 to 0.63. To improve the performance of the MegaMolBART model, we collaborated with the Taiwan Web Service (TWS) operated by ASUS, which operates the TAIWANIA-2 cluster. We obtained eight nodes × eight V100 GPUs for a total of 64 GPUs and ran the large MegaMolBART configuration, allowing every other configuration and dataset to be consistent with the one that had been pre-trained on NGC. We ran the model for approximately 1 week, which lasted for three epochs (compared to the eight epochs above). Finally, once we had the large MegaMolBART pre-trained model that was trained on TWS, we again attempted to combine the embedding and encoder layers into a classifier in PyTorch (URL: https://pytorch.org/), but we could not obtain results better than an AUC score of 0.63. From there, we took a step back and examined the different MegaMolBART downstream task resources and used an XGBoost regressor through the embeddings from MegaMolBART and compared with Morgan Fingerprints. For this portion of the study, we found that only 1058 samples in the B3DB dataset had Log*BB* values that could be used for the regression analysis. A Log*BB* value that is ≥ − 1 means that the molecule was able to cross the BBB. Supplementary Figure S6 shows the calculated Log*BB* values in our model.

$$LogBB = \, Log \, {C_{Brain}}/{C_{Blood}}$$

C_brain_: Concentration of the molecule in the brain, C_blood_: Concentration of the molecule in blood.

We connected an XGBoost Regressor to all three feature types: Morgan Fingerprints, NGC MegaMolBART Embeddings, and TWS MegaMolBART Embeddings. The B3DB dataset with log BB was divided into 80% training, 10% validation for early stopping, and 10% testing groups. The mean square error (MSE) and R-square (R2) values were calculated with the 10% test set, whereas the accuracy was calculated with the inferred Log*BB* of the 6749 samples without Log*BB* and the 2499 CMUH dataset and converted to BBB+ or BBB−, depending on the inferred Log*BB* value. Next, because we required more training samples, we used the existing pipeline of MegaMolBART embeddings and replaced the XGBoost Regressor with an XGBoost classifier. For the next experiment, we used all B3DB and CMUH datasets split into 80% training, 10% validation, and 10% testing.

### In vitro study

Supplementary Figure S8 shows the in vitro experimental design. ScienCellTM (cat. no. Cat. #SP3D-8768; ScienCell Research Laboratories, Inc., CA, USA) supplied normal human BBB spheroids consisting of human brain microvascular endothelial cells, brain vascular pericytes, and astrocytes in a 1:1:1 ratio to simulate intracellular interactions at the BBB. These spheroids consisted of human microvascular endothelial cells, brain vascular pericytes, and astrocytes. The spheroids were cultured in the 3D-BBB spheroid medium (3D-BBBSpM; Cat. #3D-8701) supplemented with 3D-BBB spheroids (3D-BBBSpS; Cat. #3D-8752), and fetal bovine serum (FBS; cat. #0010; ScienCell Research Laboratories, Inc., CA, USA), 100 U/mL penicillin, and 100 g/mL streptomycin in 96 well round bottom ultralow attachment plates (Corning; Cat. #CLS7007) under a humidified atmosphere with 5% CO_2_ at 37 °C^83^. Spheroids from normal human BBB were cultured in 96-well round-bottom ultralow attachment plates. Spheroid cells were treated with 10 g/mL of Temozolomide (TMZ; positive control), ferulic acid (negative control), and NPRL compounds. They were collected and washed twice with phosphate-buffered saline; subsequently, acetone precipitation was used to remove the detritus and centrifuged for 10 min at 12,000 rpm. The supernatant was collected and vacuum-dried. For the MS analysis, the sample was re-dissolved in 20 μL of a solvent containing MeOH/H2O/FA (1:1:0.001 v/v/v), and the supernatant was directly used for the LC–MS/MS analysis. With an orthogonal electrospray ionization (ESI) source, a UHPLC system (Ultimate 3000; Dionex, Germany) equipped with a C18 reversed‐phase column (2.1 × 150 mm, 3 μm, T3; Waters, Milford, MA, USA) was coupled to a hybrid QTOF mass spectrometer (maXis impact; Bruker Daltonics, Bremen, Germany). The initial flow rates were 0.25 mL/min of 99% for solvent A (0.1% formic acid) and 1% for solvent B (acetonitrile with 0.1% formic acid). A sample volume of 5 µL was injected. Within 1 min of the injection, the solvent B concentration was maintained at 1%, increased to 40% over 15 min, increased to 99% over 3 min, and maintained for 3 min before returning to its initial concentration for 4.5 min. The MS was operated in positive and negative ion modes with an *m/z* range of 50 ~ 1000 at 1 Hz. The capillary voltage of the ion source was set at + 3600 V and − 3000 V, and the endplate offset was 500 V. The nebulizer gas flow was one bar, and the drying gas flow was 8 L/min. A temperature of 200 °C was set for drying. The radiofrequency (RF) power in Funnels 1 and 2 was 200 Vpp. The RF for the hexapole was 200 Vpp and the low mass cutoff for the quadrupole was 100 m/z. A data-dependent analysis mode was used to obtain the data. The four most intense precursor ions were selected for the MS/MS analysis, excluded after two spectra, and released after 0.5 min. The total cycle time was 1.8–2.3 s^84,85^.

### Supplementary Information

Supplementary Information.

## Data Availability

All data generated or analyzed during this study are included in this published article.
