# Supplementary material for: Predicting blood–brain barrier permeability of molecules with a large language model and machine learning
Source: Sci Rep. 2024 Jul 9;14:15844. doi: 10.1038/s41598-024-66897-y (PMC11233737; doi:10.1038/s41598-024-66897-y)
Supplement: Supplementary file 1 — Supplementary Information. [file 41598_2024_66897_MOESM1_ESM.pdf]

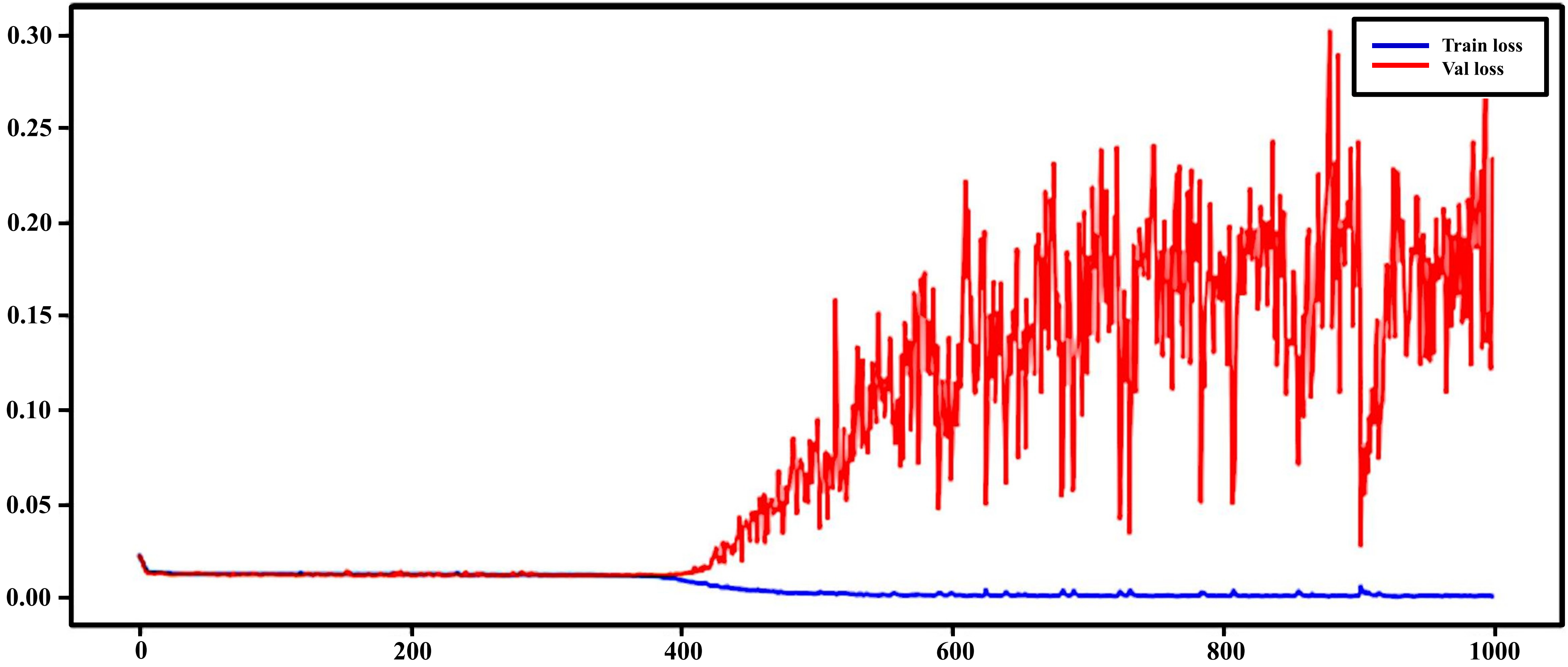

Supplementary Figure S1: Training and validation loss curves.

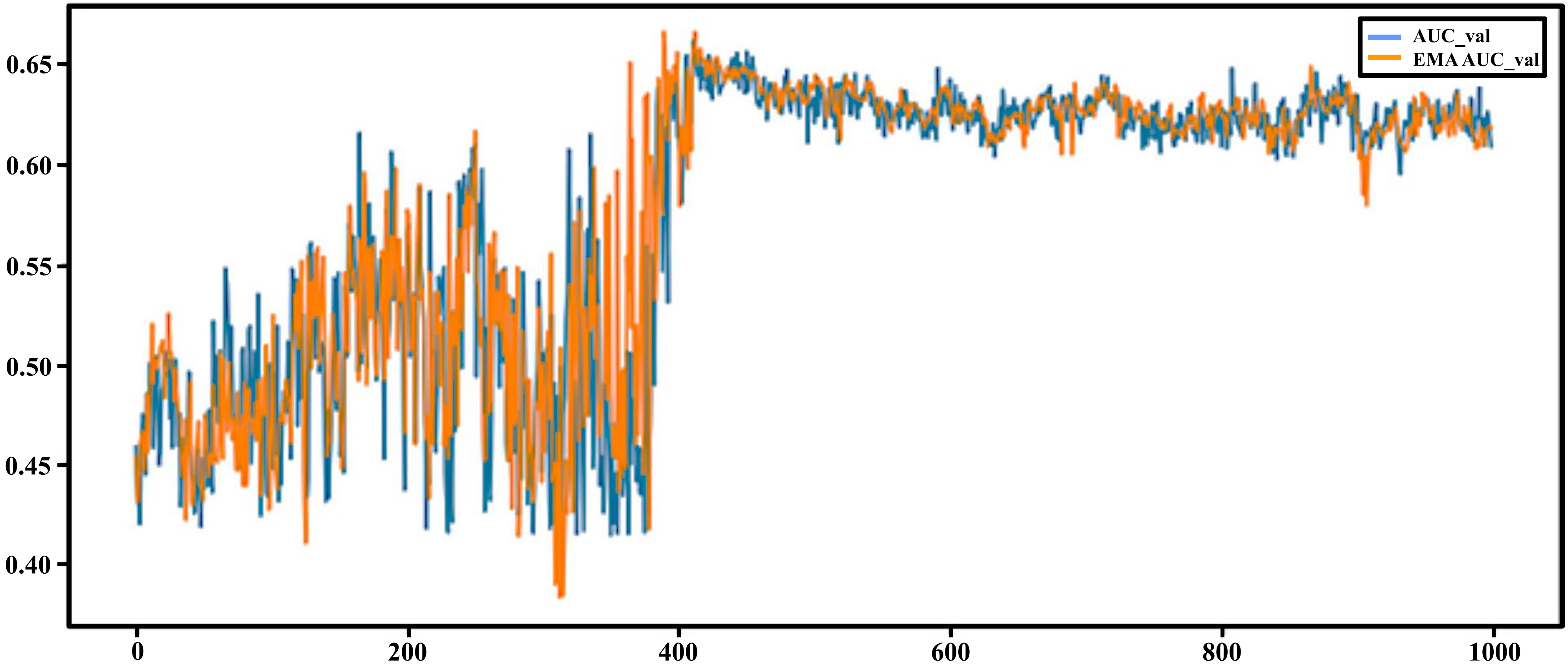

Supplementary Figure S2: The validation area under curve (AUC) during training with and without the exponential moving average (EMA).

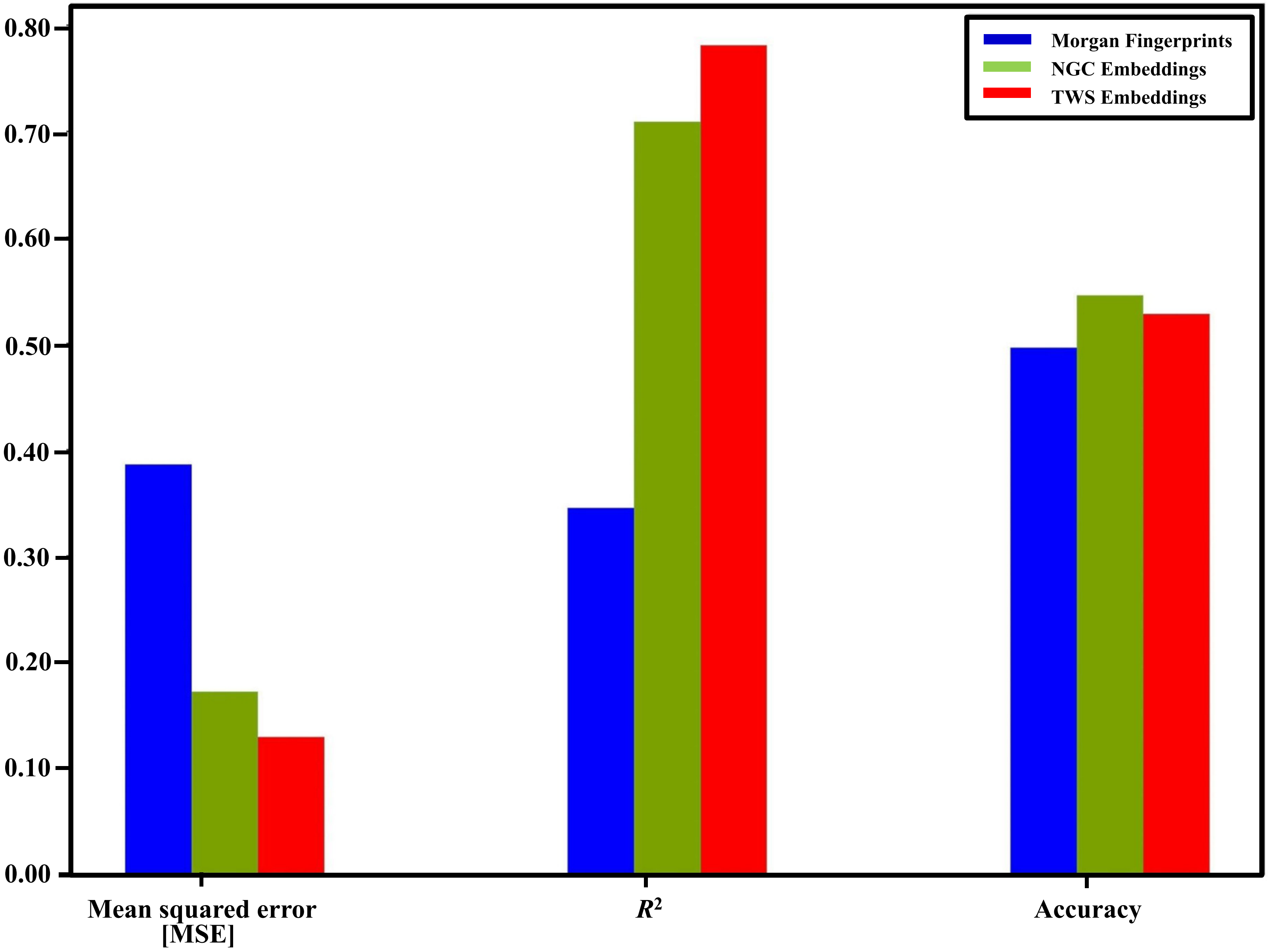

Supplementary Figure S3: Mean square error (MSE), R^2 and accuracy of performing regression with Log*BB* and accuracy of the conversion from Log*BB* to BBB+ and BBB-.

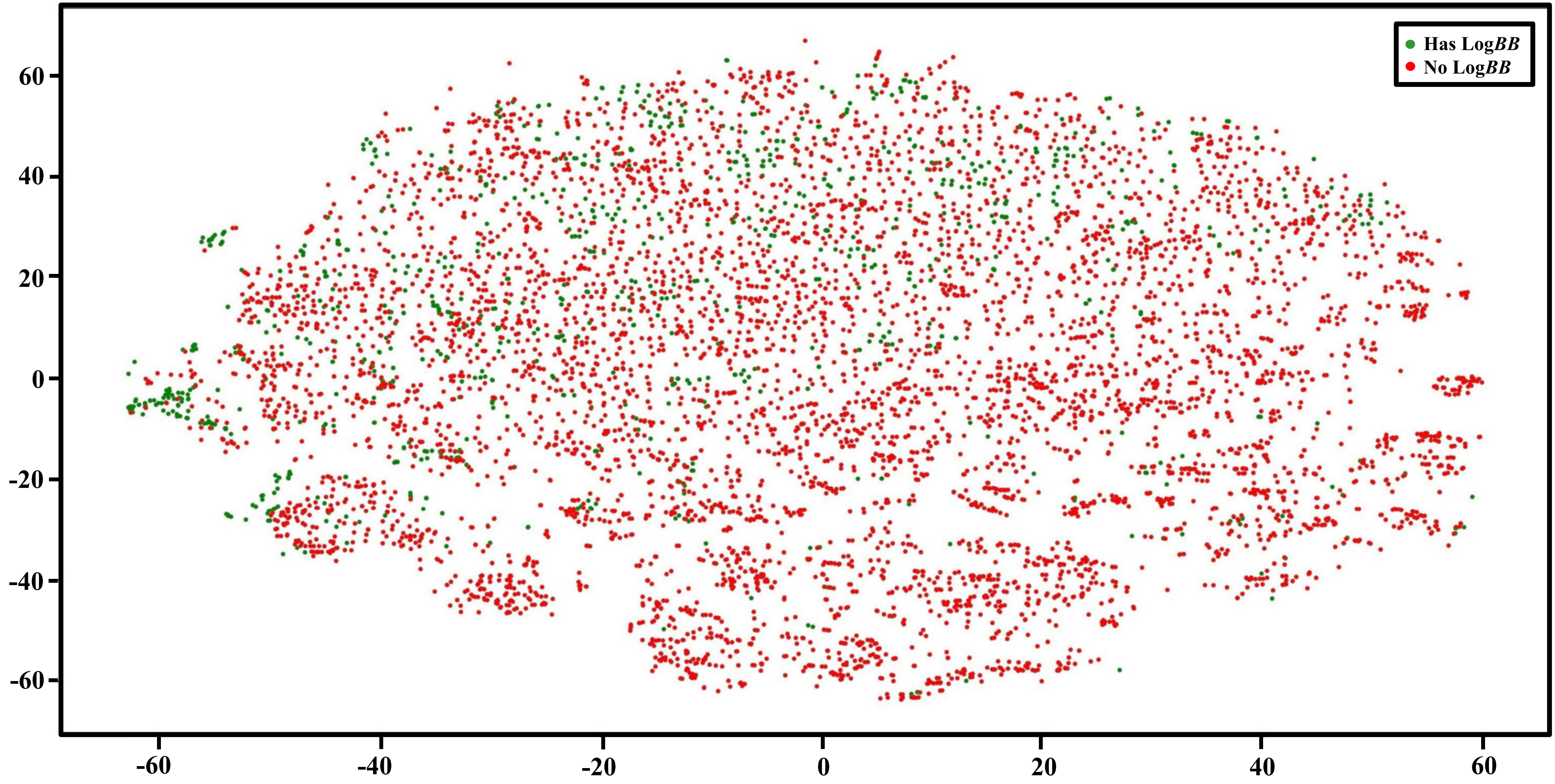

Supplementary Figure S4: Data distribution of the datasets visualized using t-distributed stochastic neighbor embedding (t-SNE) .

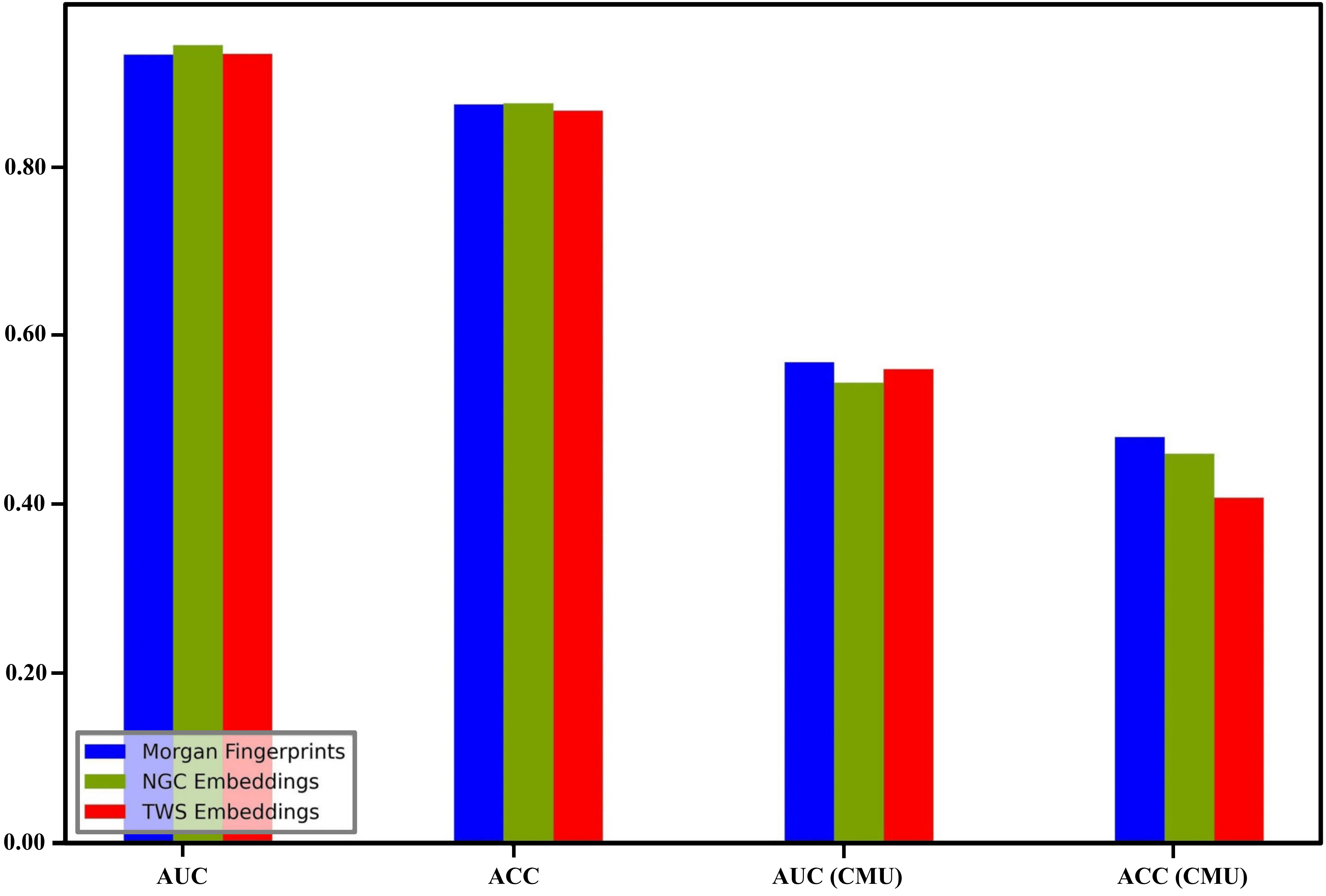

Supplementary Figure S5: Classification AUC and accuracy of the test set from B3DB and classification AUC and accuracy of the CMUH dataset.

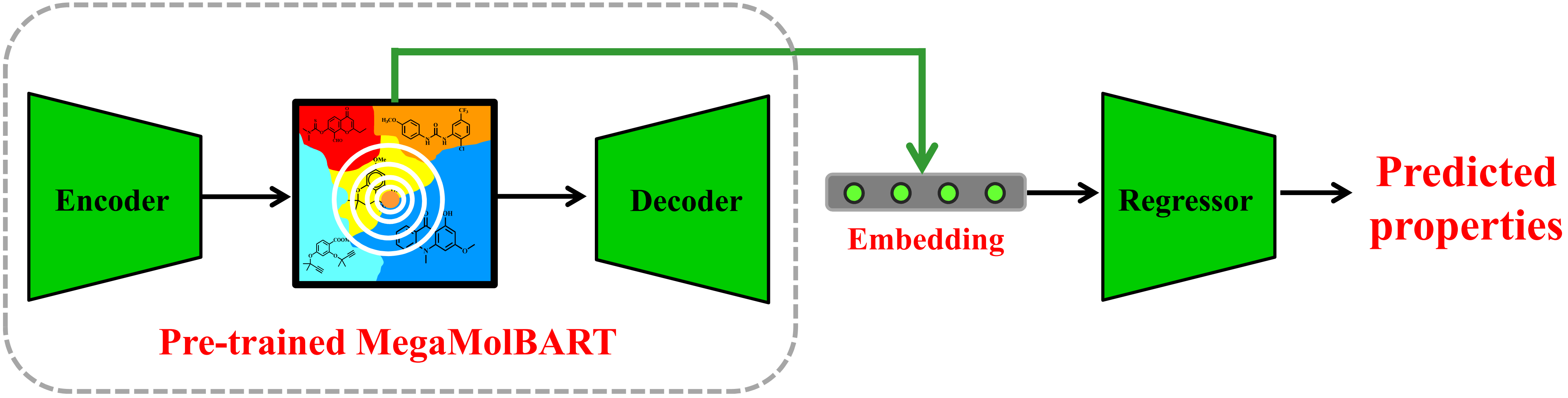

Supplementary Figure S6: Predicted properties of pipeline using large language model (LLM).

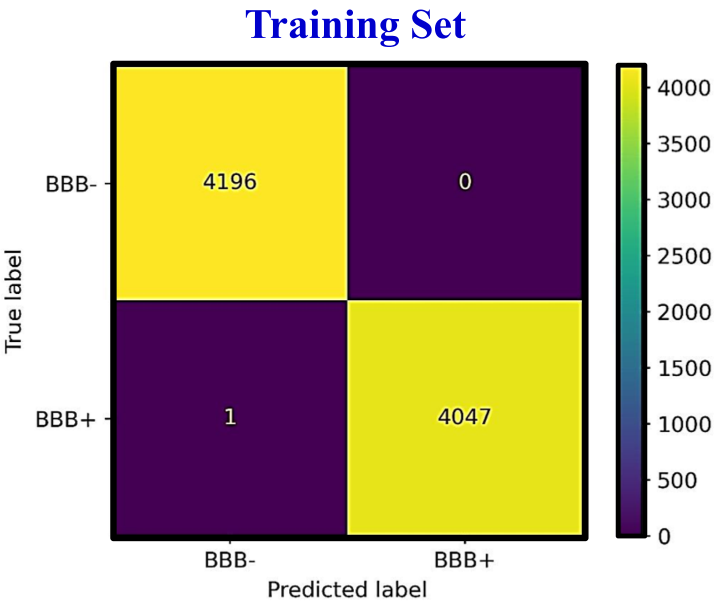

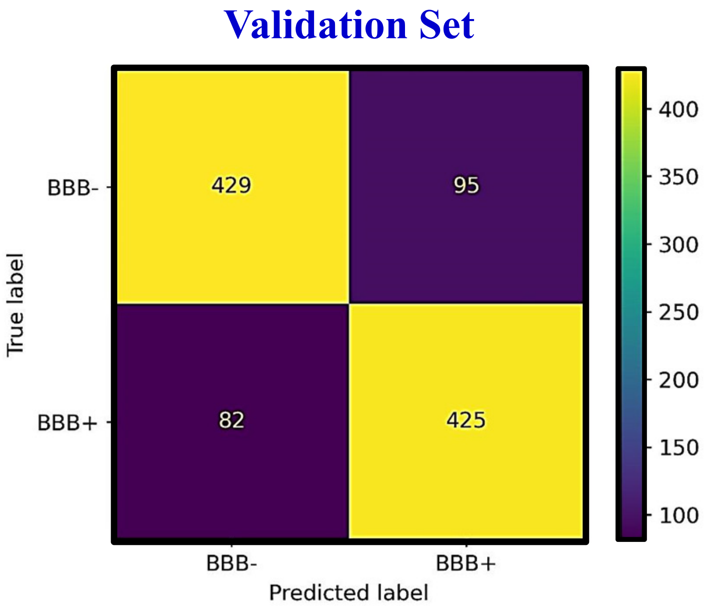

Test Set	
**Metric**	**Value**
AUC	0.90
Accuracy	0.83
Precision	0.83
Recall	0.83
F1-Score	0.83

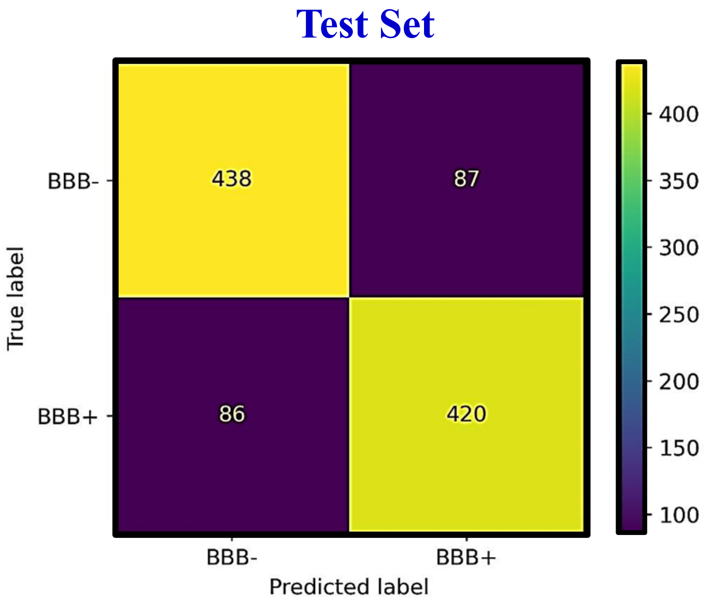

Supplementary Figure S7: The confusion matrices and evaluation metrics of the test set.

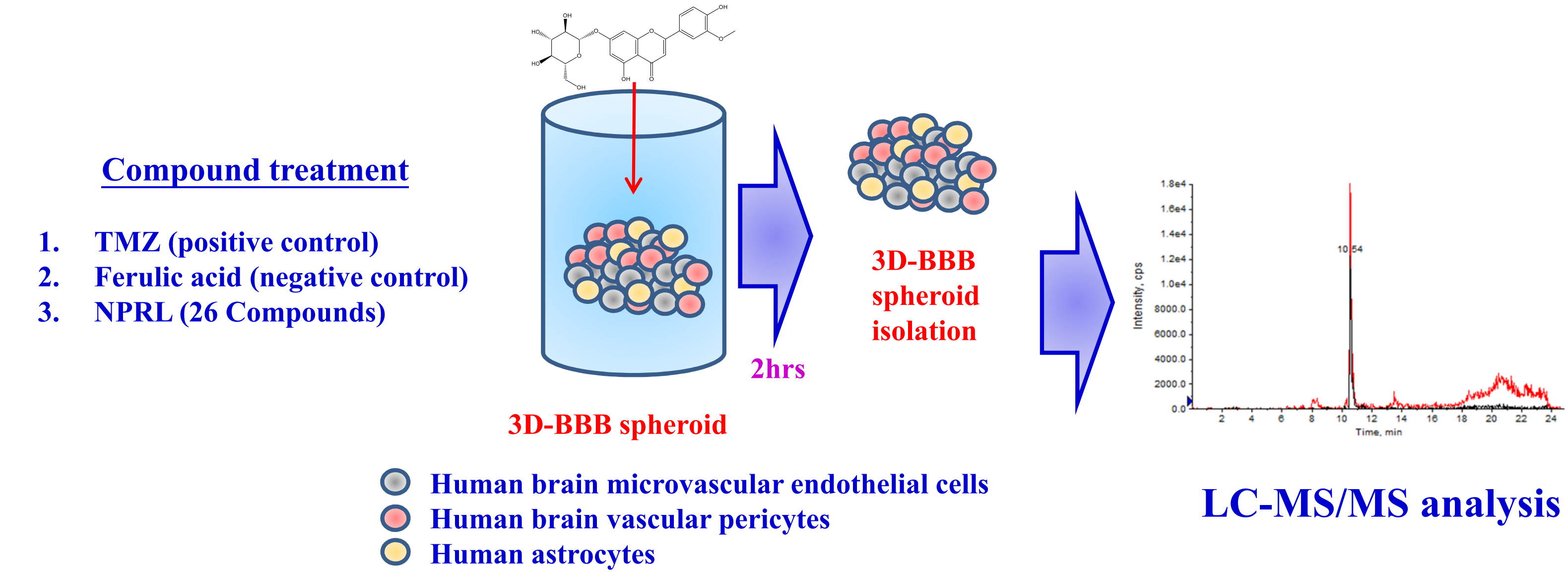

Supplementary Figure S8: A schematic diagram of *in vitro* experimental design.

Supplementary Figure S9. Human BBB spheroid cells were analyzed by LC-MS/MS.

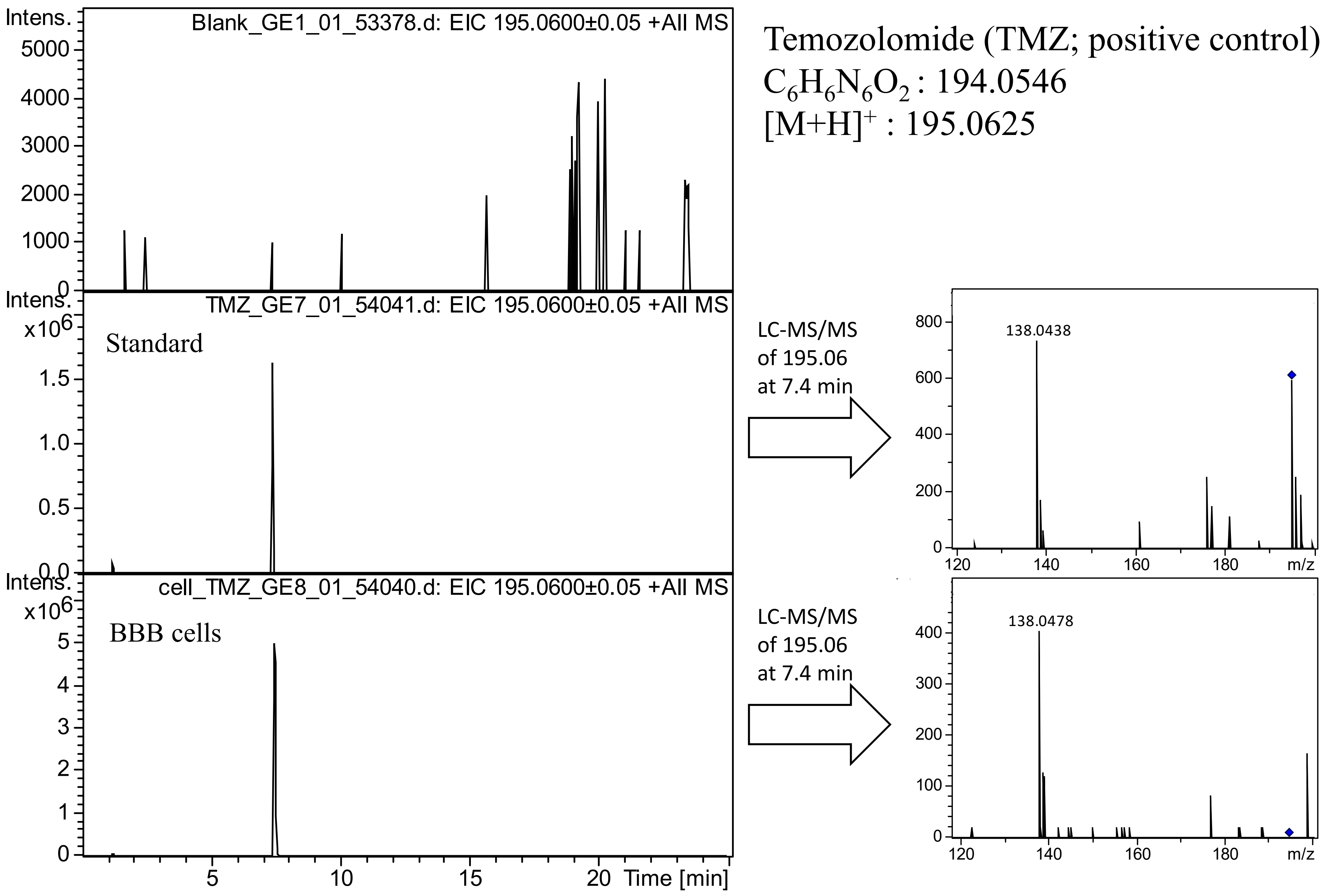

Ferulic acid (Negative control)

$C_{10}H_{10}O_4$: 194.0573

$[M+H]^+$: 195.0651

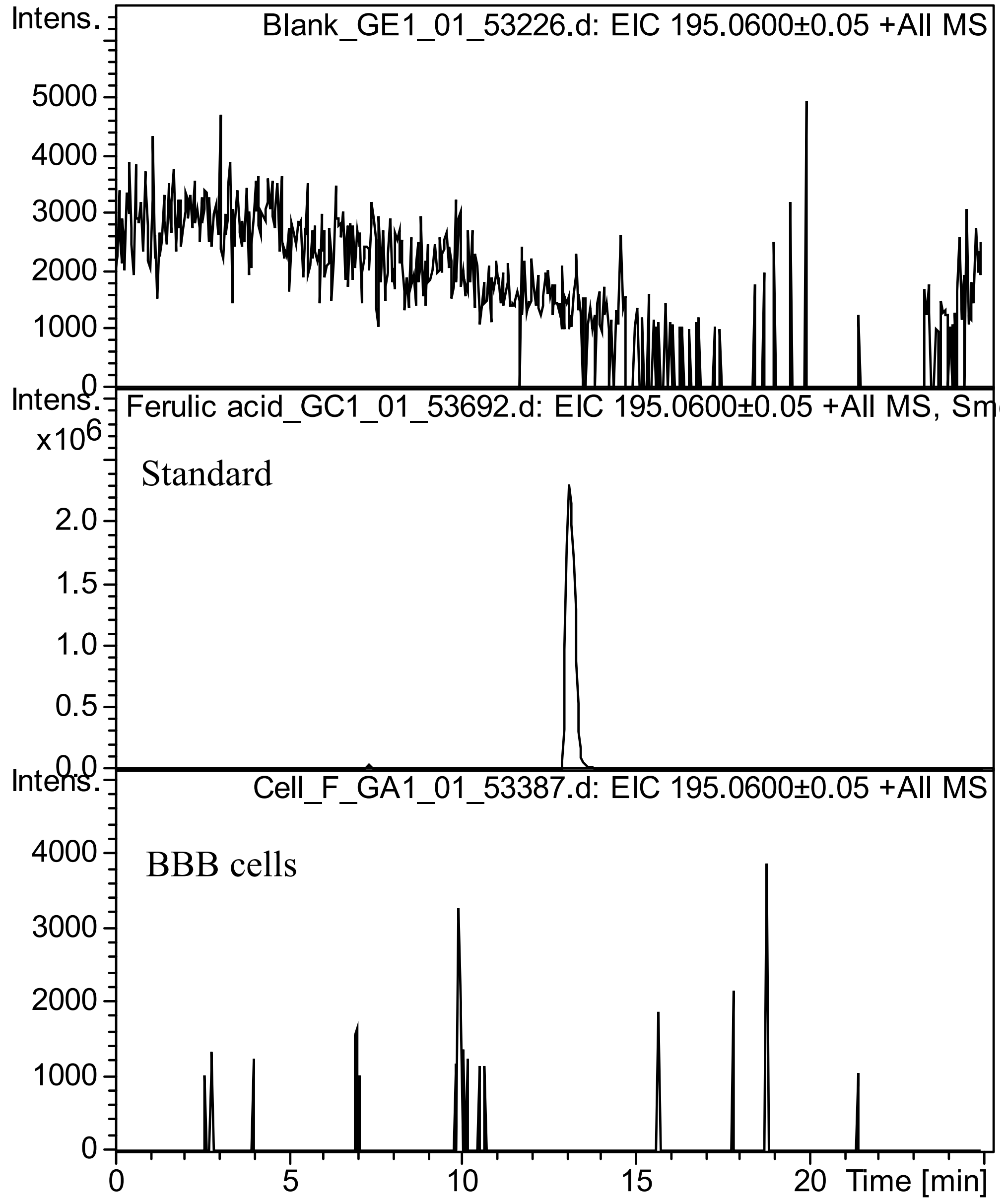

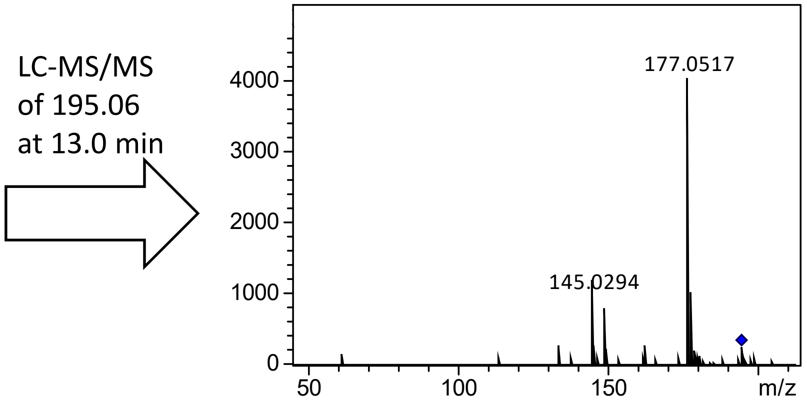

NPRL 309
[M-H]⁻ : 221.15

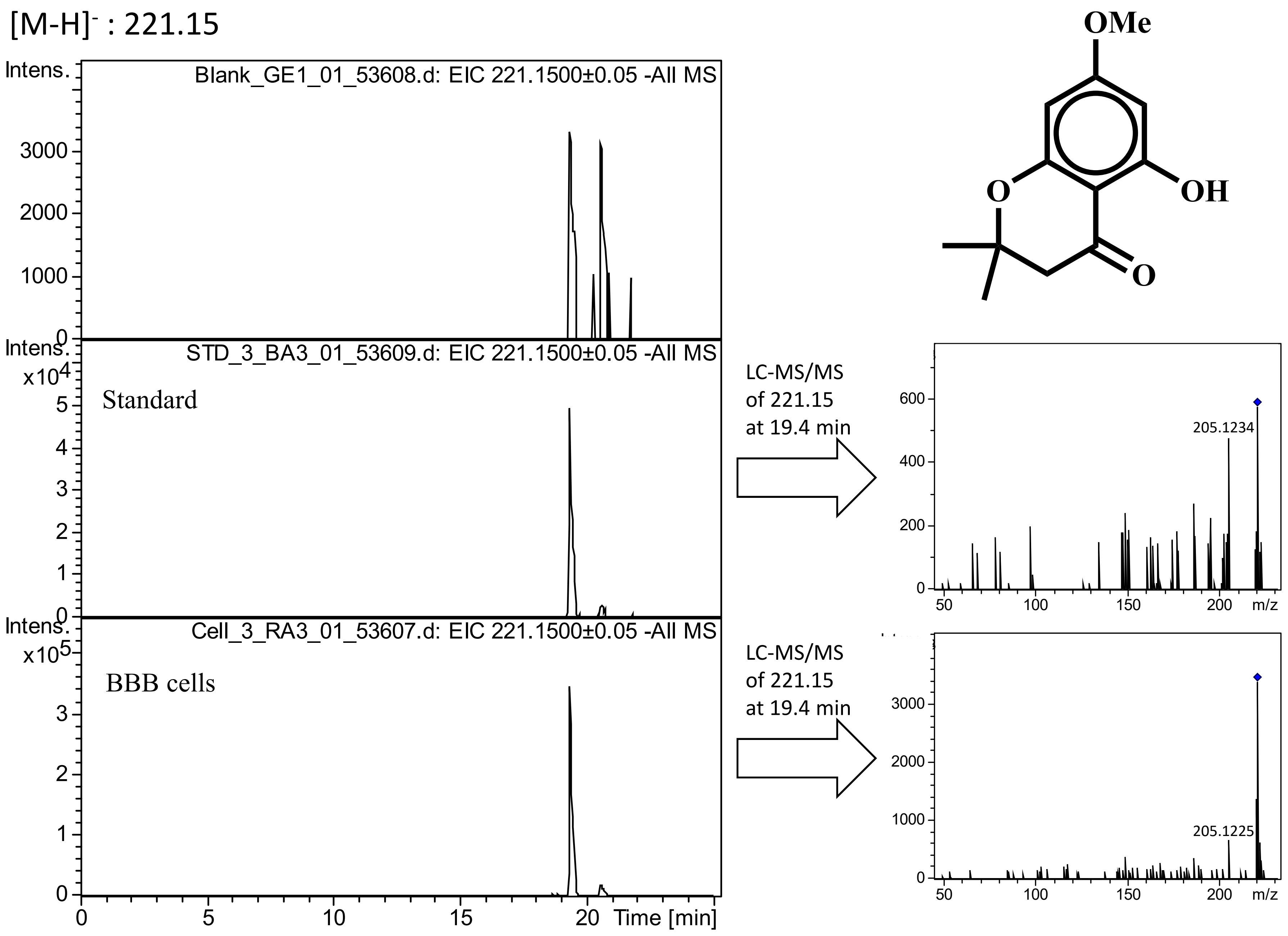

NPRL 358

$[M+H]^+$: 345.05

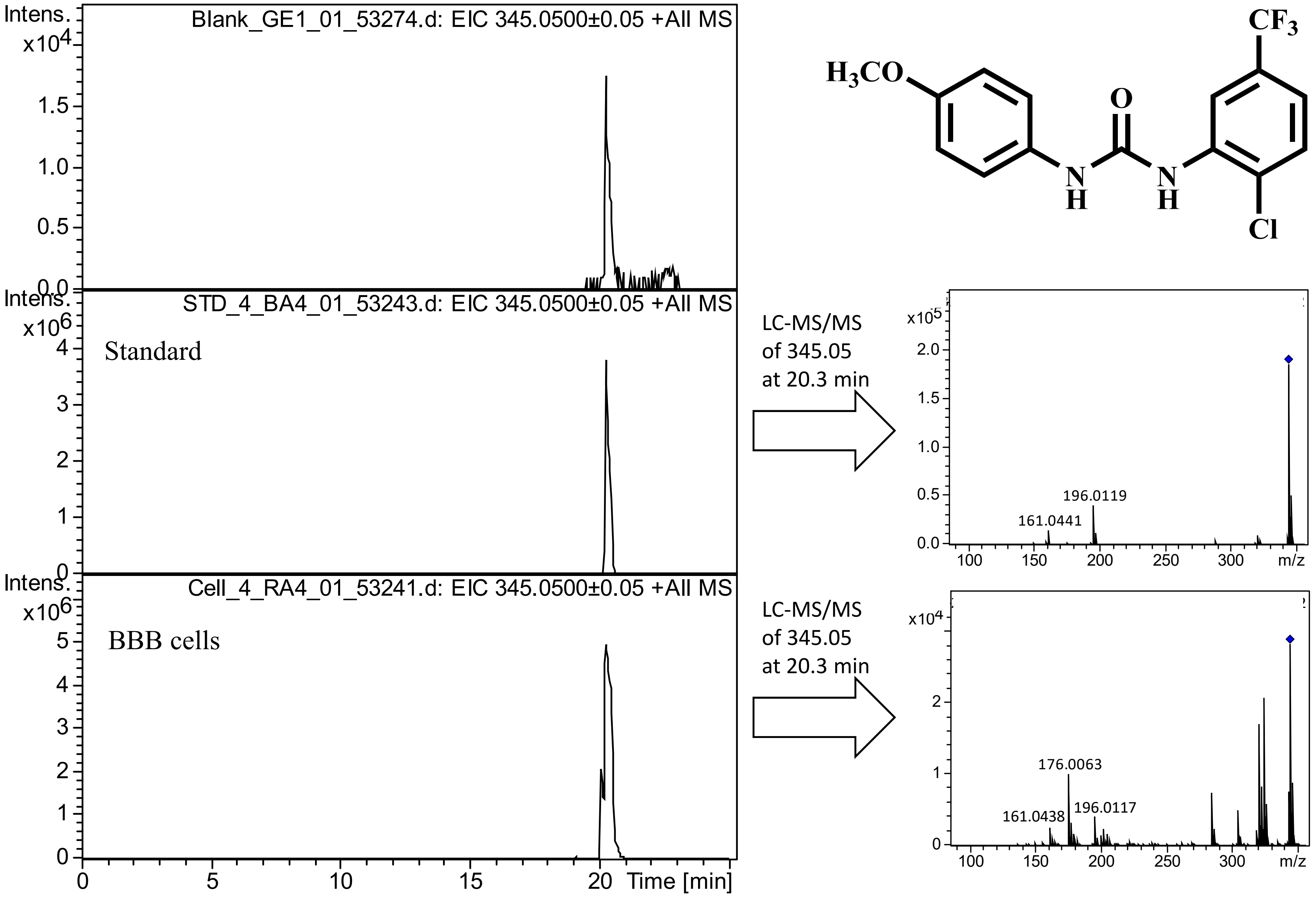

NPRL 588

$[M+H]^+$: 234.07

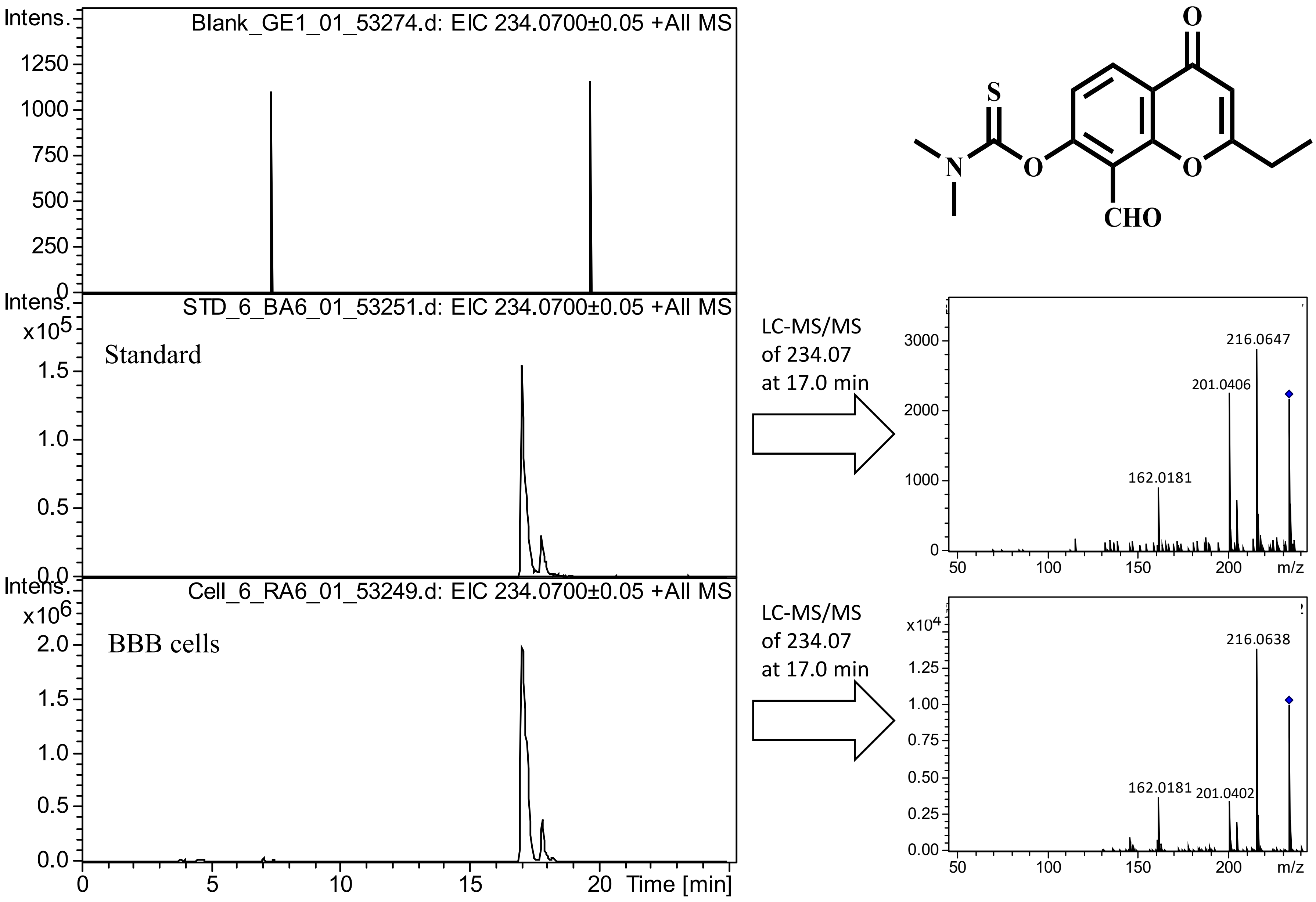

NPRL 818

[M+H]+ : 679.23 (340.12)

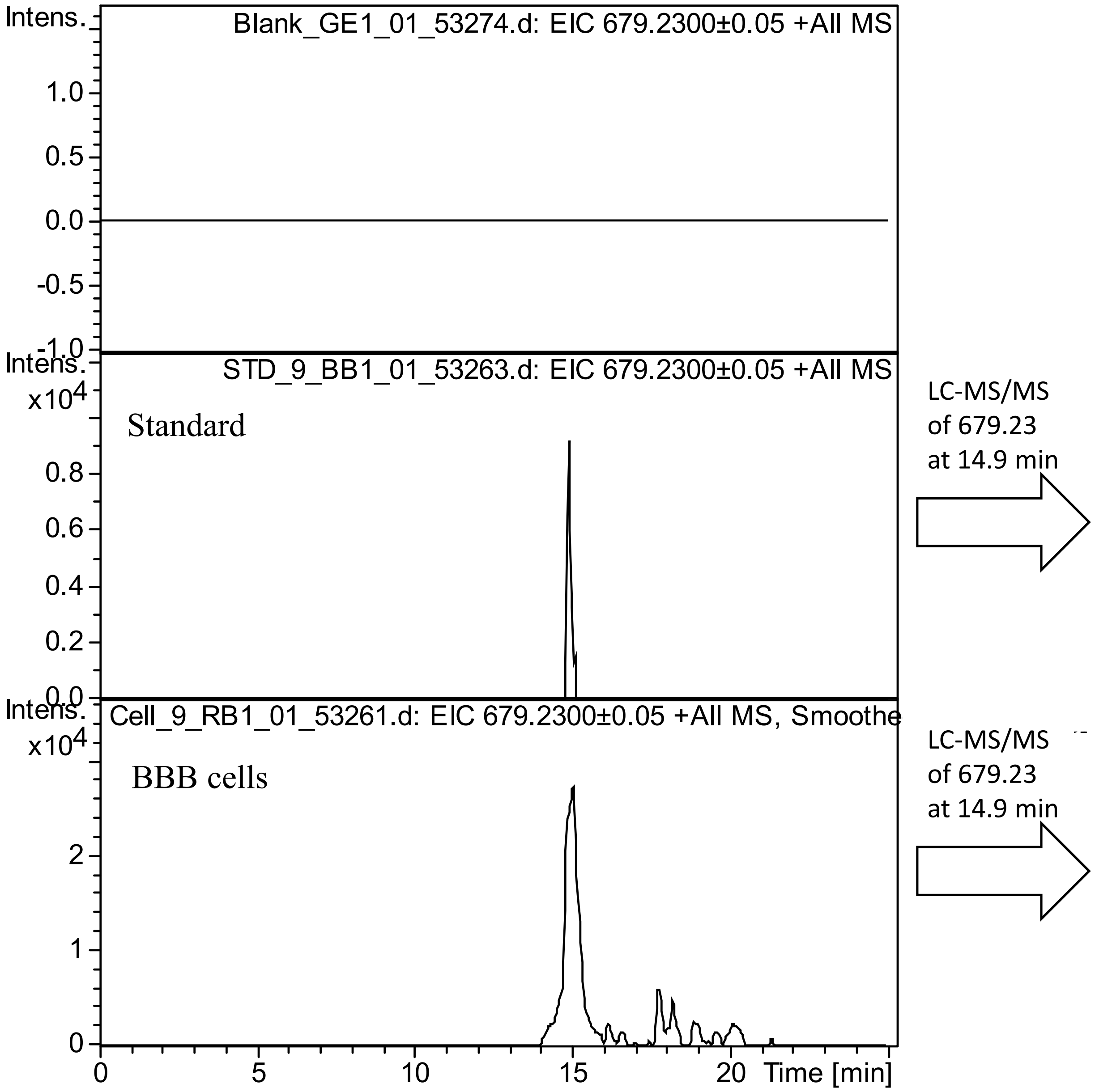

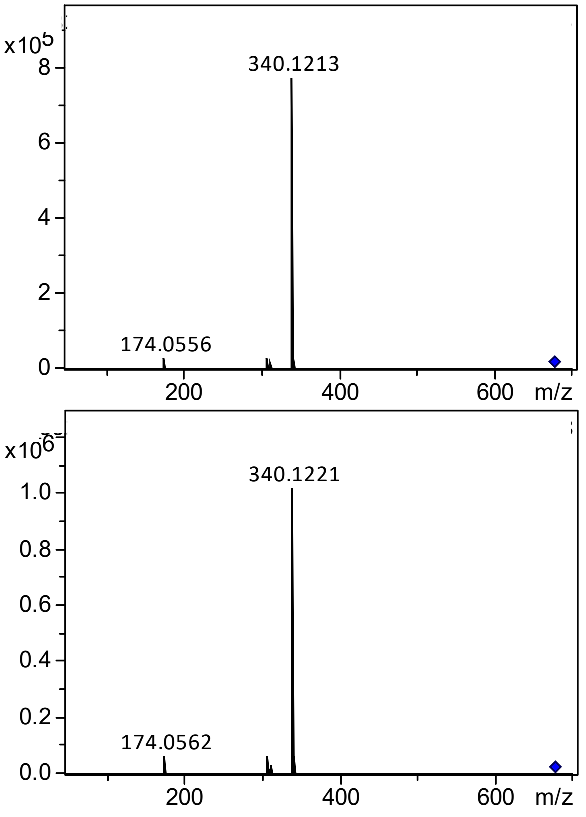

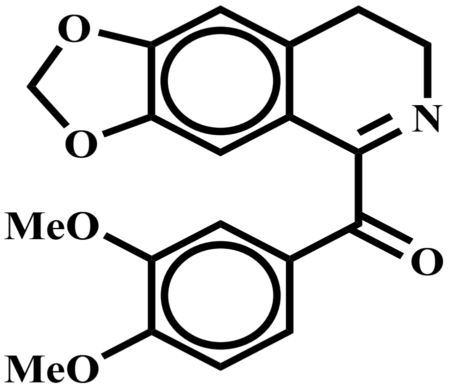

NPRL 833

[M+H]+ : 501.09

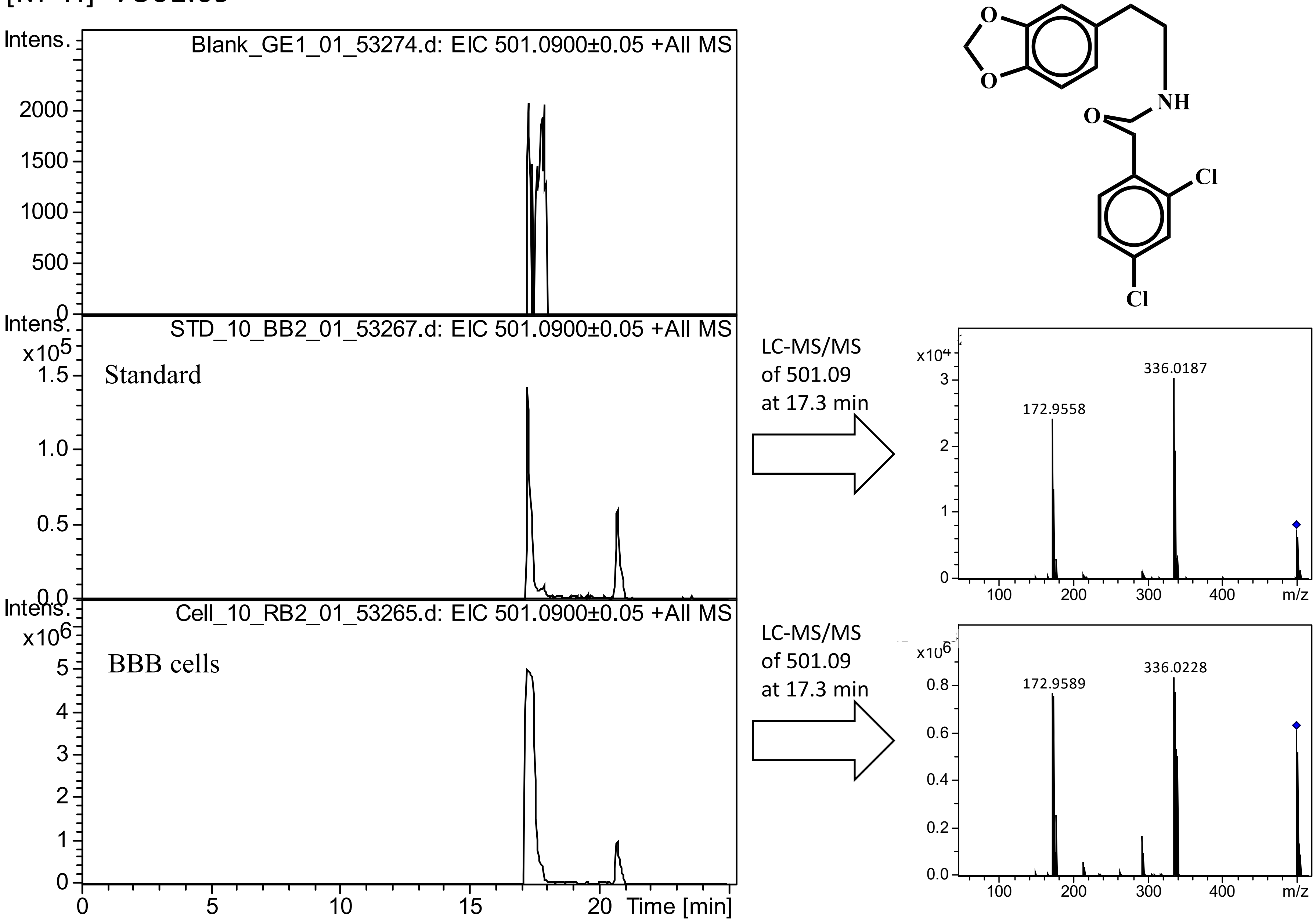

NPRL 835
[M+H]+ : 336.05

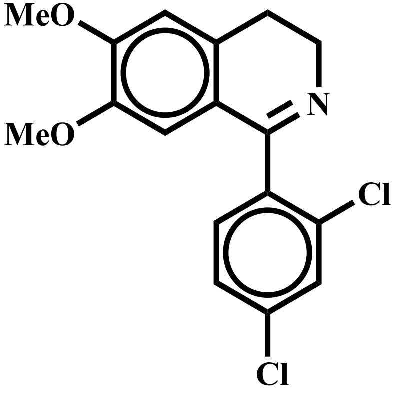

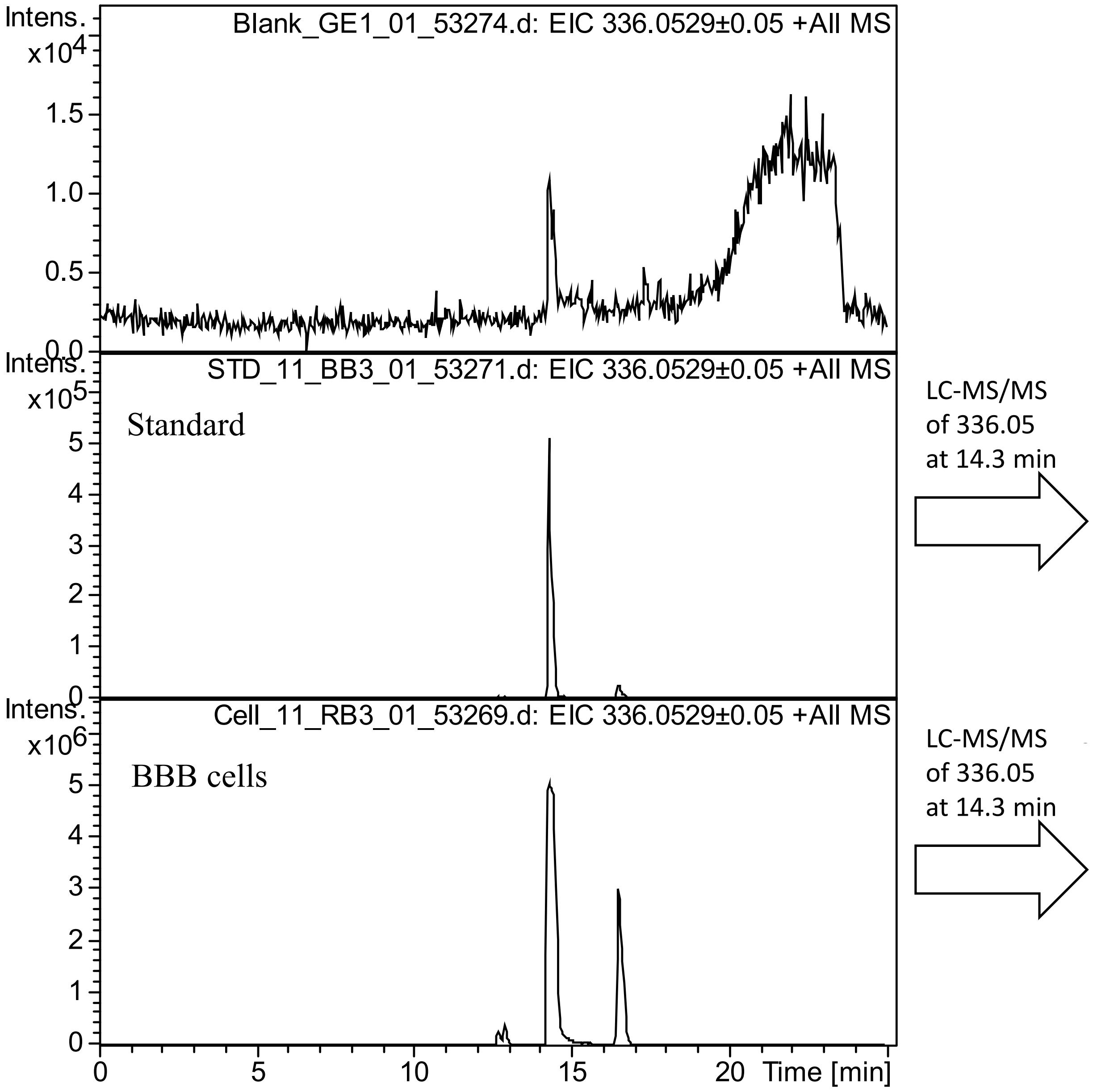

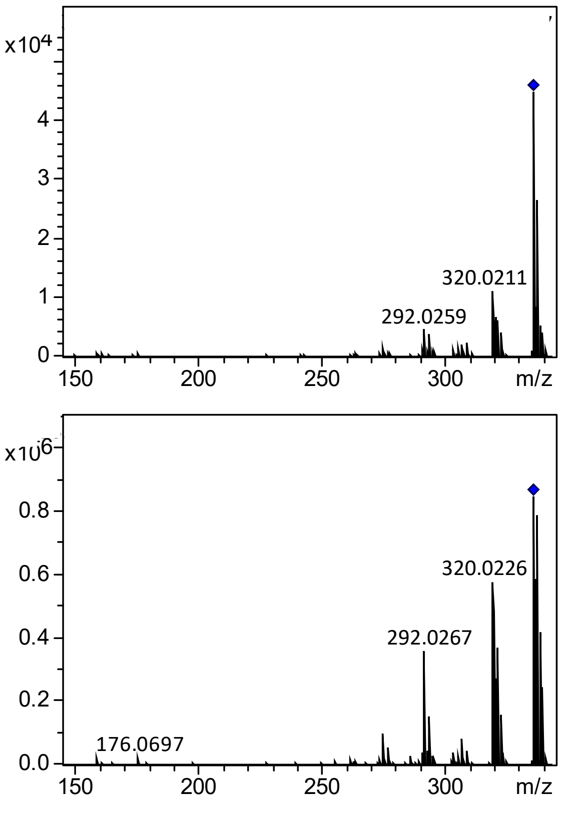

NPRL 836

[M+H]$^+$: 334.03

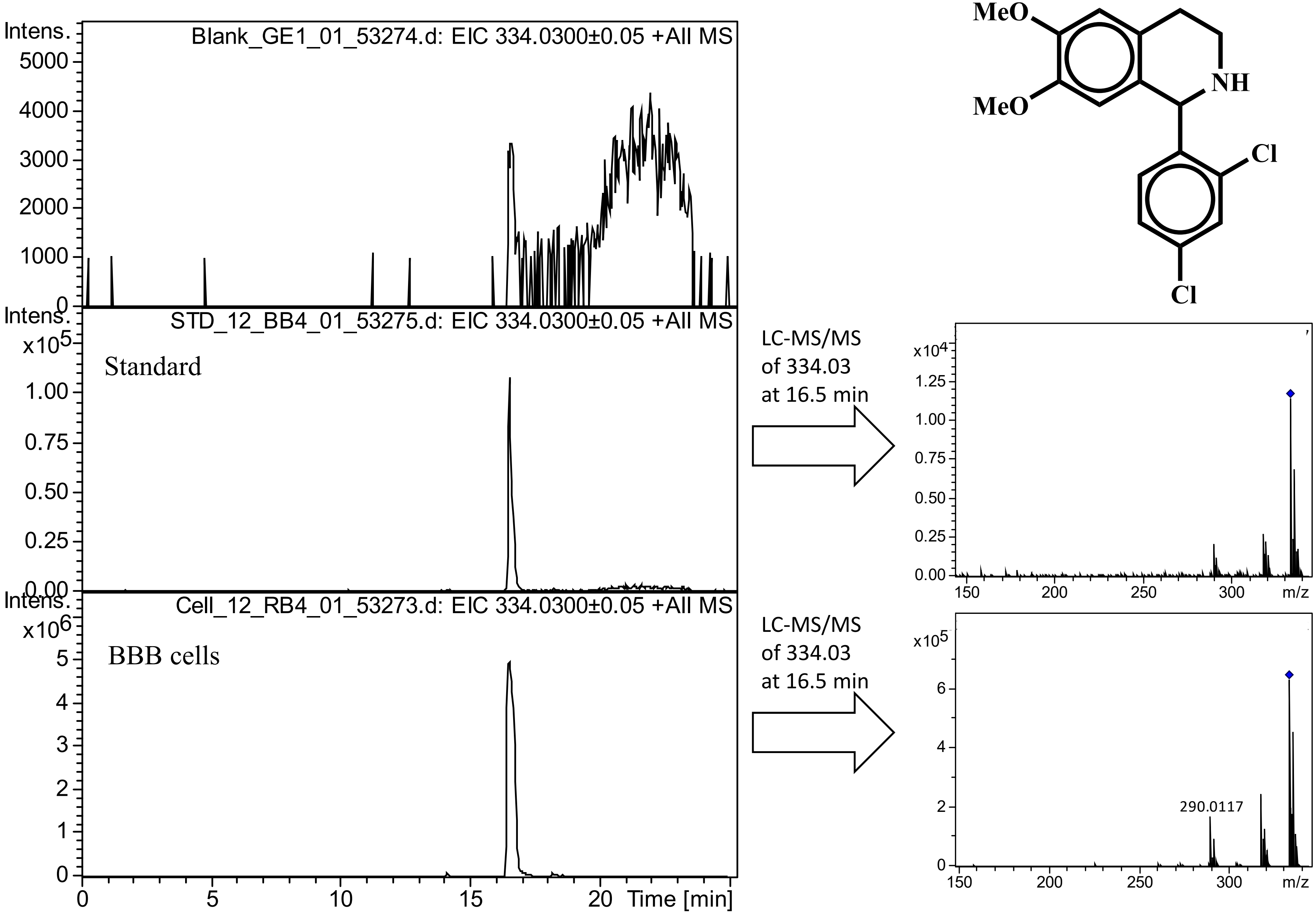

NPRL 842

[M+H]$^+$: 229.05

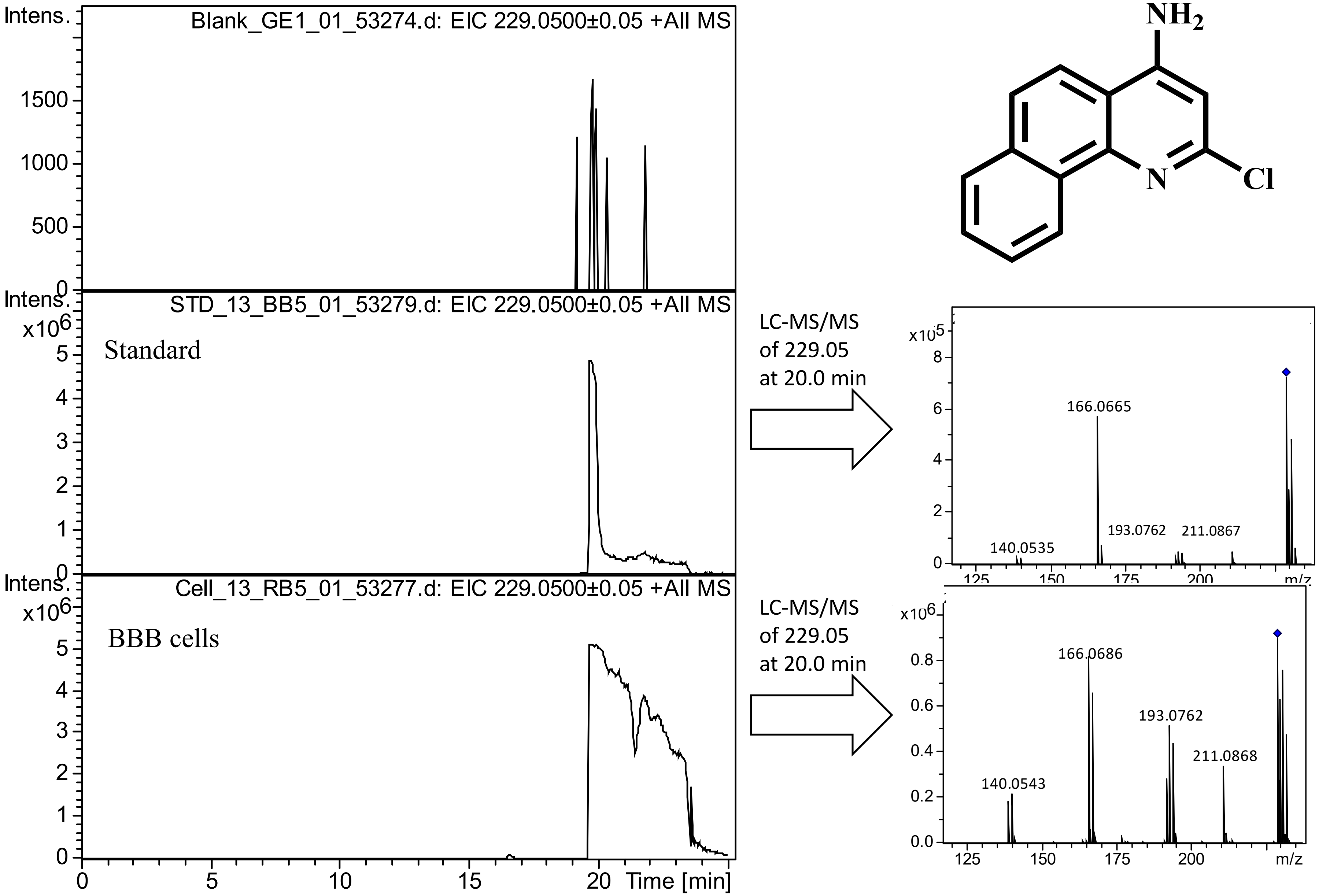

NPRL 1089

$[M+H]^+$: 284.12

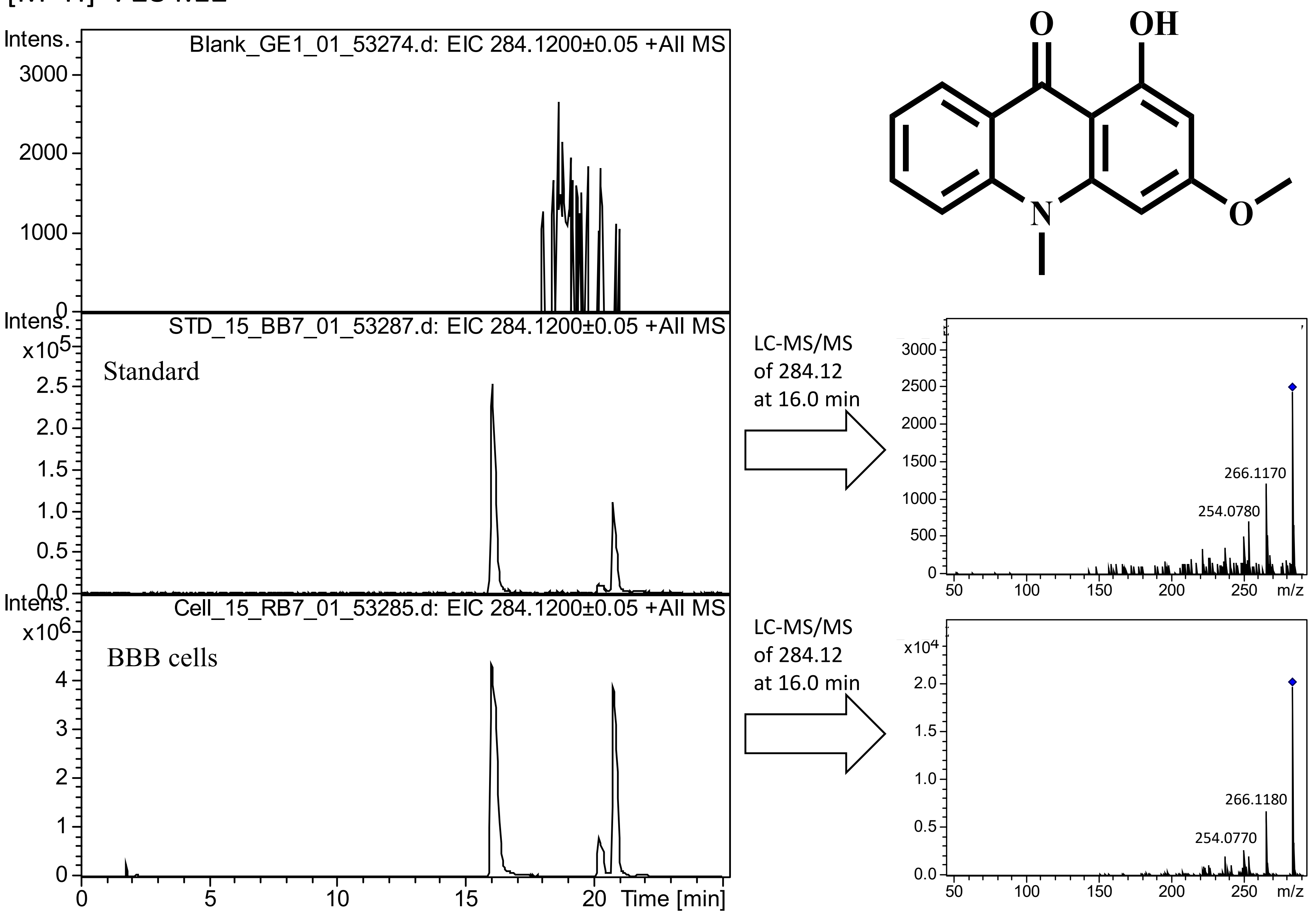

NPRL 1185

$[M+H]^+$: 503.12

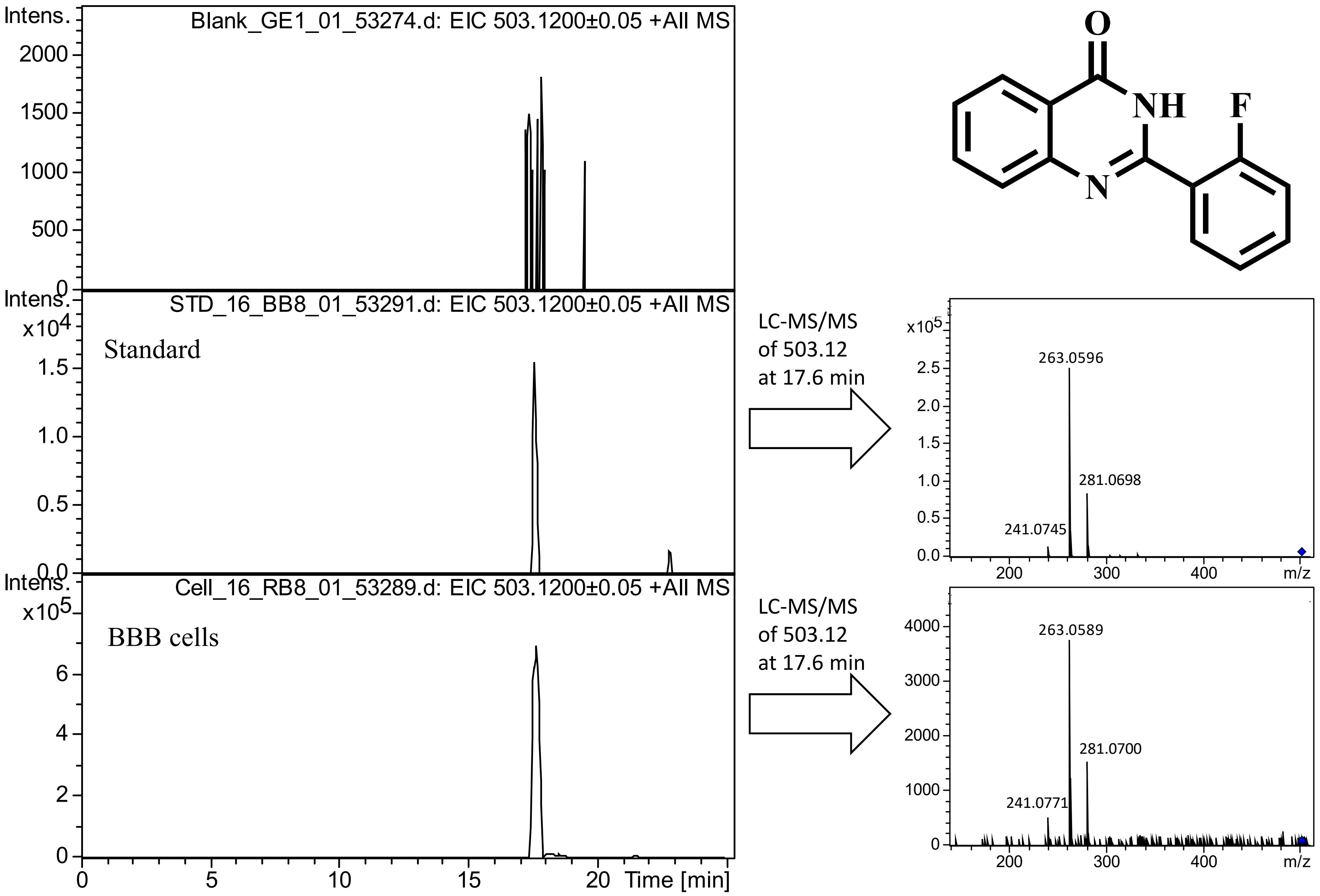

NPRL 1188

$[M+H]^{+}$: 301.09

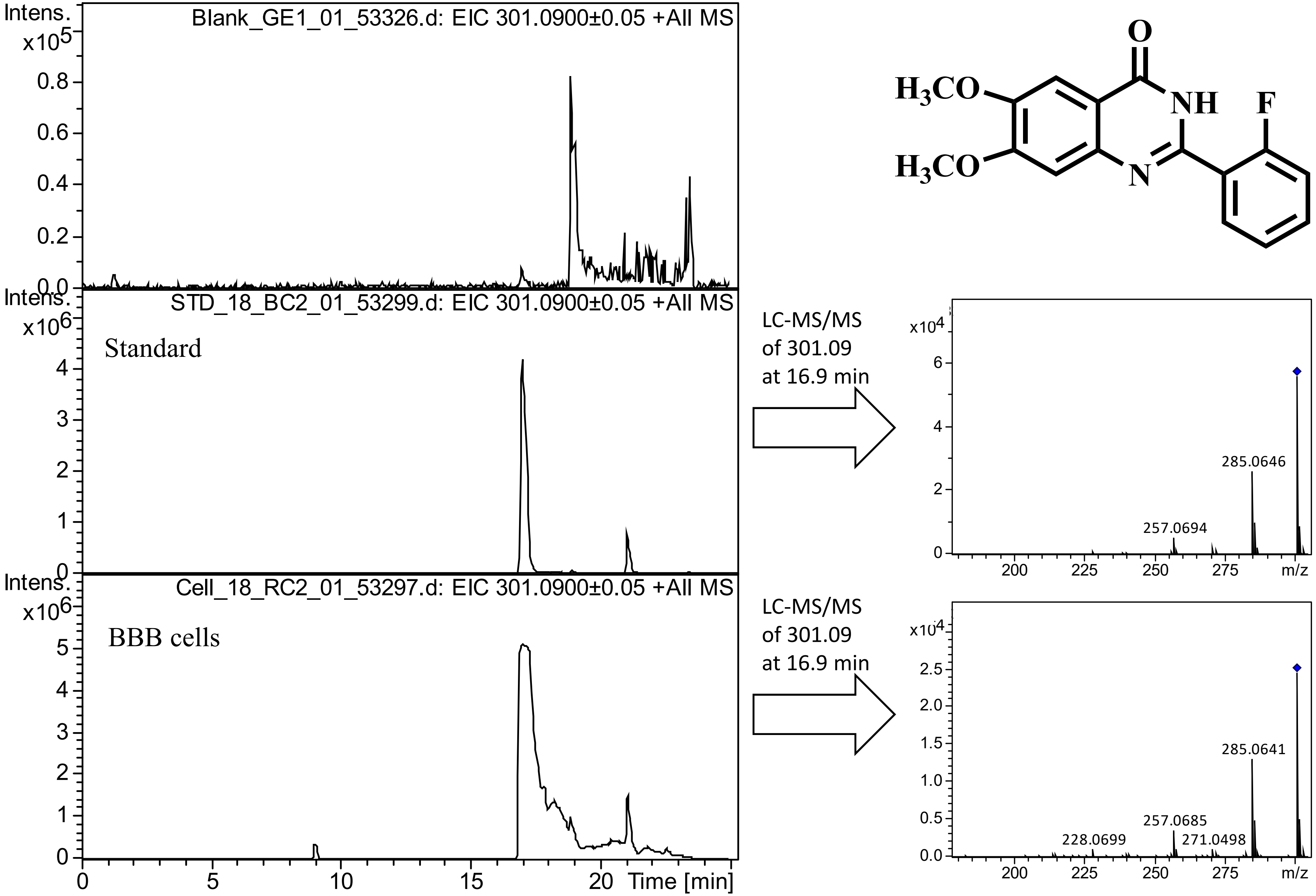

NPRL 1192

[M+H]$^+$: 332.03

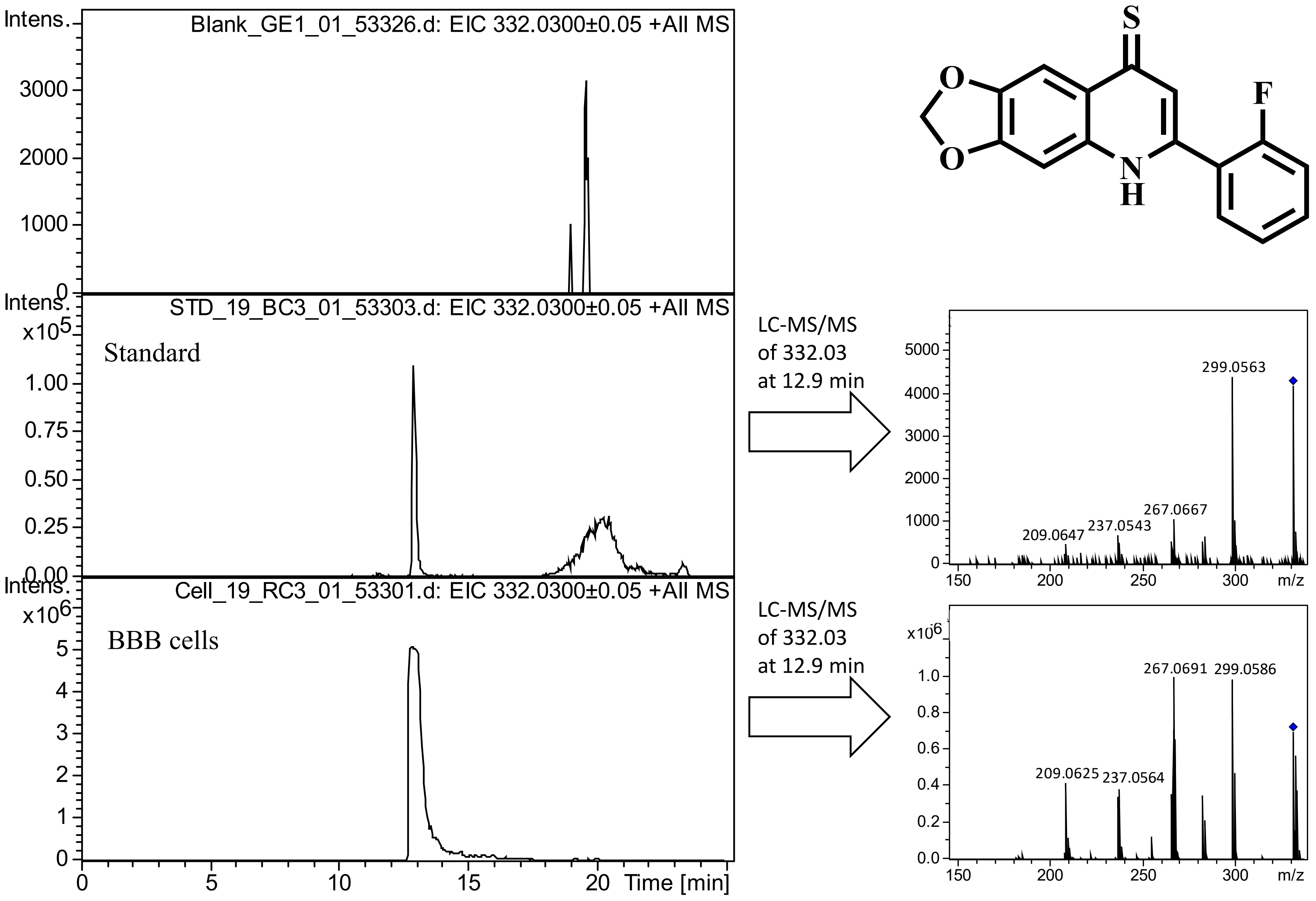

NPRL 1195

$[M+H]^+$: 254.11

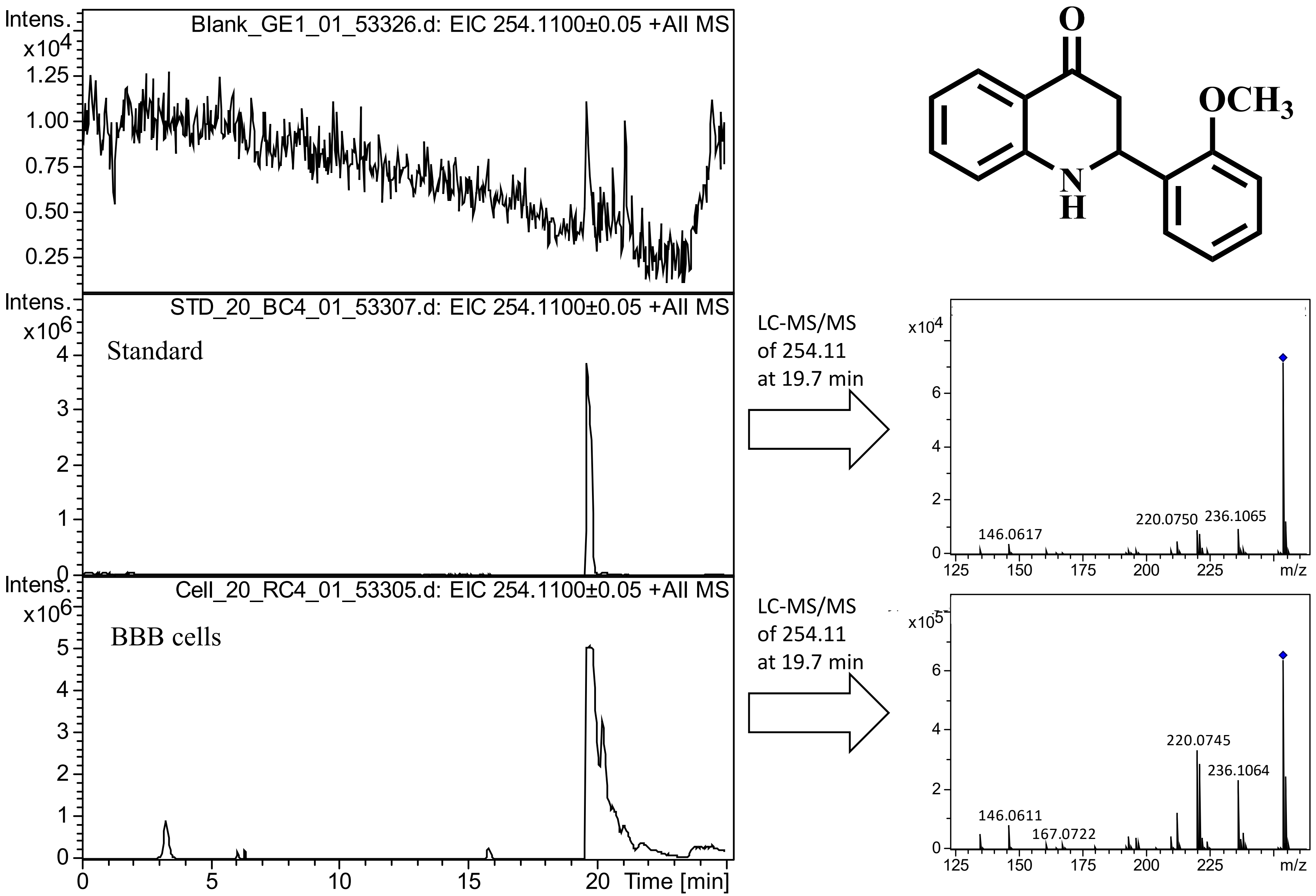

NPRL 1241

[M+H]+ : 284.07

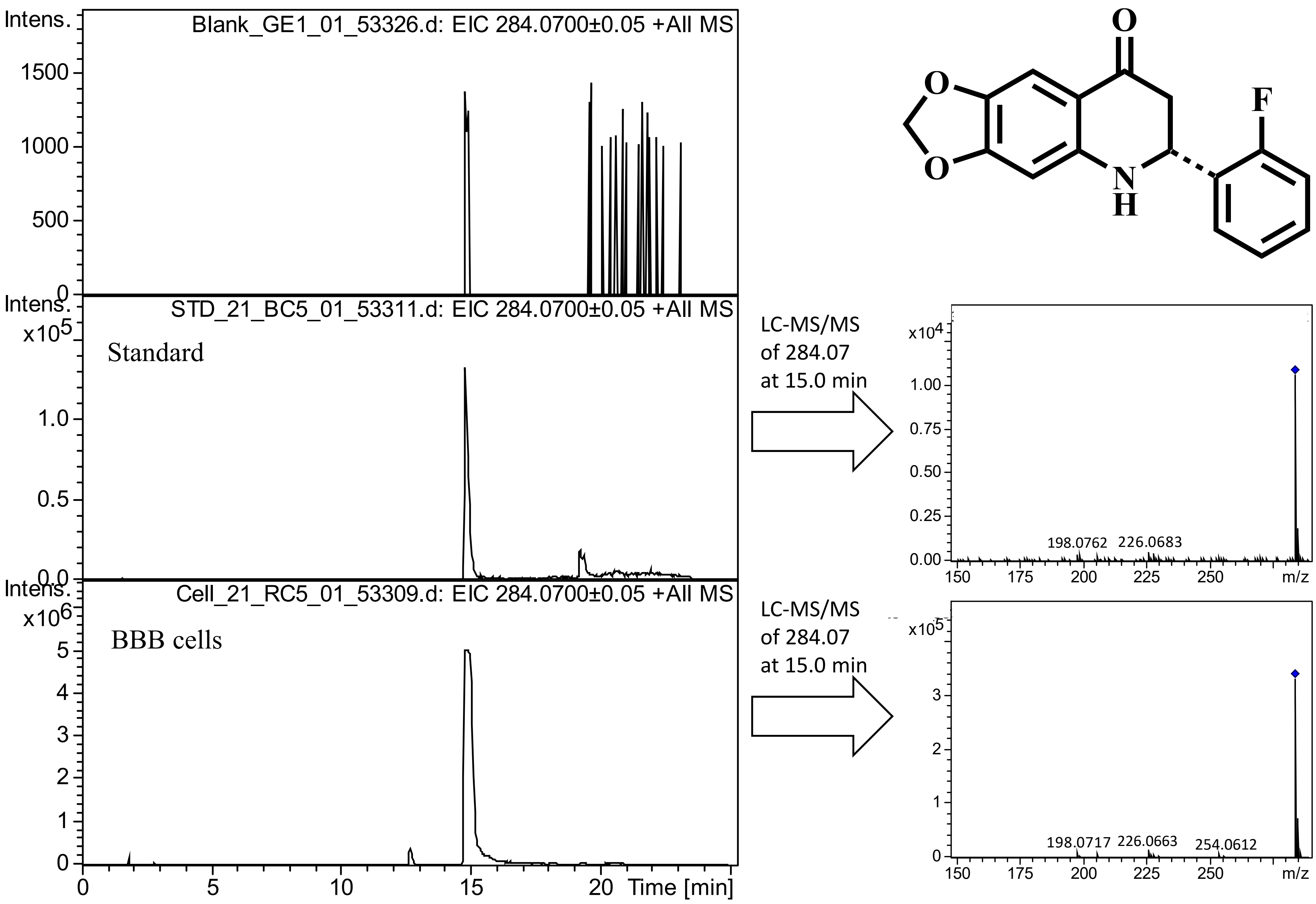

NPRL 1958

$[M+H]^+$: 322.13

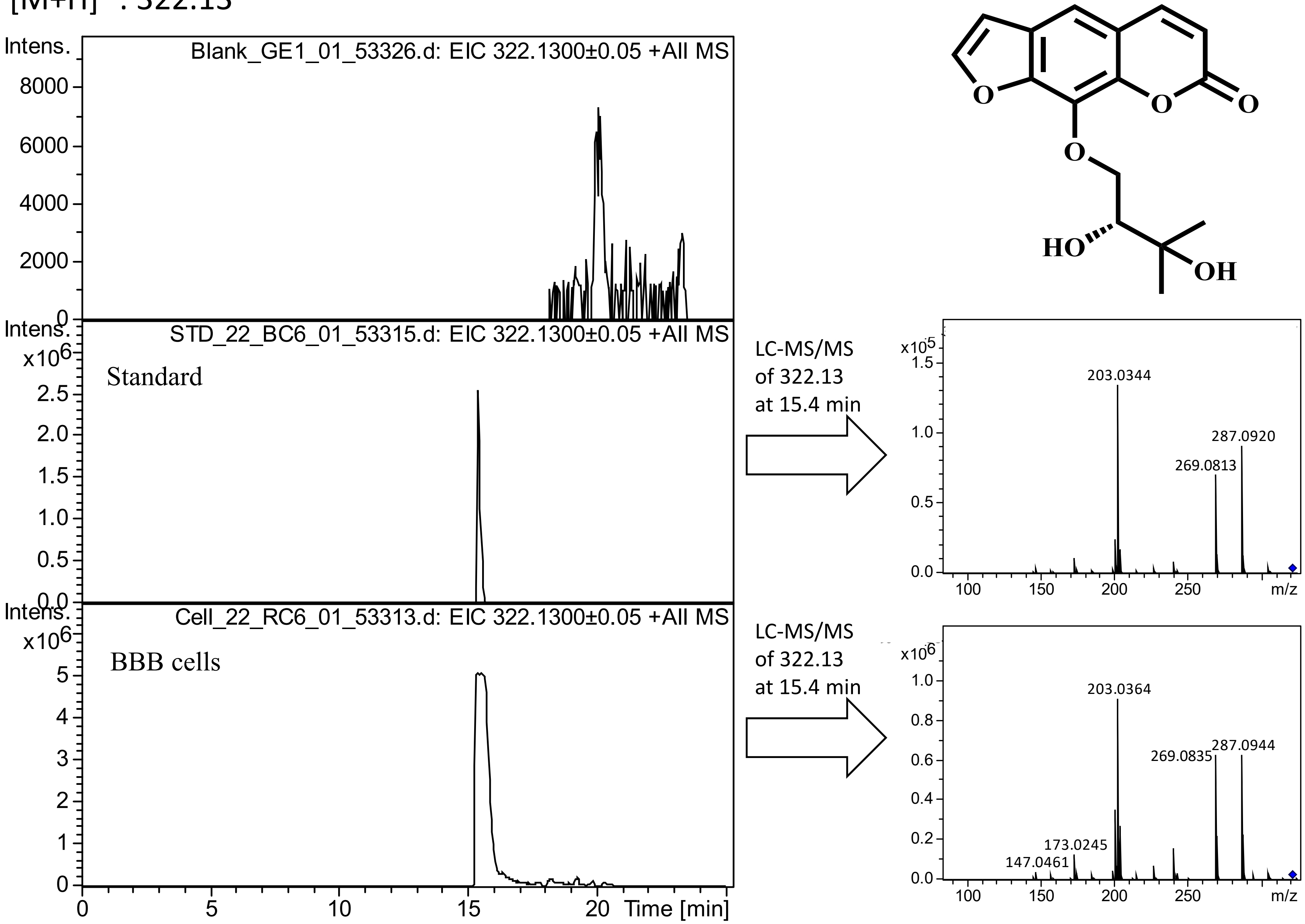

NPRL 2026

$[M+H]^+$: 215.10

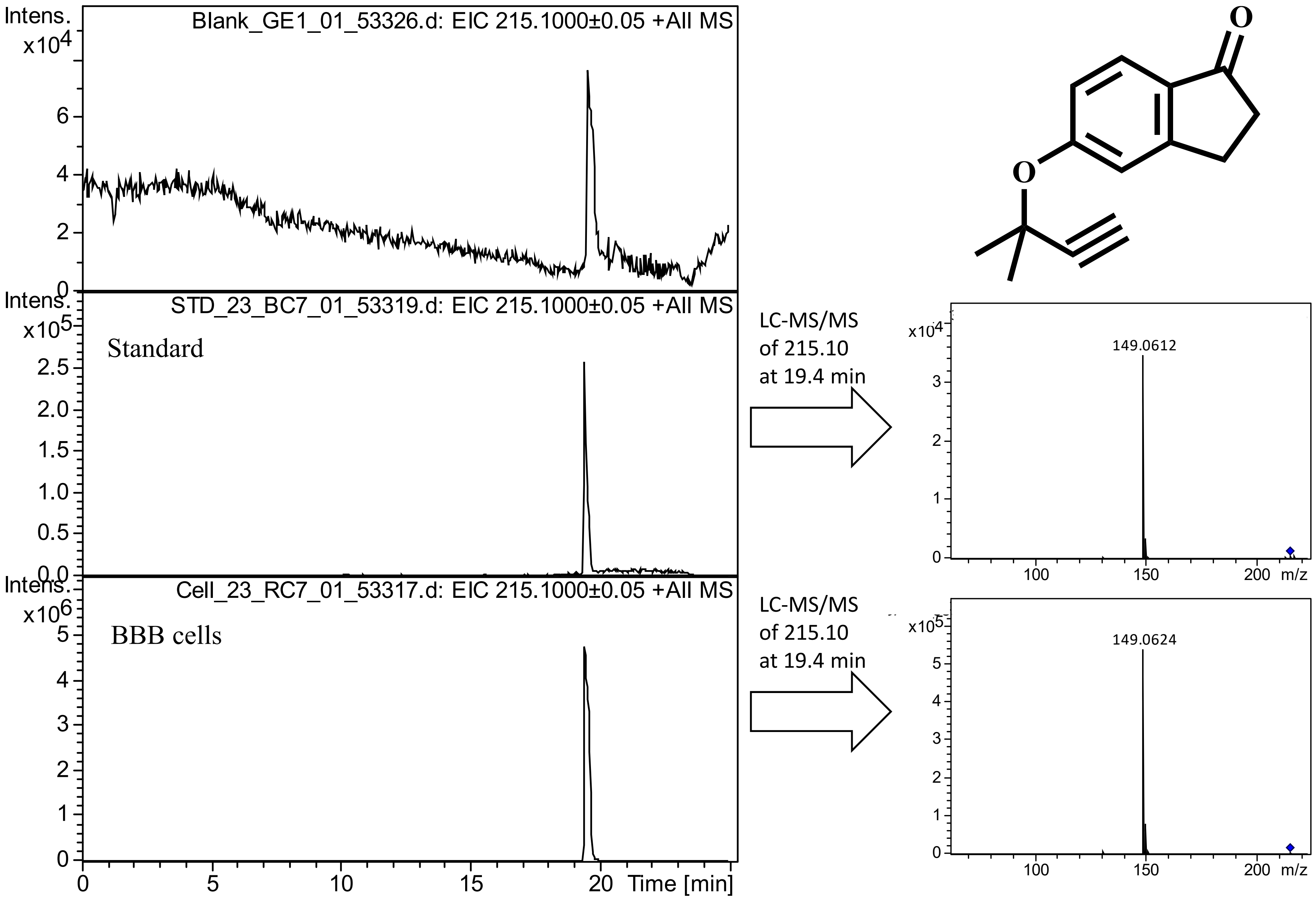

NPRL 2029

$[M+H]^+$: 215.10

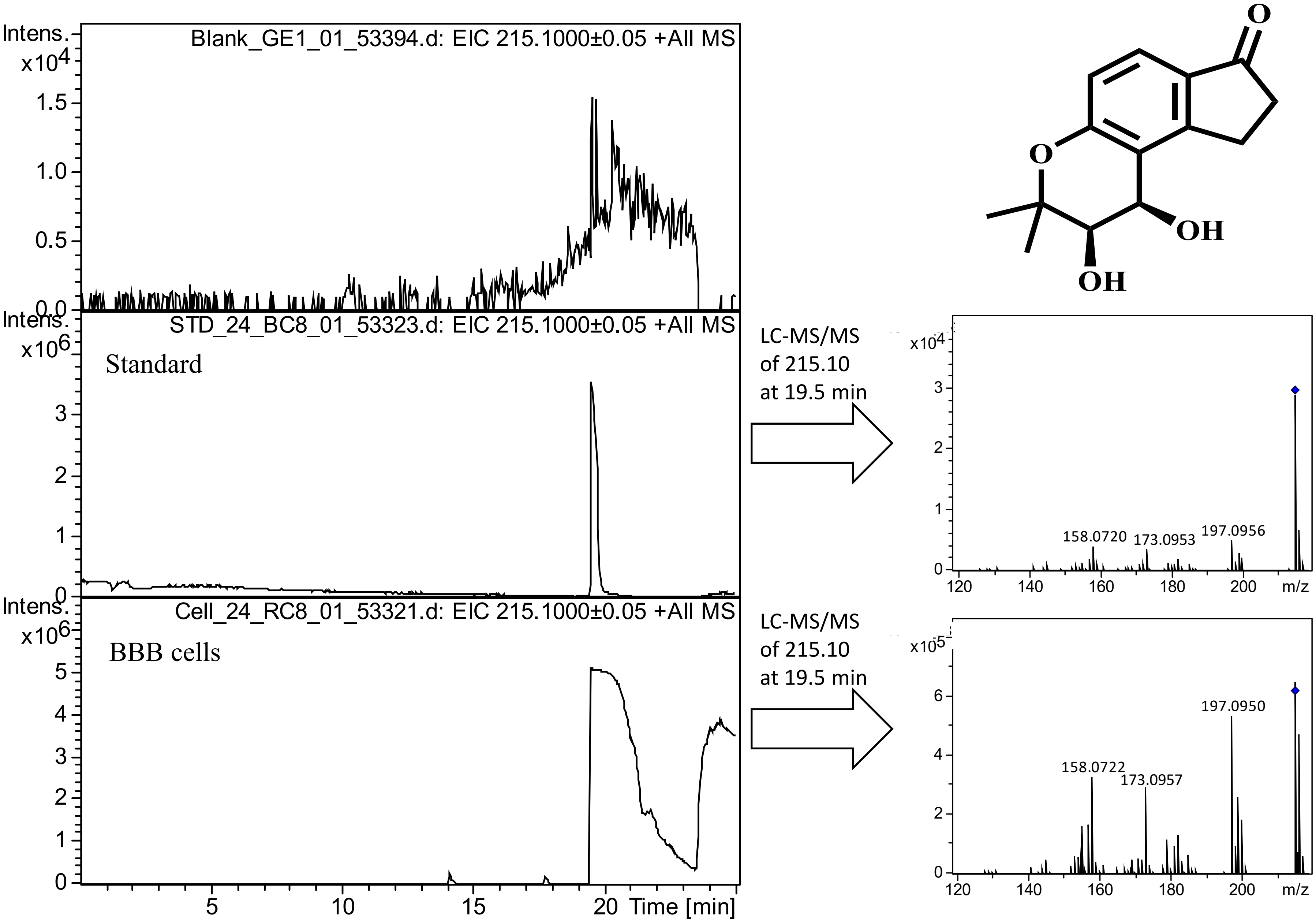

NPRL 2051

$[M+H]^+$: 249.11

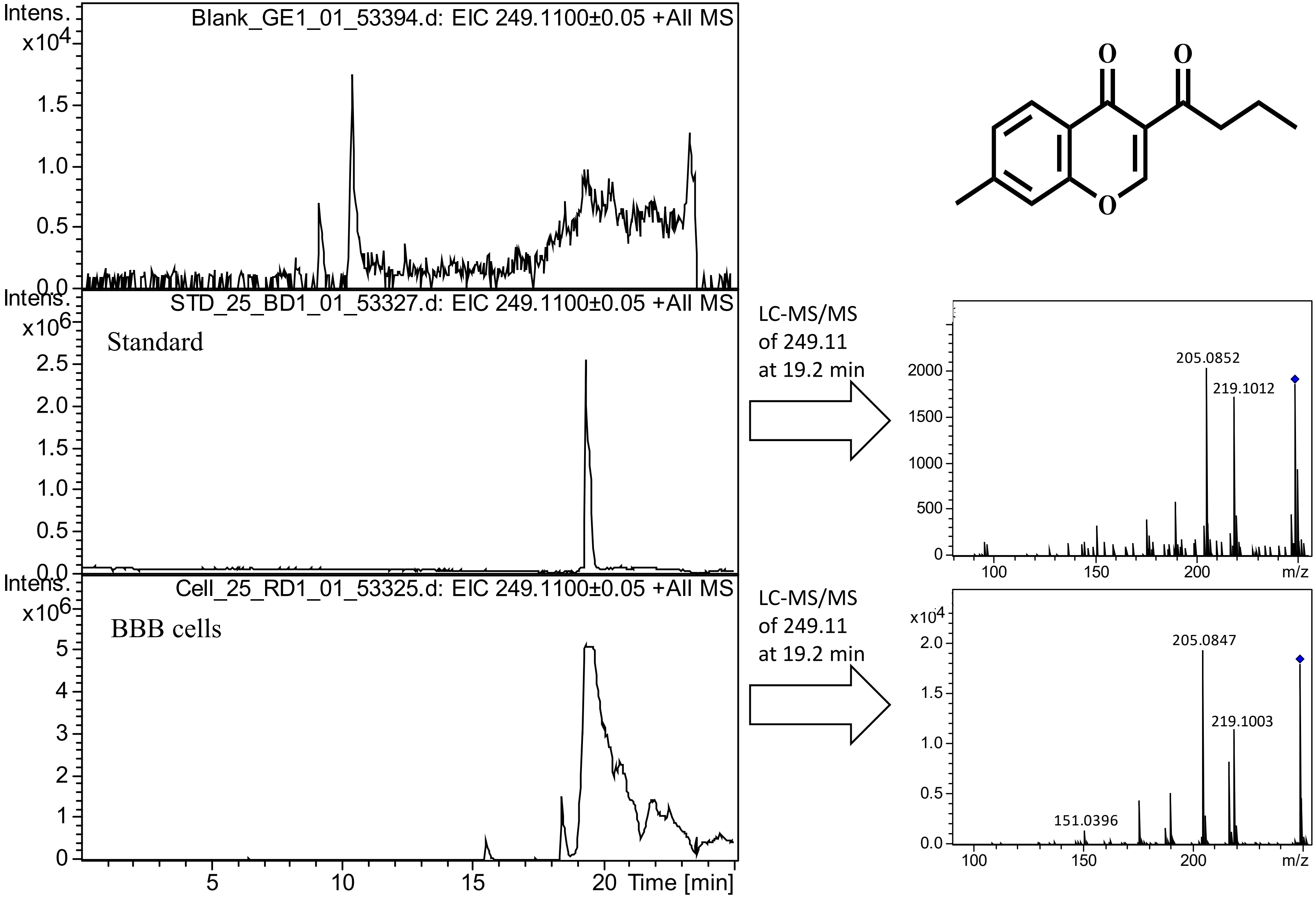

NPRL 2059

$[M+H]^+$: 305.13

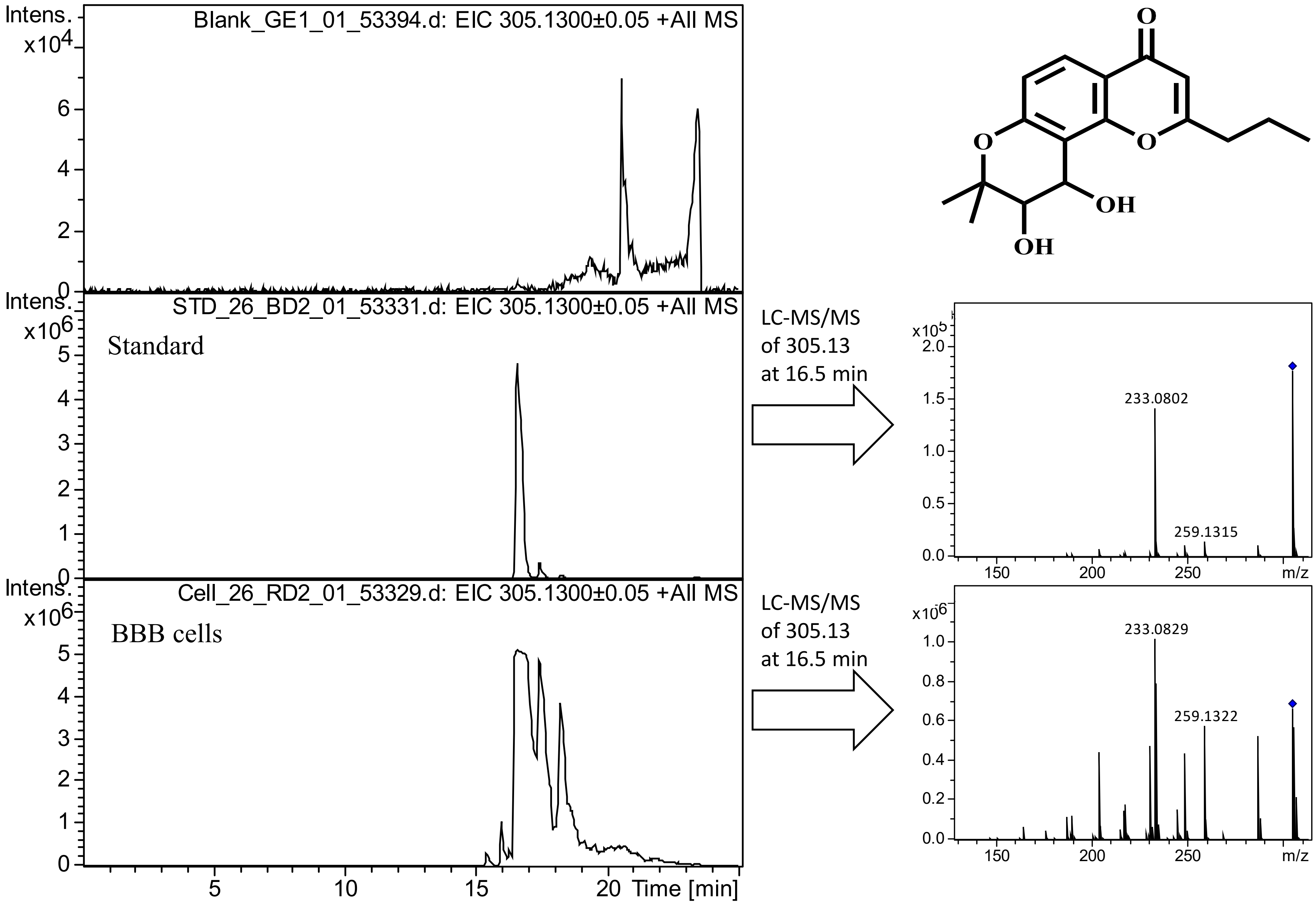

NPRL 2148

$[M+H]^+$: 301.14

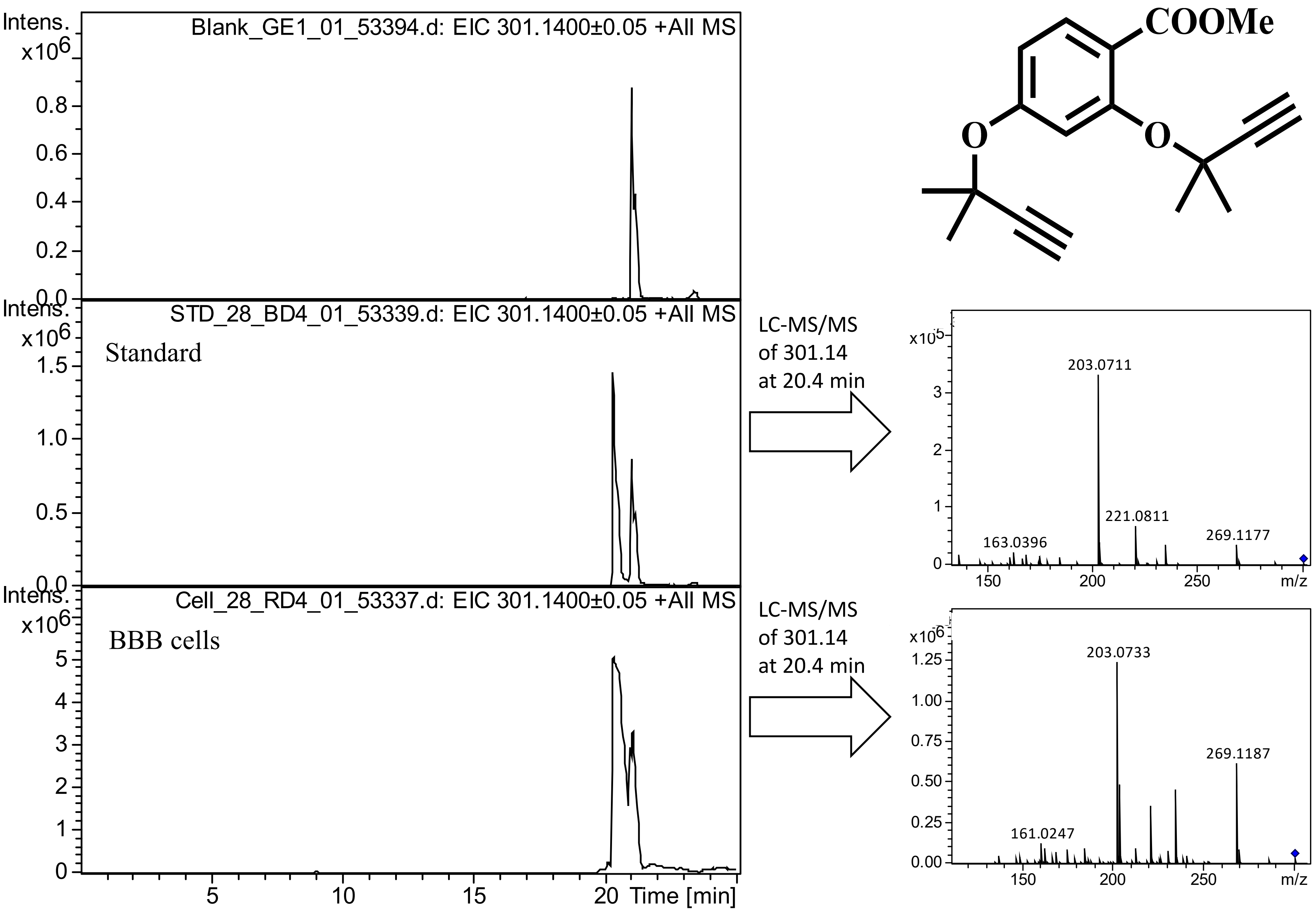

NPRL 3767

$[M+H]^+$: 271.06

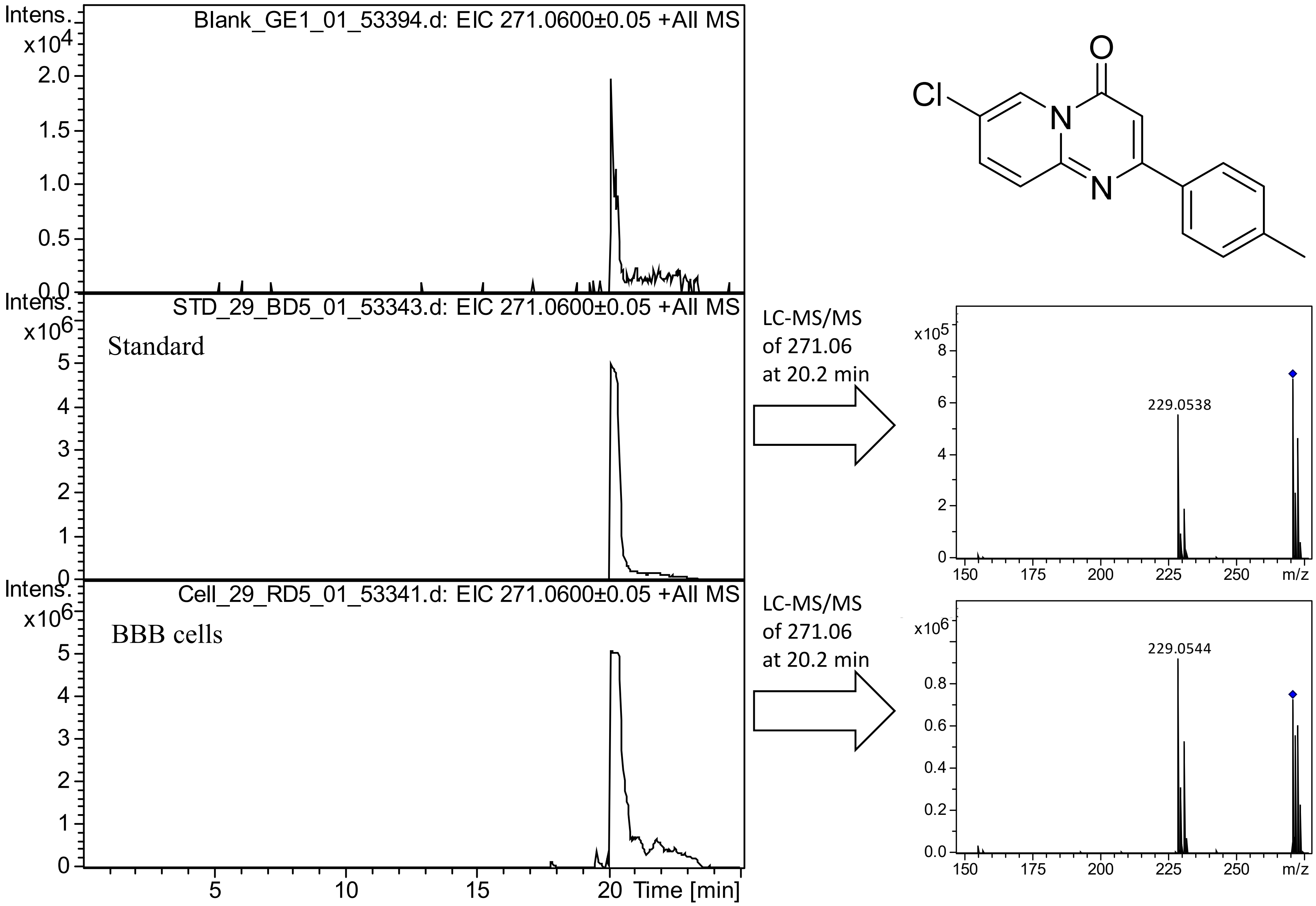

NPRL 2359

$[M+H]^+$: 529.28

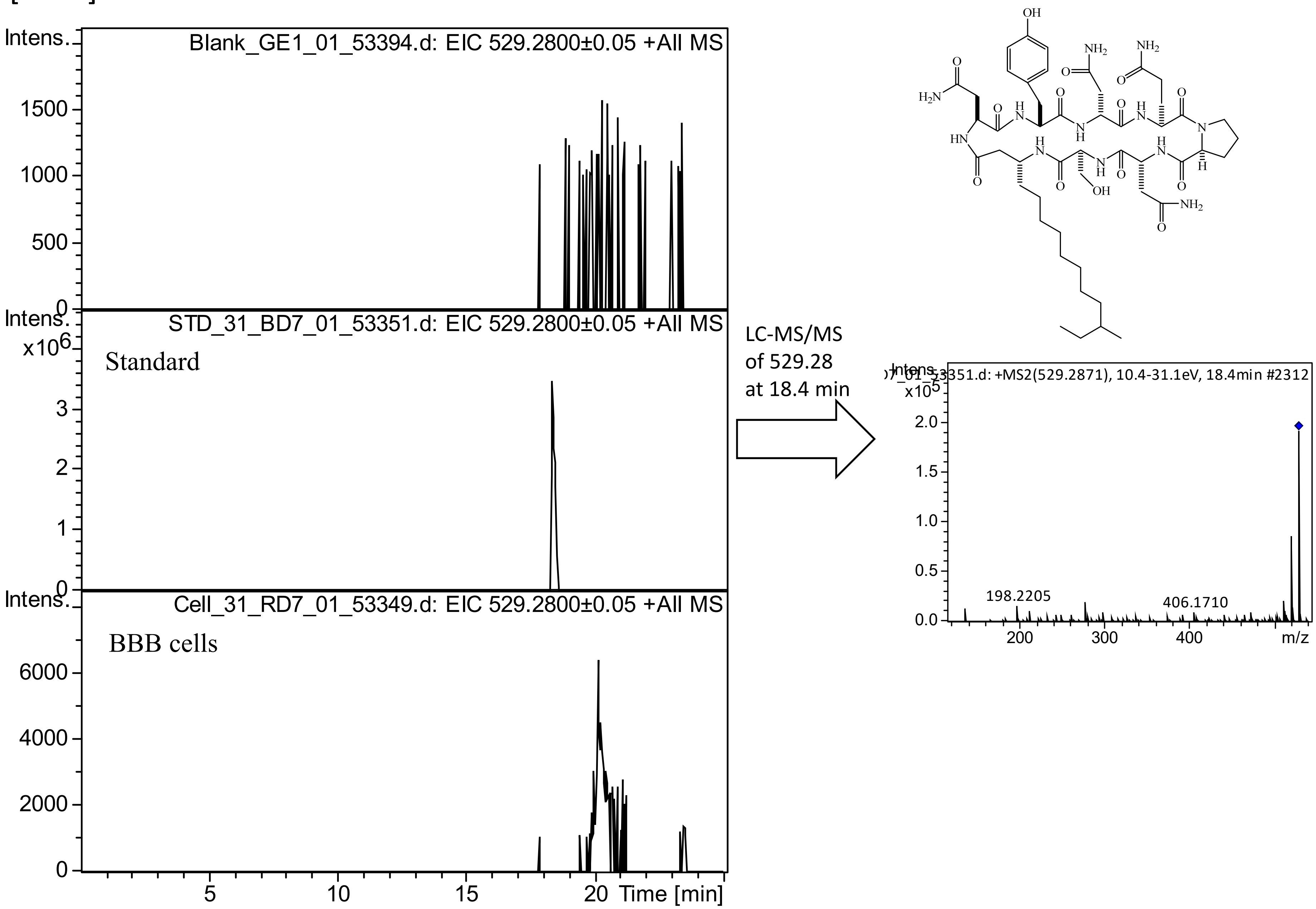

NPRL 2576

$[M+H]^{+}$: 796.48

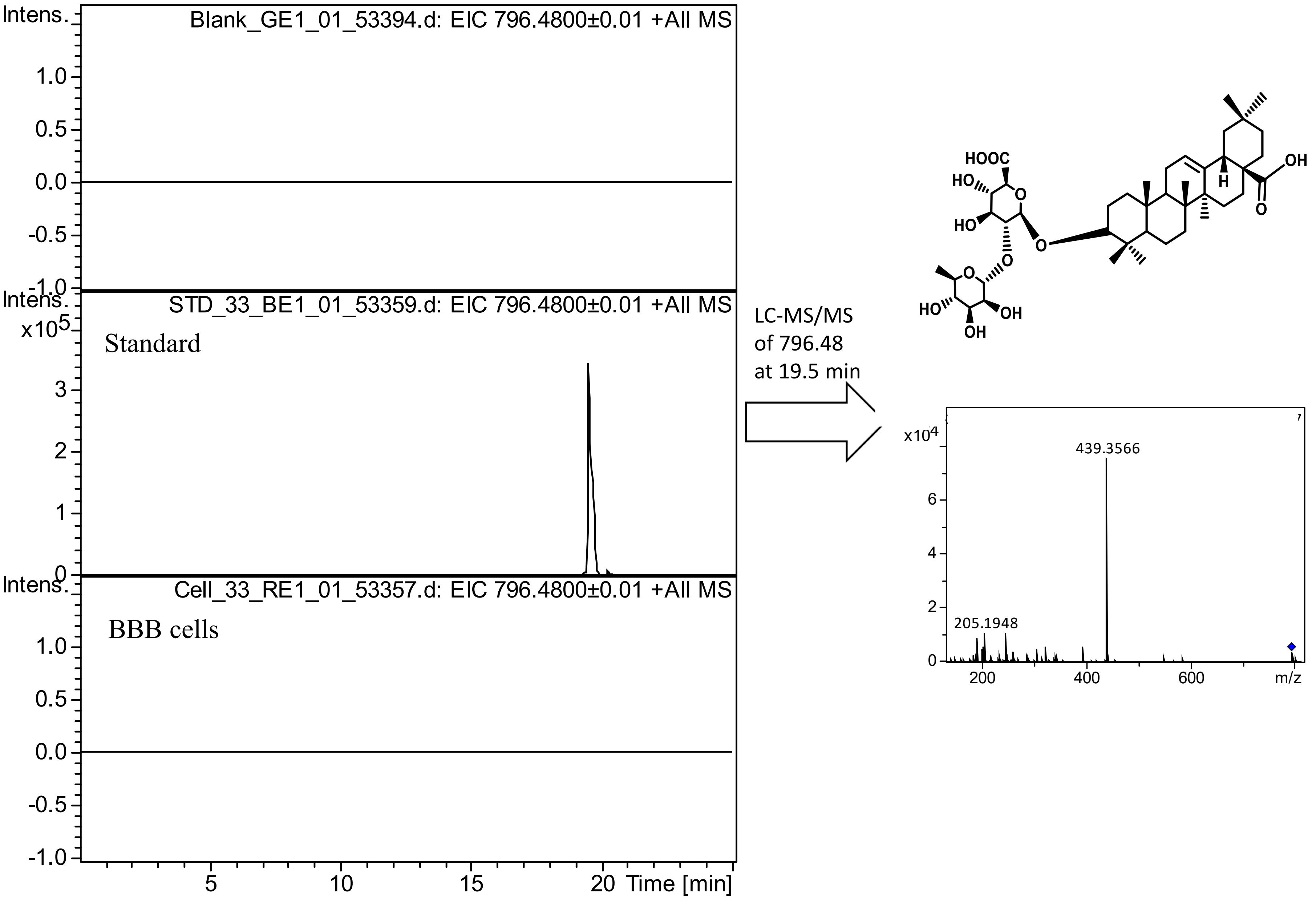

NPRL 2646
[M+H]+ : 741.22

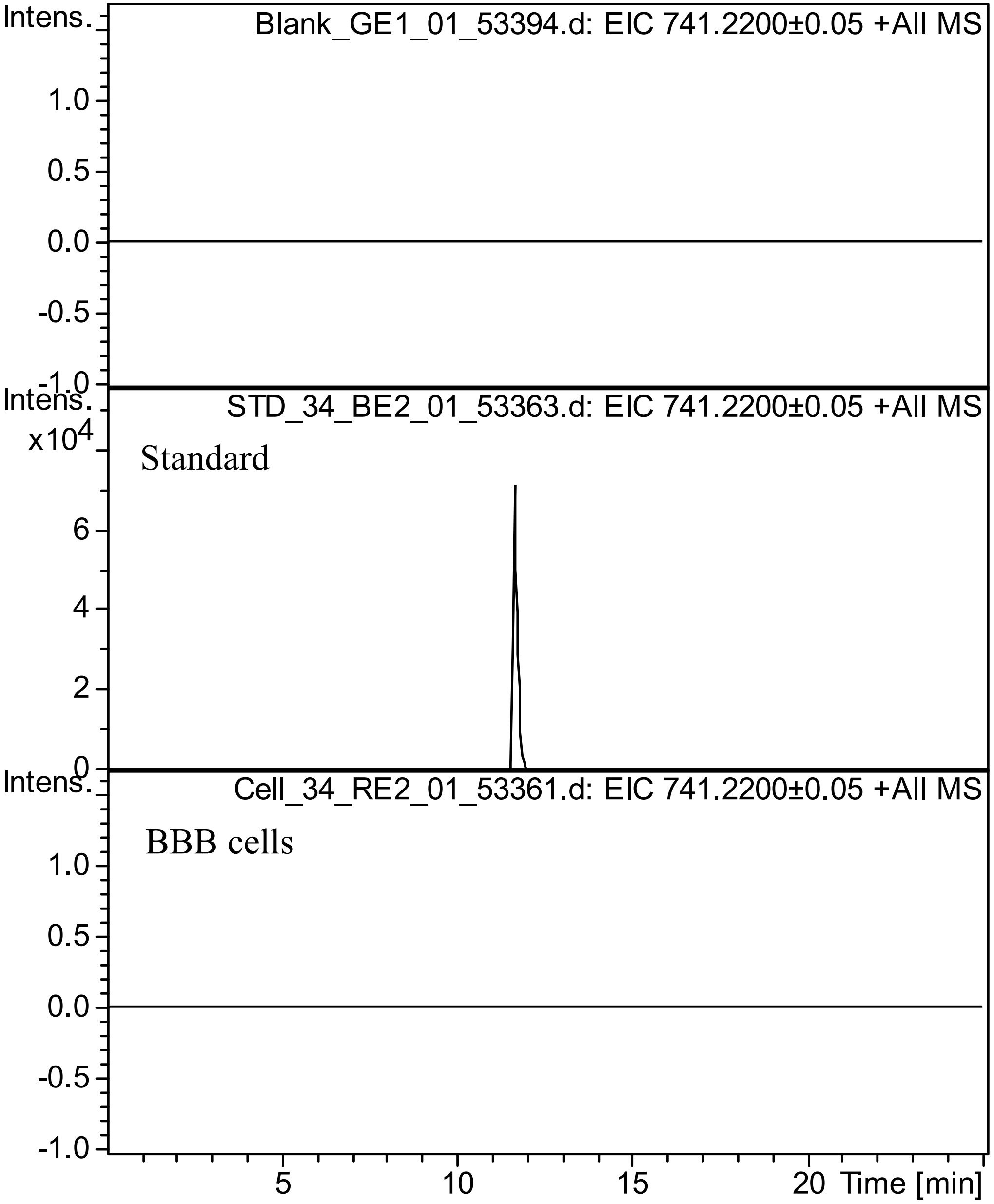

LC-MS/MS
of 741.22
at 11.7 min

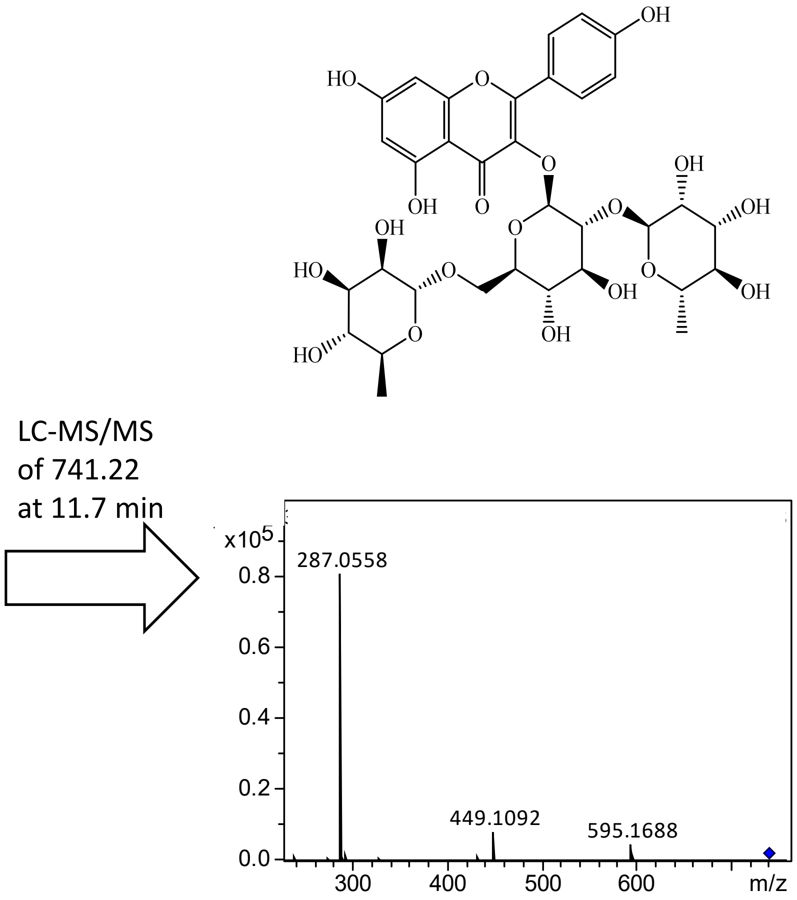

NPRL 3098

$[M+H]^+$: 765.47

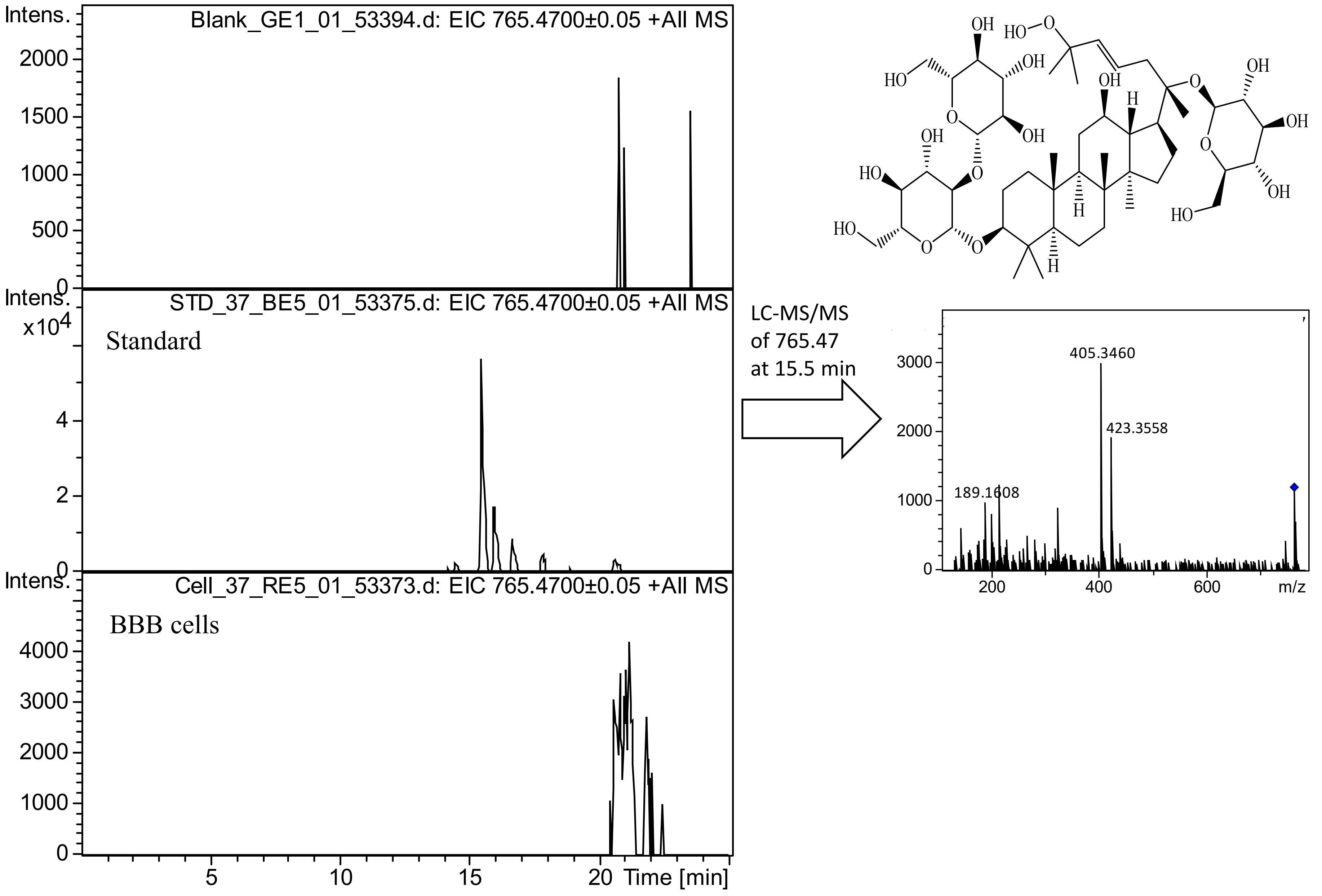

NPRL 3183

$[M+H]^+$: 493.09

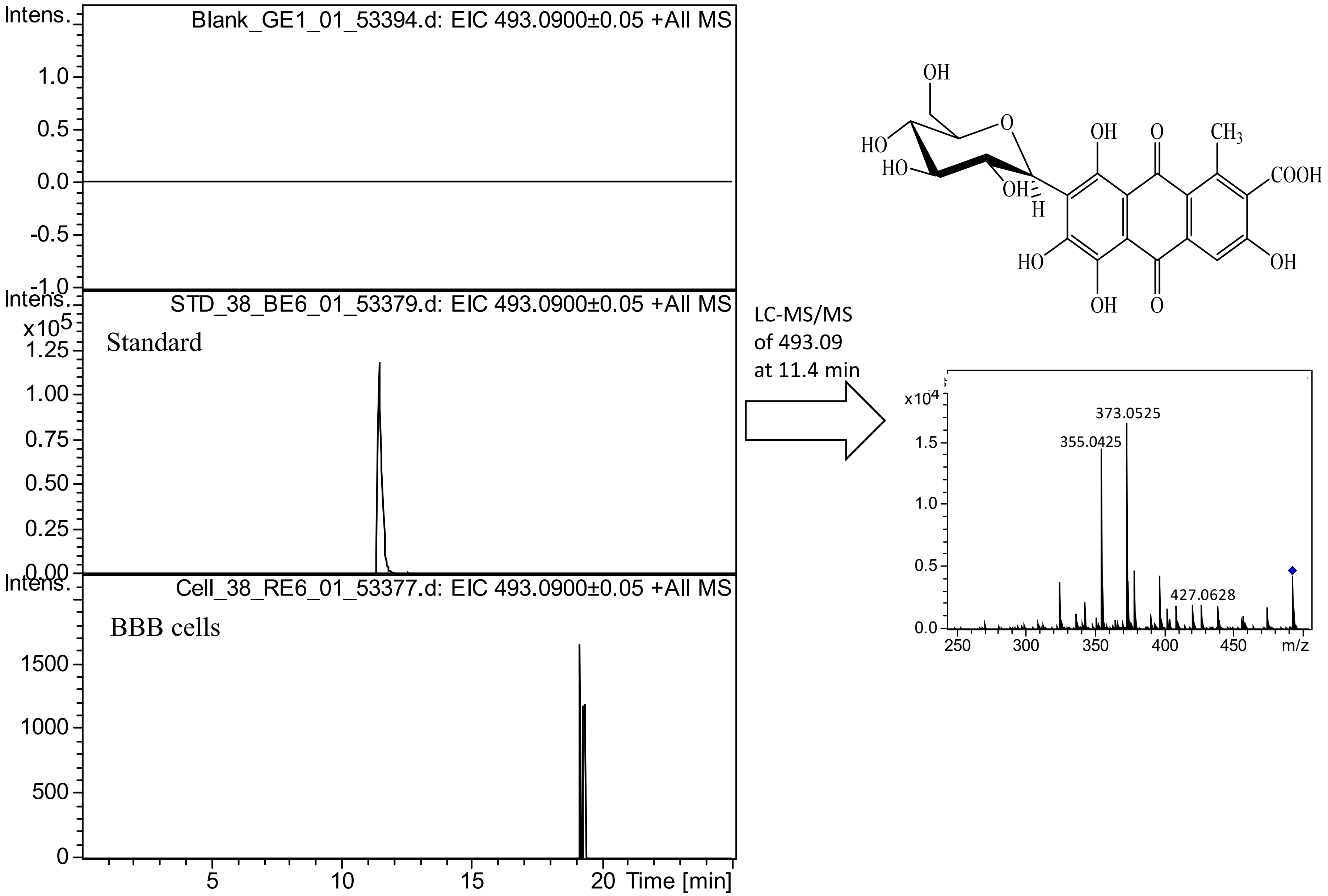

Supplementary Table S1: Training with CMUH-NPRL dataset.

Training sample	Parameter	MegaMolBART-XSMALL	MegaMolBART- SMALL	MegaMolBART- LARGE
CMUH-NPRL test set	Accuracy	0.99 (Incorrect: 6)	0.99 (Incorrect: 4)	0.99 (Incorrect: 3)
	AUC	0.89	0.91	0.92
	F1 score	0.99	0.99	0.99
B3DB dataset	Accuracy	0.61	0.57	0.60
	AUC	0.57	0.56	0.58
	F1 score	0.59	0.57	0.54
Accuracy: TP/Total; AUC: TPR=TP/(TP+FN); FPR=FP/(FP+TN); F1 score: 2/(1/Precision+1/Recall); Precision: TP/(TP+FP); Recall: TP/(TP+FN)				

Supplementary Table S2: Training with B3DB Dataset.

Training sample	B3DB Test Set	MegaMolBART-XSMALL	MegaMolBART-SMALL	MegaMolBART-LARGE
B3DB test set	Accuracy	0.62	0.62	0.63
	AUC	0.63	0.62	0.64
	F1 score	0.62	0.62	0.62
CMUH-NPRL dataset	Accuracy	0.11	0.35	0.35
	AUC	0.58	0.62	0.62
	F1 score	0.11	0.47	0.47
Accuracy: TP/Total; AUC: TPR=TP/(TP+FN), FPR=FP/(FP+TN); F1 score: 2/(1/Precision+1/Recall); Precision: TP/(TP+FP); Recall: TP/(TP+FN)				

Supplementary Table S3: LightBBB v.s. our model on the LightBBB dataset.

	AUC	Specificity	Sensitivity	Accuracy
LightBBB	0.94	0.77	0.99	89%
Our Model	0.93	0.65	0.97	90%

Supplementary Table S4: DeePred v.s. our model on the DeePred dataset.

	AUC	F1-score
DeePred	0.99	0.99
Our Model	0.96	0.95

Supplementary Table S5: Raw data of MegaMolBART analysis on blood brain barrier (BBB) permeability of NPRL compounds.		
**NPRL ID**	**PRED_SCORES**	**PRED_LABEL**
NPRL 1	0.45393	BBB-
NPRL 10	0.26538	BBB-
NPRL 104	0.90741	BBB+
NPRL 105	0.94339	BBB+
NPRL 106	0.89884	BBB+
NPRL 107	0.39566	BBB-
NPRL 108	0.64440	BBB+
NPRL 109	0.88389	BBB+
NPRL 11	0.63237	BBB+
NPRL 111	0.25134	BBB-
NPRL 112	0.29219	BBB-
NPRL 113	0.72508	BBB+
NPRL 114	0.90522	BBB+
NPRL 115	0.00139	BBB-
NPRL 116	0.30958	BBB-
NPRL 117	0.20910	BBB-
NPRL 118	0.02755	BBB-
NPRL 119	0.06277	BBB-
NPRL 12	0.40601	BBB-
NPRL 120	0.19995	BBB-
NPRL 121	0.00617	BBB-
NPRL 122	0.70562	BBB+
NPRL 124	0.01115	BBB-
NPRL 125	0.00152	BBB-
NPRL 126	0.00155	BBB-
NPRL 127	0.00528	BBB-
NPRL 128	0.00148	BBB-
NPRL 129	0.80271	BBB+
NPRL 13	0.01433	BBB-
NPRL 130	0.47620	BBB-
NPRL 131	0.79631	BBB+
NPRL 133	0.89034	BBB+
NPRL 134	0.40152	BBB-
NPRL 135	0.97464	BBB+
NPRL 136	0.94924	BBB+
NPRL 137	0.95735	BBB+
NPRL 139	0.84235	BBB+
NPRL 14	0.00227	BBB-
NPRL 140	0.99775	BBB+
NPRL 141	0.37611	BBB-
NPRL 142	0.99170	BBB+
NPRL 145	0.93260	BBB+
NPRL 146	0.94191	BBB+
NPRL 149	0.98268	BBB+
NPRL 15	0.00250	BBB-
NPRL 150	0.31763	BBB-
NPRL 151	0.99560	BBB+
NPRL 152	0.95973	BBB+
NPRL 153	0.99286	BBB+
NPRL 154	0.96706	BBB+
NPRL 155	0.99093	BBB+
NPRL 156	0.43547	BBB-
NPRL 157	0.28566	BBB-
NPRL 158	0.99878	BBB+
NPRL 159	0.99036	BBB+

NPRL 16	0.82473	BBB+
NPRL 164	0.99415	BBB+
NPRL 165	0.39210	BBB-
NPRL 166	0.97431	BBB+
NPRL 168	0.02276	BBB-
NPRL 169	0.98003	BBB+
NPRL 17	0.99567	BBB+
NPRL 170	0.98367	BBB+
NPRL 171	0.65060	BBB+
NPRL 172	0.99605	BBB+
NPRL 173	0.96740	BBB+
NPRL 174	0.89727	BBB+
NPRL 175	0.02776	BBB-
NPRL 177	0.99237	BBB+
NPRL 178	0.53367	BBB+
NPRL 179	0.93865	BBB+
NPRL 18	0.93237	BBB+
NPRL 180	0.80135	BBB+
NPRL 181	0.48458	BBB-
NPRL 1814	0.00105	BBB-
NPRL 1816	0.00040	BBB-
NPRL 1817	0.00032	BBB-
NPRL 1818	0.00041	BBB-
NPRL 1819	0.00030	BBB-
NPRL 182	0.99436	BBB+
NPRL 1820	0.00104	BBB-
NPRL 1821	0.01710	BBB-
NPRL 1822	0.50921	BBB+
NPRL 1823	0.85355	BBB+
NPRL 1825	0.49881	BBB-
NPRL 1826	0.93773	BBB+
NPRL 1827	0.00196	BBB-
NPRL 1828	0.60257	BBB+
NPRL 1829	0.81099	BBB+
NPRL 1830	0.27776	BBB-
NPRL 1831	0.11780	BBB-
NPRL 1833	0.01201	BBB-
NPRL 1834	0.00033	BBB-
NPRL 1835	0.79119	BBB+
NPRL 1836	0.98740	BBB+
NPRL 1837	0.89497	BBB+
NPRL 1838	0.24268	BBB-
NPRL 1839	0.00017	BBB-
NPRL 184	0.49445	BBB-
NPRL 1840	0.34009	BBB-
NPRL 1841	0.00160	BBB-
NPRL 1842	0.26273	BBB-
NPRL 1843	0.55278	BBB+
NPRL 1844	0.03153	BBB-
NPRL 1847	0.47701	BBB-
NPRL 1848	0.47869	BBB-
NPRL 1849	0.95075	BBB+
NPRL 185	0.99394	BBB+
NPRL 1850	0.47368	BBB-
NPRL 1851	0.71548	BBB+
NPRL 1852	0.01299	BBB-
NPRL 1854	0.04629	BBB-

NPRL 1855	0.04217	BBB-
NPRL 1857	0.94250	BBB+
NPRL 1859	0.07863	BBB-
NPRL 186	0.98057	BBB+
NPRL 1860	0.67976	BBB+
NPRL 1862	0.00942	BBB-
NPRL 1863	0.00601	BBB-
NPRL 1864	0.00476	BBB-
NPRL 1866	0.01774	BBB-
NPRL 1867	0.00158	BBB-
NPRL 1868	0.00143	BBB-
NPRL 1869	0.00260	BBB-
NPRL 187	0.04818	BBB-
NPRL 1870	0.00270	BBB-
NPRL 1871	0.01230	BBB-
NPRL 1873	0.97580	BBB+
NPRL 1874	0.96846	BBB+
NPRL 1875	0.37278	BBB-
NPRL 1876	0.81821	BBB+
NPRL 1878	0.99679	BBB+
NPRL 188	0.45436	BBB-
NPRL 1880	0.92575	BBB+
NPRL 1881	0.90539	BBB+
NPRL 1882	0.71176	BBB+
NPRL 1883	0.98567	BBB+
NPRL 1884	0.09728	BBB-
NPRL 1885	0.96612	BBB+
NPRL 1886	0.94736	BBB+
NPRL 1887	0.11249	BBB-
NPRL 1888	0.96522	BBB+
NPRL 1889	0.97478	BBB+
NPRL 189	0.31078	BBB-
NPRL 1890	0.81021	BBB+
NPRL 1891	0.99305	BBB+
NPRL 1892	0.99088	BBB+
NPRL 1893	0.99025	BBB+
NPRL 1894	0.99385	BBB+
NPRL 1895	0.99859	BBB+
NPRL 1896	0.98566	BBB+
NPRL 1897	0.98787	BBB+
NPRL 1898	0.98640	BBB+
NPRL 1899	0.96279	BBB+
NPRL 19	0.08060	BBB-
NPRL 190	0.43166	BBB-
NPRL 1900	0.99044	BBB+
NPRL 1901	0.99628	BBB+
NPRL 1902	0.99128	BBB+
NPRL 1903	0.99820	BBB+
NPRL 1904	0.93922	BBB+
NPRL 1905	0.98250	BBB+
NPRL 1906	0.99374	BBB+
NPRL 1907	0.95452	BBB+
NPRL 1908	0.95867	BBB+
NPRL 1909	0.47885	BBB-
NPRL 191	0.74680	BBB+
NPRL 1910	0.99244	BBB+
NPRL 1911	0.98503	BBB+

NPRL 1912	0.99085	BBB+
NPRL 1913	0.97538	BBB+
NPRL 1914	0.85892	BBB+
NPRL 1915	0.92179	BBB+
NPRL 1919	0.94039	BBB+
NPRL 1920	0.93215	BBB+
NPRL 1921	0.93499	BBB+
NPRL 1922	0.93369	BBB+
NPRL 1923	0.98308	BBB+
NPRL 1924	0.88542	BBB+
NPRL 1925	0.86756	BBB+
NPRL 1926	0.90962	BBB+
NPRL 1927	0.96695	BBB+
NPRL 1929	0.98297	BBB+
NPRL 193	0.90590	BBB+
NPRL 1932	0.89355	BBB+
NPRL 1933	0.90590	BBB+
NPRL 1935	0.82961	BBB+
NPRL 1936	0.78987	BBB+
NPRL 1937	0.99099	BBB+
NPRL 1938	0.76048	BBB+
NPRL 1939	0.78055	BBB+
NPRL 194	0.61286	BBB+
NPRL 1940	0.76889	BBB+
NPRL 1941	0.26338	BBB-
NPRL 1942	0.63785	BBB+
NPRL 1943	0.95100	BBB+
NPRL 1944	0.48932	BBB-
NPRL 1946	0.99025	BBB+
NPRL 1947	0.84046	BBB+
NPRL 1948	0.45773	BBB-
NPRL 1949	0.87120	BBB+
NPRL 195	0.99112	BBB+
NPRL 1950	0.99785	BBB+
NPRL 1951	0.77047	BBB+
NPRL 1952	0.97738	BBB+
NPRL 1953	0.91440	BBB+
NPRL 1954	0.60857	BBB+
NPRL 1955	0.98373	BBB+
NPRL 1956	0.94809	BBB+
NPRL 1957	0.99494	BBB+
NPRL 1958	0.99821	BBB+
NPRL 1959	0.99704	BBB+
NPRL 196	0.14981	BBB-
NPRL 1960	0.99915	BBB+
NPRL 1961	0.99712	BBB+
NPRL 1962	0.92111	BBB+
NPRL 1963	0.96813	BBB+
NPRL 1964	0.98849	BBB+
NPRL 1965	0.99620	BBB+
NPRL 1966	0.98964	BBB+
NPRL 1967	0.99495	BBB+
NPRL 1968	0.99873	BBB+
NPRL 1969	0.28499	BBB-
NPRL 197	0.91230	BBB+
NPRL 1970	0.91184	BBB+
NPRL 1971	0.96584	BBB+

NPRL 1972	0.97788	BBB+
NPRL 1973	0.29363	BBB-
NPRL 1974	0.32816	BBB-
NPRL 1975	0.42572	BBB-
NPRL 1976	0.71959	BBB+
NPRL 1977	0.00026	BBB-
NPRL 1979	0.84527	BBB+
NPRL 1980	0.81497	BBB+
NPRL 1981	0.49912	BBB-
NPRL 1982	0.00051	BBB-
NPRL 1983	0.97692	BBB+
NPRL 1984	0.99155	BBB+
NPRL 1985	0.90969	BBB+
NPRL 1986	0.85231	BBB+
NPRL 1987	0.05821	BBB-
NPRL 1988	0.00699	BBB-
NPRL 1989	0.00765	BBB-
NPRL 199	0.14140	BBB-
NPRL 1990	0.00196	BBB-
NPRL 1991	0.00270	BBB-
NPRL 1992	0.00059	BBB-
NPRL 1993	0.00766	BBB-
NPRL 1994	0.00554	BBB-
NPRL 1995	0.01046	BBB-
NPRL 1996	0.43470	BBB-
NPRL 1997	0.05094	BBB-
NPRL 1998	0.05070	BBB-
NPRL 2	0.97381	BBB+
NPRL 20	0.36651	BBB-
NPRL 2001	0.07590	BBB-
NPRL 2002	0.93535	BBB+
NPRL 2003	0.38807	BBB-
NPRL 2004	0.77195	BBB+
NPRL 2005	0.99639	BBB+
NPRL 2006	0.92663	BBB+
NPRL 2007	0.99929	BBB+
NPRL 2008	0.75623	BBB+
NPRL 2009	0.99465	BBB+
NPRL 201	0.99079	BBB+
NPRL 2010	0.97569	BBB+
NPRL 2011	0.99811	BBB+
NPRL 2014	0.89672	BBB+
NPRL 2015	0.45737	BBB-
NPRL 2016	0.01329	BBB-
NPRL 2017	0.12955	BBB-
NPRL 2018	0.04264	BBB-
NPRL 2019	0.02097	BBB-
NPRL 202	0.00588	BBB-
NPRL 2020	0.00726	BBB-
NPRL 2021	0.00058	BBB-
NPRL 2023	0.68606	BBB+
NPRL 2025	0.97090	BBB+
NPRL 2026	0.99583	BBB+
NPRL 2027	0.98770	BBB+
NPRL 2028	0.99260	BBB+
NPRL 2029	0.99832	BBB+
NPRL 203	0.08528	BBB-

NPRL 2032	0.60327	BBB+
NPRL 2033	0.55846	BBB+
NPRL 2034	0.85131	BBB+
NPRL 2037	0.89930	BBB+
NPRL 2038	0.95744	BBB+
NPRL 2039	0.99683	BBB+
NPRL 204	0.61273	BBB+
NPRL 2042	0.82845	BBB+
NPRL 2043	0.89831	BBB+
NPRL 2049	0.08297	BBB-
NPRL 205	0.94949	BBB+
NPRL 2050	0.97965	BBB+
NPRL 2051	0.99571	BBB+
NPRL 2052	0.61317	BBB+
NPRL 2054	0.87756	BBB+
NPRL 2055	0.97930	BBB+
NPRL 2056	0.94031	BBB+
NPRL 2057	0.89095	BBB+
NPRL 2058	0.96087	BBB+
NPRL 2059	0.99636	BBB+
NPRL 206	0.83441	BBB+
NPRL 2061	0.96044	BBB+
NPRL 2062	0.96433	BBB+
NPRL 2065	0.22042	BBB-
NPRL 2066	0.97673	BBB+
NPRL 2067	0.97403	BBB+
NPRL 2068	0.09509	BBB-
NPRL 2069	0.91547	BBB+
NPRL 207	0.86265	BBB+
NPRL 2070	0.85740	BBB+
NPRL 2071	0.99415	BBB+
NPRL 2072	0.93342	BBB+
NPRL 2073	0.98040	BBB+
NPRL 2074	0.59798	BBB+
NPRL 2076	0.95042	BBB+
NPRL 2077	0.99547	BBB+
NPRL 2078	0.95717	BBB+
NPRL 2079	0.99680	BBB+
NPRL 208	0.00442	BBB-
NPRL 2080	0.99906	BBB+
NPRL 2083	0.99567	BBB+
NPRL 2085	0.57285	BBB+
NPRL 2086	0.40926	BBB-
NPRL 2088	0.86612	BBB+
NPRL 2089	0.95812	BBB+
NPRL 209	0.01478	BBB-
NPRL 2090	0.95429	BBB+
NPRL 2091	0.55268	BBB+
NPRL 2092	0.52262	BBB+
NPRL 2093	0.99677	BBB+
NPRL 2096	0.79407	BBB+
NPRL 2097	0.96497	BBB+
NPRL 2098	0.91691	BBB+
NPRL 2099	0.99574	BBB+
NPRL 21	0.59550	BBB+
NPRL 210	0.00596	BBB-
NPRL 2100	0.97602	BBB+

NPRL 2101	0.93755	BBB+
NPRL 2102	0.75019	BBB+
NPRL 2103	0.88266	BBB+
NPRL 2105	0.98448	BBB+
NPRL 211	0.97950	BBB+
NPRL 2112	0.22356	BBB-
NPRL 2113	0.64917	BBB+
NPRL 2114	0.03297	BBB-
NPRL 2115	0.49949	BBB-
NPRL 2116	0.00410	BBB-
NPRL 2117	0.16693	BBB-
NPRL 2118	0.00927	BBB-
NPRL 2119	0.50342	BBB+
NPRL 212	0.83444	BBB+
NPRL 2120	0.48277	BBB-
NPRL 2121	0.60202	BBB+
NPRL 2122	0.67869	BBB+
NPRL 2123	0.79036	BBB+
NPRL 2124	0.00108	BBB-
NPRL 2125	0.00060	BBB-
NPRL 2127	0.98529	BBB+
NPRL 2128	0.92299	BBB+
NPRL 2129	0.99212	BBB+
NPRL 213	0.00110	BBB-
NPRL 2130	0.99662	BBB+
NPRL 2131	0.19914	BBB-
NPRL 2132	0.10321	BBB-
NPRL 2134	0.56425	BBB+
NPRL 2135	0.76088	BBB+
NPRL 2137	0.91147	BBB+
NPRL 2139	0.97641	BBB+
NPRL 214	0.00009	BBB-
NPRL 2140	0.15799	BBB-
NPRL 2144	0.00326	BBB-
NPRL 2145	0.47840	BBB-
NPRL 2146	0.98342	BBB+
NPRL 2147	0.80111	BBB+
NPRL 2148	0.99787	BBB+
NPRL 2149	0.42259	BBB-
NPRL 215	0.01428	BBB-
NPRL 2150	0.98785	BBB+
NPRL 2151	0.98989	BBB+
NPRL 2153	0.60756	BBB+
NPRL 2154	0.71722	BBB+
NPRL 2155	0.77251	BBB+
NPRL 2156	0.92534	BBB+
NPRL 2158	0.98171	BBB+
NPRL 216	0.01259	BBB-
NPRL 217	0.98146	BBB+
NPRL 2170	0.00119	BBB-
NPRL 2171	0.19386	BBB-
NPRL 2172	0.50270	BBB+
NPRL 2173	0.33111	BBB-
NPRL 2174	0.51110	BBB+
NPRL 2175	0.22120	BBB-
NPRL 2176	0.03131	BBB-
NPRL 2177	0.04727	BBB-

NPRL 2178	0.18363	BBB-
NPRL 2179	0.02676	BBB-
NPRL 218	0.90551	BBB+
NPRL 2180	0.11420	BBB-
NPRL 2181	0.00247	BBB-
NPRL 2182	0.00575	BBB-
NPRL 2183	0.20019	BBB-
NPRL 2184	0.38864	BBB-
NPRL 2185	0.02072	BBB-
NPRL 2186	0.17678	BBB-
NPRL 2187	0.00008	BBB-
NPRL 2188	0.97715	BBB+
NPRL 2189	0.95454	BBB+
NPRL 219	0.57653	BBB+
NPRL 2190	0.17937	BBB-
NPRL 2191	0.88226	BBB+
NPRL 2192	0.83793	BBB+
NPRL 2193	0.03838	BBB-
NPRL 2194	0.94541	BBB+
NPRL 2195	0.99362	BBB+
NPRL 2196	0.98876	BBB+
NPRL 2197	0.99034	BBB+
NPRL 2198	0.98557	BBB+
NPRL 2199	0.98241	BBB+
NPRL 22	0.04968	BBB-
NPRL 220	0.51481	BBB+
NPRL 2200	0.99623	BBB+
NPRL 2201	0.78525	BBB+
NPRL 2202	0.82007	BBB+
NPRL 2203	0.99024	BBB+
NPRL 2204	0.99941	BBB+
NPRL 2205	0.99691	BBB+
NPRL 2206	0.92778	BBB+
NPRL 2207	0.41614	BBB-
NPRL 221	0.99580	BBB+
NPRL 222	0.00389	BBB-
NPRL 223	0.00066	BBB-
NPRL 225	0.00309	BBB-
NPRL 227	0.00052	BBB-
NPRL 228	0.00617	BBB-
NPRL 230	0.00357	BBB-
NPRL 231	0.00242	BBB-
NPRL 232	0.00061	BBB-
NPRL 235	0.00132	BBB-
NPRL 237	0.96865	BBB+
NPRL 239	0.00542	BBB-
NPRL 24	0.11048	BBB-
NPRL 240	0.00542	BBB-
NPRL 242	0.01133	BBB-
NPRL 245	0.01024	BBB-
NPRL 246	0.00025	BBB-
NPRL 248	0.00022	BBB-
NPRL 249	0.00371	BBB-
NPRL 25	0.75601	BBB+
NPRL 250	0.00038	BBB-
NPRL 252	0.00149	BBB-
NPRL 258	0.00699	BBB-

NPRL 259	0.00185	BBB-
NPRL 26	0.53269	BBB+
NPRL 260	0.93570	BBB+
NPRL 262	0.00279	BBB-
NPRL 263	0.00375	BBB-
NPRL 264	0.01467	BBB-
NPRL 265	0.01978	BBB-
NPRL 268	0.00034	BBB-
NPRL 27	0.99204	BBB+
NPRL 270	0.02159	BBB-
NPRL 271	0.01394	BBB-
NPRL 272	0.85339	BBB+
NPRL 274	0.00291	BBB-
NPRL 276	0.77370	BBB+
NPRL 277	0.77119	BBB+
NPRL 28	0.82767	BBB+
NPRL 281	0.91850	BBB+
NPRL 282	0.97553	BBB+
NPRL 283	0.98217	BBB+
NPRL 284	0.88698	BBB+
NPRL 285	0.89075	BBB+
NPRL 286	0.88285	BBB+
NPRL 287	0.64775	BBB+
NPRL 288	0.69933	BBB+
NPRL 289	0.87832	BBB+
NPRL 29	0.47586	BBB-
NPRL 290	0.89342	BBB+
NPRL 291	0.89110	BBB+
NPRL 293	0.98047	BBB+
NPRL 294	0.71138	BBB+
NPRL 295	0.99321	BBB+
NPRL 296	0.87159	BBB+
NPRL 297	0.97889	BBB+
NPRL 298	0.39758	BBB-
NPRL 299	0.70254	BBB+
NPRL 3	0.93633	BBB+
NPRL 30	0.69461	BBB+
NPRL 300	0.40719	BBB-
NPRL 301	0.99541	BBB+
NPRL 302	0.96815	BBB+
NPRL 305	0.97494	BBB+
NPRL 306	0.98961	BBB+
NPRL 308	0.01503	BBB-
NPRL 309	0.99522	BBB+
NPRL 31	0.93275	BBB+
NPRL 310	0.46809	BBB-
NPRL 311	0.57357	BBB+
NPRL 313	0.40129	BBB-
NPRL 316	0.02825	BBB-
NPRL 318	0.98480	BBB+
NPRL 32	0.69133	BBB+
NPRL 320	0.27242	BBB-
NPRL 322	0.93192	BBB+
NPRL 325	0.46972	BBB-
NPRL 329	0.74687	BBB+
NPRL 331	0.04193	BBB-
NPRL 332	0.26231	BBB-

NPRL 333	0.00224	BBB-
NPRL 334	0.04633	BBB-
NPRL 335	0.81456	BBB+
NPRL 336	0.03190	BBB-
NPRL 337	0.00345	BBB-
NPRL 338	0.15844	BBB-
NPRL 339	0.00055	BBB-
NPRL 34	0.71068	BBB+
NPRL 340	0.00065	BBB-
NPRL 341	0.25872	BBB-
NPRL 342	0.49580	BBB-
NPRL 343	0.36289	BBB-
NPRL 344	0.05342	BBB-
NPRL 345	0.19883	BBB-
NPRL 346	0.18085	BBB-
NPRL 347	0.04903	BBB-
NPRL 348	0.42188	BBB-
NPRL 349	0.04098	BBB-
NPRL 35	0.03356	BBB-
NPRL 350	0.12782	BBB-
NPRL 351	0.24160	BBB-
NPRL 352	0.37461	BBB-
NPRL 353	0.97364	BBB+
NPRL 354	0.00105	BBB-
NPRL 355	0.01176	BBB-
NPRL 356	0.65009	BBB+
NPRL 357	0.85576	BBB+
NPRL 358	0.99815	BBB+
NPRL 359	0.05457	BBB-
NPRL 36	0.96566	BBB+
NPRL 360	0.34855	BBB-
NPRL 361	0.72752	BBB+
NPRL 362	0.00821	BBB-
NPRL 363	0.00152	BBB-
NPRL 364	0.20506	BBB-
NPRL 365	0.03973	BBB-
NPRL 366	0.03587	BBB-
NPRL 367	0.02913	BBB-
NPRL 368	0.16981	BBB-
NPRL 369	0.02901	BBB-
NPRL 37	0.52481	BBB+
NPRL 370	0.00015	BBB-
NPRL 371	0.08912	BBB-
NPRL 372	0.66676	BBB+
NPRL 373	0.58389	BBB+
NPRL 374	0.22753	BBB-
NPRL 375	0.35442	BBB-
NPRL 378	0.84966	BBB+
NPRL 379	0.70392	BBB+
NPRL 38	0.98147	BBB+
NPRL 380	0.64723	BBB+
NPRL 381	0.96675	BBB+
NPRL 382	0.00449	BBB-
NPRL 383	0.86596	BBB+
NPRL 384	0.50540	BBB+
NPRL 385	0.74381	BBB+
NPRL 386	0.70066	BBB+

NPRL 387	0.45634	BBB-
NPRL 388	0.26000	BBB-
NPRL 389	0.52846	BBB+
NPRL 39	0.13512	BBB-
NPRL 390	0.08725	BBB-
NPRL 391	0.10522	BBB-
NPRL 392	0.02918	BBB-
NPRL 394	0.52993	BBB+
NPRL 395	0.92443	BBB+
NPRL 396	0.88857	BBB+
NPRL 397	0.40572	BBB-
NPRL 398	0.89403	BBB+
NPRL 399	0.08398	BBB-
NPRL 4	0.80067	BBB+
NPRL 40	0.04883	BBB-
NPRL 400	0.07554	BBB-
NPRL 401	0.05294	BBB-
NPRL 403	0.02603	BBB-
NPRL 404	0.43413	BBB-
NPRL 405	0.82878	BBB+
NPRL 406	0.81804	BBB+
NPRL 407	0.94766	BBB+
NPRL 408	0.82478	BBB+
NPRL 409	0.75347	BBB+
NPRL 41	0.91799	BBB+
NPRL 410	0.10425	BBB-
NPRL 411	0.04773	BBB-
NPRL 413	0.08528	BBB-
NPRL 414	0.17353	BBB-
NPRL 415	0.43736	BBB-
NPRL 416	0.30442	BBB-
NPRL 417	0.29741	BBB-
NPRL 418	0.68639	BBB+
NPRL 419	0.39254	BBB-
NPRL 42	0.94813	BBB+
NPRL 420	0.20418	BBB-
NPRL 421	0.17477	BBB-
NPRL 425	0.00945	BBB-
NPRL 426	0.53037	BBB+
NPRL 427	0.64416	BBB+
NPRL 429	0.91586	BBB+
NPRL 43	0.63884	BBB+
NPRL 430	0.93121	BBB+
NPRL 431	0.32376	BBB-
NPRL 432	0.91257	BBB+
NPRL 433	0.67518	BBB+
NPRL 434	0.27250	BBB-
NPRL 435	0.97494	BBB+
NPRL 436	0.99240	BBB+
NPRL 437	0.88665	BBB+
NPRL 438	0.03158	BBB-
NPRL 439	0.02613	BBB-
NPRL 44	0.97054	BBB+
NPRL 440	0.65465	BBB+
NPRL 441	0.24351	BBB-
NPRL 442	0.05721	BBB-
NPRL 443	0.15350	BBB-

NPRL 444	0.17857	BBB-
NPRL 445	0.79978	BBB+
NPRL 446	0.87862	BBB+
NPRL 447	0.98185	BBB+
NPRL 448	0.00546	BBB-
NPRL 449	0.62246	BBB+
NPRL 45	0.79597	BBB+
NPRL 451	0.10454	BBB-
NPRL 452	0.11871	BBB-
NPRL 453	0.01680	BBB-
NPRL 454	0.28519	BBB-
NPRL 455	0.76101	BBB+
NPRL 456	0.63324	BBB+
NPRL 457	0.24213	BBB-
NPRL 459	0.51539	BBB+
NPRL 46	0.67793	BBB+
NPRL 460	0.24883	BBB-
NPRL 461	0.01366	BBB-
NPRL 462	0.04844	BBB-
NPRL 463	0.24888	BBB-
NPRL 464	0.01744	BBB-
NPRL 466	0.09536	BBB-
NPRL 467	0.18568	BBB-
NPRL 468	0.00718	BBB-
NPRL 469	0.07958	BBB-
NPRL 47	0.96751	BBB+
NPRL 470	0.00192	BBB-
NPRL 471	0.00327	BBB-
NPRL 472	0.06499	BBB-
NPRL 473	0.17257	BBB-
NPRL 474	0.94615	BBB+
NPRL 477	0.80287	BBB+
NPRL 478	0.39996	BBB-
NPRL 479	0.17976	BBB-
NPRL 48	0.97351	BBB+
NPRL 481	0.92420	BBB+
NPRL 482	0.81125	BBB+
NPRL 484	0.70336	BBB+
NPRL 49	0.39908	BBB-
NPRL 491	0.02491	BBB-
NPRL 492	0.92756	BBB+
NPRL 493	0.57034	BBB+
NPRL 495	0.98427	BBB+
NPRL 496	0.81913	BBB+
NPRL 497	0.92160	BBB+
NPRL 498	0.56267	BBB+
NPRL 499	0.78675	BBB+
NPRL 5	0.98923	BBB+
NPRL 50	0.66749	BBB+
NPRL 501	0.57001	BBB+
NPRL 502	0.65512	BBB+
NPRL 503	0.34566	BBB-
NPRL 504	0.35356	BBB-
NPRL 505	0.56631	BBB+
NPRL 506	0.98093	BBB+
NPRL 507	0.98386	BBB+
NPRL 508	0.29468	BBB-

NPRL 509	0.37675	BBB-
NPRL 510	0.78661	BBB+
NPRL 511	0.55845	BBB+
NPRL 512	0.95693	BBB+
NPRL 513	0.80195	BBB+
NPRL 514	0.74587	BBB+
NPRL 516	0.62469	BBB+
NPRL 52	0.18844	BBB-
NPRL 522	0.77151	BBB+
NPRL 524	0.85012	BBB+
NPRL 526	0.99576	BBB+
NPRL 527	0.87981	BBB+
NPRL 529	0.98777	BBB+
NPRL 53	0.91757	BBB+
NPRL 530	0.89159	BBB+
NPRL 532	0.99389	BBB+
NPRL 534	0.99431	BBB+
NPRL 535	0.88405	BBB+
NPRL 536	0.93616	BBB+
NPRL 537	0.88937	BBB+
NPRL 540	0.98718	BBB+
NPRL 541	0.91079	BBB+
NPRL 543	0.95187	BBB+
NPRL 544	0.62214	BBB+
NPRL 545	0.95396	BBB+
NPRL 546	0.99574	BBB+
NPRL 548	0.87154	BBB+
NPRL 55	0.98159	BBB+
NPRL 551	0.76043	BBB+
NPRL 552	0.57034	BBB+
NPRL 553	0.92082	BBB+
NPRL 554	0.85054	BBB+
NPRL 555	0.98084	BBB+
NPRL 556	0.25833	BBB-
NPRL 557	0.92767	BBB+
NPRL 558	0.03205	BBB-
NPRL 559	0.66994	BBB+
NPRL 56	0.96235	BBB+
NPRL 560	0.00162	BBB-
NPRL 561	0.81615	BBB+
NPRL 562	0.89616	BBB+
NPRL 564	0.95420	BBB+
NPRL 565	0.81200	BBB+
NPRL 566	0.45395	BBB-
NPRL 568	0.88962	BBB+
NPRL 570	0.01156	BBB-
NPRL 571	0.87769	BBB+
NPRL 572	0.05633	BBB-
NPRL 573	0.01442	BBB-
NPRL 574	0.05886	BBB-
NPRL 576	0.04254	BBB-
NPRL 577	0.00618	BBB-
NPRL 578	0.00582	BBB-
NPRL 5781	0.02001	BBB-
NPRL 5782	0.00494	BBB-
NPRL 5783	0.00358	BBB-
NPRL 5784	0.00794	BBB-

NPRL 5785	0.00071	BBB-
NPRL 5786	0.01835	BBB-
NPRL 5787	0.00211	BBB-
NPRL 5788	0.00272	BBB-
NPRL 5789	0.01191	BBB-
NPRL 579	0.00229	BBB-
NPRL 5790	0.00468	BBB-
NPRL 5791	0.03091	BBB-
NPRL 5792	0.03628	BBB-
NPRL 5793	0.03926	BBB-
NPRL 5794	0.02578	BBB-
NPRL 5795	0.02413	BBB-
NPRL 5796	0.01759	BBB-
NPRL 5797	0.08373	BBB-
NPRL 5798	0.02421	BBB-
NPRL 5799	0.02572	BBB-
NPRL 580	0.00088	BBB-
NPRL 5800	0.00557	BBB-
NPRL 5801	0.00627	BBB-
NPRL 5802	0.00977	BBB-
NPRL 5803	0.04112	BBB-
NPRL 5804	0.07162	BBB-
NPRL 5805	0.10548	BBB-
NPRL 5806	0.04290	BBB-
NPRL 5807	0.00325	BBB-
NPRL 5808	0.15762	BBB-
NPRL 5809	0.00093	BBB-
NPRL 5810	0.03380	BBB-
NPRL 5811	0.02523	BBB-
NPRL 5814	0.41630	BBB-
NPRL 5815	0.24097	BBB-
NPRL 5816	0.01610	BBB-
NPRL 5817	0.45050	BBB-
NPRL 5818	0.02583	BBB-
NPRL 5819	0.00218	BBB-
NPRL 582	0.06583	BBB-
NPRL 5820	0.04409	BBB-
NPRL 5821	0.00865	BBB-
NPRL 5822	0.02230	BBB-
NPRL 5823	0.00378	BBB-
NPRL 5824	0.00242	BBB-
NPRL 5825	0.00370	BBB-
NPRL 5826	0.00193	BBB-
NPRL 5827	0.00072	BBB-
NPRL 5828	0.00166	BBB-
NPRL 5829	0.00211	BBB-
NPRL 583	0.01509	BBB-
NPRL 5830	0.00082	BBB-
NPRL 5831	0.00245	BBB-
NPRL 5832	0.01109	BBB-
NPRL 5833	0.16617	BBB-
NPRL 5834	0.08154	BBB-
NPRL 5835	0.04147	BBB-
NPRL 5836	0.02421	BBB-
NPRL 5837	0.03079	BBB-
NPRL 5838	0.00489	BBB-
NPRL 5839	0.05746	BBB-

NPRL 584	0.03576	BBB-
NPRL 5840	0.08547	BBB-
NPRL 5841	0.10930	BBB-
NPRL 5842	0.03268	BBB-
NPRL 5843	0.04212	BBB-
NPRL 5844	0.04313	BBB-
NPRL 5845	0.08902	BBB-
NPRL 5846	0.01574	BBB-
NPRL 5847	0.00956	BBB-
NPRL 5848	0.01853	BBB-
NPRL 585	0.02073	BBB-
NPRL 5864	0.00082	BBB-
NPRL 5865	0.00019	BBB-
NPRL 5866	0.00166	BBB-
NPRL 5867	0.01558	BBB-
NPRL 5868	0.02524	BBB-
NPRL 5869	0.05555	BBB-
NPRL 587	0.20025	BBB-
NPRL 5870	0.00015	BBB-
NPRL 5871	0.00061	BBB-
NPRL 5872	0.00049	BBB-
NPRL 5873	0.00017	BBB-
NPRL 5874	0.00049	BBB-
NPRL 5875	0.00405	BBB-
NPRL 5876	0.00015	BBB-
NPRL 5877	0.00003	BBB-
NPRL 5878	0.00016	BBB-
NPRL 5879	0.00044	BBB-
NPRL 588	0.99820	BBB+
NPRL 5880	0.00020	BBB-
NPRL 5881	0.01816	BBB-
NPRL 5882	0.00040	BBB-
NPRL 5883	0.00008	BBB-
NPRL 5884	0.00033	BBB-
NPRL 5885	0.00113	BBB-
NPRL 5886	0.00621	BBB-
NPRL 5887	0.00356	BBB-
NPRL 589	0.88152	BBB+
NPRL 5890	0.05230	BBB-
NPRL 5891	0.00105	BBB-
NPRL 5892	0.00149	BBB-
NPRL 5893	0.00439	BBB-
NPRL 5894	0.00421	BBB-
NPRL 5895	0.00361	BBB-
NPRL 5896	0.00257	BBB-
NPRL 5897	0.00251	BBB-
NPRL 5898	0.00433	BBB-
NPRL 5899	0.00323	BBB-
NPRL 59	0.85671	BBB+
NPRL 590	0.43878	BBB-
NPRL 5900	0.00088	BBB-
NPRL 5901	0.00279	BBB-
NPRL 5902	0.00937	BBB-
NPRL 5903	0.00383	BBB-
NPRL 5904	0.00572	BBB-
NPRL 5905	0.00404	BBB-
NPRL 5906	0.00176	BBB-

NPRL 5907	0.00437	BBB-
NPRL 5908	0.00259	BBB-
NPRL 5909	0.00178	BBB-
NPRL 591	0.83005	BBB+
NPRL 5910	0.00171	BBB-
NPRL 5911	0.00828	BBB-
NPRL 5912	0.00967	BBB-
NPRL 5913	0.51215	BBB+
NPRL 5916	0.00201	BBB-
NPRL 5917	0.00292	BBB-
NPRL 5918	0.00766	BBB-
NPRL 5919	0.00124	BBB-
NPRL 592	0.95813	BBB+
NPRL 5920	0.00080	BBB-
NPRL 5921	0.00229	BBB-
NPRL 5922	0.00094	BBB-
NPRL 5923	0.00246	BBB-
NPRL 5924	0.00715	BBB-
NPRL 5925	0.00164	BBB-
NPRL 5926	0.00063	BBB-
NPRL 5927	0.00770	BBB-
NPRL 5928	0.00163	BBB-
NPRL 5929	0.00188	BBB-
NPRL 5930	0.00199	BBB-
NPRL 5931	0.00147	BBB-
NPRL 5932	0.00461	BBB-
NPRL 5933	0.05932	BBB-
NPRL 5934	0.03851	BBB-
NPRL 5935	0.00813	BBB-
NPRL 5936	0.02399	BBB-
NPRL 5937	0.03933	BBB-
NPRL 5938	0.03303	BBB-
NPRL 5939	0.02948	BBB-
NPRL 594	0.99772	BBB+
NPRL 5940	0.02164	BBB-
NPRL 5941	0.05605	BBB-
NPRL 5942	0.08220	BBB-
NPRL 5943	0.07086	BBB-
NPRL 5944	0.02412	BBB-
NPRL 5945	0.01996	BBB-
NPRL 5946	0.05425	BBB-
NPRL 5947	0.02051	BBB-
NPRL 5948	0.23447	BBB-
NPRL 5949	0.08805	BBB-
NPRL 5950	0.14165	BBB-
NPRL 5951	0.06182	BBB-
NPRL 5952	0.10917	BBB-
NPRL 5953	0.06843	BBB-
NPRL 5954	0.21607	BBB-
NPRL 5955	0.05959	BBB-
NPRL 5956	0.23674	BBB-
NPRL 5957	0.02722	BBB-
NPRL 5958	0.00531	BBB-
NPRL 5959	0.00292	BBB-
NPRL 5960	0.00701	BBB-
NPRL 5961	0.19797	BBB-
NPRL 5962	0.01348	BBB-

NPRL 5963	0.03983	BBB-
NPRL 5964	0.06399	BBB-
NPRL 5965	0.03642	BBB-
NPRL 5966	0.00407	BBB-
NPRL 5967	0.01959	BBB-
NPRL 5968	0.00564	BBB-
NPRL 5969	0.00548	BBB-
NPRL 597	0.89152	BBB+
NPRL 5970	0.53841	BBB+
NPRL 5971	0.04943	BBB-
NPRL 5972	0.00079	BBB-
NPRL 5973	0.00217	BBB-
NPRL 5974	0.00471	BBB-
NPRL 5975	0.03016	BBB-
NPRL 5976	0.02029	BBB-
NPRL 5977	0.17564	BBB-
NPRL 5978	0.56230	BBB+
NPRL 5979	0.14157	BBB-
NPRL 598	0.73963	BBB+
NPRL 5980	0.02862	BBB-
NPRL 5981	0.03264	BBB-
NPRL 5982	0.00715	BBB-
NPRL 5983	0.03576	BBB-
NPRL 5984	0.14279	BBB-
NPRL 5985	0.00726	BBB-
NPRL 5986	0.00118	BBB-
NPRL 5988	0.00308	BBB-
NPRL 5989	0.00835	BBB-
NPRL 599	0.99064	BBB+
NPRL 5990	0.02571	BBB-
NPRL 5991	0.00563	BBB-
NPRL 5992	0.01353	BBB-
NPRL 5993	0.00842	BBB-
NPRL 5994	0.02388	BBB-
NPRL 5995	0.00851	BBB-
NPRL 5996	0.00403	BBB-
NPRL 5997	0.03975	BBB-
NPRL 5998	0.00833	BBB-
NPRL 5999	0.04607	BBB-
NPRL 6	0.21846	BBB-
NPRL 60	0.96841	BBB+
NPRL 6000	0.03117	BBB-
NPRL 6001	0.03293	BBB-
NPRL 6002	0.04203	BBB-
NPRL 6003	0.01265	BBB-
NPRL 6004	0.01701	BBB-
NPRL 6005	0.04903	BBB-
NPRL 6006	0.04114	BBB-
NPRL 6007	0.00178	BBB-
NPRL 6008	0.01227	BBB-
NPRL 6009	0.00663	BBB-
NPRL 601	0.93767	BBB+
NPRL 6010	0.02557	BBB-
NPRL 6011	0.04742	BBB-
NPRL 6012	0.03664	BBB-
NPRL 6013	0.01692	BBB-
NPRL 6014	0.33950	BBB-

NPRL 6015	0.00263	BBB-
NPRL 6016	0.00046	BBB-
NPRL 6017	0.00202	BBB-
NPRL 6018	0.00316	BBB-
NPRL 6019	0.08663	BBB-
NPRL 602	0.94541	BBB+
NPRL 6020	0.01307	BBB-
NPRL 6021	0.04167	BBB-
NPRL 6022	0.07687	BBB-
NPRL 6023	0.04859	BBB-
NPRL 6024	0.05833	BBB-
NPRL 6025	0.03498	BBB-
NPRL 6026	0.09618	BBB-
NPRL 6027	0.00596	BBB-
NPRL 6028	0.00251	BBB-
NPRL 6029	0.00129	BBB-
NPRL 603	0.96774	BBB+
NPRL 6030	0.02024	BBB-
NPRL 6031	0.10084	BBB-
NPRL 6032	0.02428	BBB-
NPRL 6033	0.03661	BBB-
NPRL 6034	0.00059	BBB-
NPRL 6035	0.00126	BBB-
NPRL 6036	0.00055	BBB-
NPRL 6037	0.01638	BBB-
NPRL 6038	0.01460	BBB-
NPRL 6039	0.00172	BBB-
NPRL 6040	0.00113	BBB-
NPRL 6041	0.00348	BBB-
NPRL 6042	0.00820	BBB-
NPRL 6043	0.00081	BBB-
NPRL 6044	0.00357	BBB-
NPRL 6045	0.10630	BBB-
NPRL 6046	0.07063	BBB-
NPRL 6047	0.11301	BBB-
NPRL 6048	0.28920	BBB-
NPRL 6049	0.20355	BBB-
NPRL 6050	0.26426	BBB-
NPRL 6051	0.27059	BBB-
NPRL 6052	0.17637	BBB-
NPRL 6053	0.05008	BBB-
NPRL 6054	0.00728	BBB-
NPRL 6055	0.06805	BBB-
NPRL 6056	0.01397	BBB-
NPRL 6057	0.01051	BBB-
NPRL 6058	0.06530	BBB-
NPRL 6059	0.16422	BBB-
NPRL 6060	0.04962	BBB-
NPRL 6062	0.17673	BBB-
NPRL 6063	0.00140	BBB-
NPRL 6064	0.00991	BBB-
NPRL 6065	0.00406	BBB-
NPRL 6066	0.03380	BBB-
NPRL 6067	0.12536	BBB-
NPRL 6068	0.06564	BBB-
NPRL 6069	0.02543	BBB-
NPRL 6070	0.00817	BBB-

NPRL 6071	0.01582	BBB-
NPRL 6072	0.01450	BBB-
NPRL 6073	0.04595	BBB-
NPRL 6074	0.05996	BBB-
NPRL 6075	0.01843	BBB-
NPRL 6076	0.00140	BBB-
NPRL 6077	0.00092	BBB-
NPRL 6078	0.00342	BBB-
NPRL 6079	0.00213	BBB-
NPRL 6080	0.01293	BBB-
NPRL 6081	0.00475	BBB-
NPRL 6082	0.00531	BBB-
NPRL 6083	0.00961	BBB-
NPRL 6084	0.38289	BBB-
NPRL 6085	0.83337	BBB+
NPRL 6086	0.15643	BBB-
NPRL 6087	0.01204	BBB-
NPRL 6088	0.03344	BBB-
NPRL 6089	0.17443	BBB-
NPRL 6090	0.03093	BBB-
NPRL 6091	0.29883	BBB-
NPRL 6092	0.28442	BBB-
NPRL 6093	0.29589	BBB-
NPRL 6094	0.16706	BBB-
NPRL 6095	0.87244	BBB+
NPRL 6097	0.99674	BBB+
NPRL 6098	0.97778	BBB+
NPRL 6099	0.95092	BBB+
NPRL 61	0.54800	BBB+
NPRL 6100	0.99667	BBB+
NPRL 6101	0.19688	BBB-
NPRL 6102	0.44637	BBB-
NPRL 6103	0.31061	BBB-
NPRL 6104	0.38374	BBB-
NPRL 6105	0.59114	BBB+
NPRL 6106	0.47341	BBB-
NPRL 6107	0.34001	BBB-
NPRL 6108	0.41650	BBB-
NPRL 6109	0.24049	BBB-
NPRL 6110	0.34807	BBB-
NPRL 6111	0.36734	BBB-
NPRL 6112	0.38833	BBB-
NPRL 6113	0.44407	BBB-
NPRL 6114	0.57872	BBB+
NPRL 6115	0.49941	BBB-
NPRL 6116	0.63515	BBB+
NPRL 6117	0.06312	BBB-
NPRL 6118	0.04030	BBB-
NPRL 6119	0.03529	BBB-
NPRL 6120	0.01999	BBB-
NPRL 6121	0.91775	BBB+
NPRL 6122	0.93319	BBB+
NPRL 6123	0.98914	BBB+
NPRL 6124	0.99352	BBB+
NPRL 6125	0.97740	BBB+
NPRL 6126	0.94540	BBB+
NPRL 6127	0.96949	BBB+

NPRL 6128	0.98846	BBB+
NPRL 6129	0.80350	BBB+
NPRL 6130	0.87450	BBB+
NPRL 6131	0.99811	BBB+
NPRL 6132	0.95960	BBB+
NPRL 6133	0.90730	BBB+
NPRL 6134	0.86384	BBB+
NPRL 6135	0.96090	BBB+
NPRL 6136	0.99725	BBB+
NPRL 6137	0.67011	BBB+
NPRL 6138	0.69767	BBB+
NPRL 6139	0.97137	BBB+
NPRL 614	0.95216	BBB+
NPRL 6140	0.98750	BBB+
NPRL 6141	0.95261	BBB+
NPRL 6142	0.96980	BBB+
NPRL 6143	0.94043	BBB+
NPRL 6144	0.95424	BBB+
NPRL 6145	0.92179	BBB+
NPRL 6146	0.91727	BBB+
NPRL 6147	0.84449	BBB+
NPRL 6148	0.87443	BBB+
NPRL 6149	0.18910	BBB-
NPRL 6150	0.96935	BBB+
NPRL 6151	0.94401	BBB+
NPRL 6152	0.94767	BBB+
NPRL 6153	0.99675	BBB+
NPRL 6154	0.99967	BBB+
NPRL 6155	0.99885	BBB+
NPRL 6156	0.99858	BBB+
NPRL 6157	0.99342	BBB+
NPRL 6158	0.99598	BBB+
NPRL 6159	0.99921	BBB+
NPRL 6160	0.99061	BBB+
NPRL 6162	0.04530	BBB-
NPRL 6163	0.00448	BBB-
NPRL 6164	0.03933	BBB-
NPRL 6165	0.03613	BBB-
NPRL 6166	0.01228	BBB-
NPRL 6167	0.00149	BBB-
NPRL 6168	0.00959	BBB-
NPRL 6169	0.01039	BBB-
NPRL 6170	0.01555	BBB-
NPRL 6171	0.03042	BBB-
NPRL 6172	0.00530	BBB-
NPRL 6173	0.04078	BBB-
NPRL 6174	0.03874	BBB-
NPRL 6175	0.02910	BBB-
NPRL 6176	0.05801	BBB-
NPRL 6177	0.06085	BBB-
NPRL 6178	0.01855	BBB-
NPRL 6179	0.01780	BBB-
NPRL 6180	0.00711	BBB-
NPRL 6181	0.00918	BBB-
NPRL 6182	0.01398	BBB-
NPRL 6183	0.01404	BBB-
NPRL 6184	0.02622	BBB-

NPRL 6185	0.04245	BBB-
NPRL 6186	0.00879	BBB-
NPRL 6187	0.01424	BBB-
NPRL 6188	0.00888	BBB-
NPRL 6189	0.01369	BBB-
NPRL 6190	0.00081	BBB-
NPRL 6191	0.01164	BBB-
NPRL 6192	0.00244	BBB-
NPRL 6193	0.00822	BBB-
NPRL 6194	0.00709	BBB-
NPRL 6196	0.49600	BBB-
NPRL 6197	0.97206	BBB+
NPRL 6198	0.70129	BBB+
NPRL 6199	0.05900	BBB-
NPRL 62	0.02519	BBB-
NPRL 620	0.44151	BBB-
NPRL 6200	0.70305	BBB+
NPRL 6201	0.34672	BBB-
NPRL 6202	0.54283	BBB+
NPRL 6203	0.20819	BBB-
NPRL 6204	0.06910	BBB-
NPRL 6205	0.50350	BBB+
NPRL 6206	0.73094	BBB+
NPRL 6207	0.72909	BBB+
NPRL 6208	0.85919	BBB+
NPRL 6209	0.85587	BBB+
NPRL 6210	0.91665	BBB+
NPRL 6211	0.95550	BBB+
NPRL 6212	0.93951	BBB+
NPRL 6213	0.33433	BBB-
NPRL 6214	0.19563	BBB-
NPRL 6215	0.08268	BBB-
NPRL 6216	0.80737	BBB+
NPRL 6217	0.78390	BBB+
NPRL 6218	0.63284	BBB+
NPRL 622	0.99187	BBB+
NPRL 625	0.25815	BBB-
NPRL 626	0.82208	BBB+
NPRL 6266	0.57337	BBB+
NPRL 6267	0.03582	BBB-
NPRL 6268	0.09079	BBB-
NPRL 6269	0.38305	BBB-
NPRL 627	0.99125	BBB+
NPRL 6270	0.87025	BBB+
NPRL 6271	0.35209	BBB-
NPRL 6272	0.79814	BBB+
NPRL 6273	0.98885	BBB+
NPRL 6274	0.93742	BBB+
NPRL 6276	0.87699	BBB+
NPRL 6277	0.71446	BBB+
NPRL 6278	0.98202	BBB+
NPRL 6279	0.97035	BBB+
NPRL 6280	0.65378	BBB+
NPRL 6281	0.57796	BBB+
NPRL 6283	0.99890	BBB+
NPRL 6284	0.99826	BBB+
NPRL 6285	0.99222	BBB+

NPRL 6286	0.80984	BBB+
NPRL 6287	0.42695	BBB-
NPRL 6288	0.13815	BBB-
NPRL 6289	0.42417	BBB-
NPRL 6291	0.01013	BBB-
NPRL 6292	0.00068	BBB-
NPRL 6293	0.47661	BBB-
NPRL 6294	0.38800	BBB-
NPRL 6295	0.00022	BBB-
NPRL 6296	0.00041	BBB-
NPRL 6297	0.00032	BBB-
NPRL 6298	0.00041	BBB-
NPRL 6299	0.81424	BBB+
NPRL 630	0.90614	BBB+
NPRL 6300	0.73116	BBB+
NPRL 6302	0.88625	BBB+
NPRL 6303	0.76013	BBB+
NPRL 6304	0.97945	BBB+
NPRL 6305	0.97321	BBB+
NPRL 6306	0.00006	BBB-
NPRL 6307	0.00006	BBB-
NPRL 6309	0.72461	BBB+
NPRL 631	0.60716	BBB+
NPRL 6310	0.00628	BBB-
NPRL 6311	0.77693	BBB+
NPRL 6312	0.12465	BBB-
NPRL 6314	0.00633	BBB-
NPRL 6315	0.82329	BBB+
NPRL 6316	0.70193	BBB+
NPRL 6317	0.94328	BBB+
NPRL 6318	0.45448	BBB-
NPRL 6319	0.00435	BBB-
NPRL 632	0.86776	BBB+
NPRL 6320	0.98586	BBB+
NPRL 6321	0.64523	BBB+
NPRL 6322	0.08037	BBB-
NPRL 6323	0.00764	BBB-
NPRL 6324	0.00132	BBB-
NPRL 6325	0.00079	BBB-
NPRL 6326	0.04550	BBB-
NPRL 6327	0.01284	BBB-
NPRL 6328	0.00135	BBB-
NPRL 6329	0.00668	BBB-
NPRL 6330	0.00480	BBB-
NPRL 6331	0.19758	BBB-
NPRL 6332	0.06707	BBB-
NPRL 6333	0.37429	BBB-
NPRL 6334	0.19602	BBB-
NPRL 6336	0.18590	BBB-
NPRL 6337	0.04965	BBB-
NPRL 6338	0.00468	BBB-
NPRL 6339	0.00092	BBB-
NPRL 6340	0.00253	BBB-
NPRL 6341	0.00281	BBB-
NPRL 6342	0.00037	BBB-
NPRL 6343	0.02561	BBB-
NPRL 6345	0.61457	BBB+

NPRL 6347	0.98605	BBB+
NPRL 6348	0.86018	BBB+
NPRL 6349	0.00123	BBB-
NPRL 6350	0.07390	BBB-
NPRL 6351	0.23624	BBB-
NPRL 6352	0.01394	BBB-
NPRL 6353	0.07879	BBB-
NPRL 6354	0.22570	BBB-
NPRL 6355	0.16475	BBB-
NPRL 6356	0.51805	BBB+
NPRL 6357	0.03773	BBB-
NPRL 6358	0.99445	BBB+
NPRL 6359	0.94769	BBB+
NPRL 636	0.75740	BBB+
NPRL 6360	0.94840	BBB+
NPRL 6362	0.89191	BBB+
NPRL 6363	0.93261	BBB+
NPRL 6364	0.97180	BBB+
NPRL 6365	0.64914	BBB+
NPRL 6366	0.95897	BBB+
NPRL 6367	0.78305	BBB+
NPRL 6368	0.94992	BBB+
NPRL 6369	0.93360	BBB+
NPRL 6370	0.93896	BBB+
NPRL 6371	0.92914	BBB+
NPRL 6372	0.89783	BBB+
NPRL 6373	0.73448	BBB+
NPRL 6374	0.70663	BBB+
NPRL 6375	0.97405	BBB+
NPRL 6376	0.87801	BBB+
NPRL 6377	0.69613	BBB+
NPRL 6378	0.39451	BBB-
NPRL 6379	0.92207	BBB+
NPRL 6380	0.64468	BBB+
NPRL 6381	0.00660	BBB-
NPRL 6382	0.94825	BBB+
NPRL 6383	0.06883	BBB-
NPRL 6384	0.23500	BBB-
NPRL 6385	0.27303	BBB-
NPRL 6386	0.89028	BBB+
NPRL 6387	0.78977	BBB+
NPRL 6388	0.15729	BBB-
NPRL 6389	0.56779	BBB+
NPRL 6390	0.86086	BBB+
NPRL 6391	0.38124	BBB-
NPRL 6392	0.00143	BBB-
NPRL 6393	0.32590	BBB-
NPRL 6394	0.00124	BBB-
NPRL 6395	0.05746	BBB-
NPRL 6396	0.06243	BBB-
NPRL 6397	0.18351	BBB-
NPRL 6398	0.03631	BBB-
NPRL 6399	0.00982	BBB-
NPRL 64	0.00550	BBB-
NPRL 640	0.96624	BBB+
NPRL 6400	0.78975	BBB+
NPRL 6401	0.01115	BBB-

NPRL 6402	0.06259	BBB-
NPRL 6403	0.80772	BBB+
NPRL 6404	0.99678	BBB+
NPRL 6405	0.92160	BBB+
NPRL 6406	0.63767	BBB+
NPRL 6407	0.01621	BBB-
NPRL 6408	0.94718	BBB+
NPRL 6409	0.09052	BBB-
NPRL 641	0.46290	BBB-
NPRL 6410	0.09782	BBB-
NPRL 6411	0.49495	BBB-
NPRL 6412	0.23959	BBB-
NPRL 6413	0.55503	BBB+
NPRL 6414	0.99775	BBB+
NPRL 6415	0.99659	BBB+
NPRL 6416	0.71807	BBB+
NPRL 6417	0.86044	BBB+
NPRL 6419	0.92252	BBB+
NPRL 642	0.96170	BBB+
NPRL 6420	0.92706	BBB+
NPRL 6421	0.52280	BBB+
NPRL 6422	0.11670	BBB-
NPRL 6423	0.24844	BBB-
NPRL 6424	0.11420	BBB-
NPRL 6425	0.19302	BBB-
NPRL 6426	0.72740	BBB+
NPRL 6427	0.55778	BBB+
NPRL 6428	0.88380	BBB+
NPRL 6429	0.33275	BBB-
NPRL 6430	0.86560	BBB+
NPRL 6431	0.64989	BBB+
NPRL 6432	0.39297	BBB-
NPRL 6433	0.97302	BBB+
NPRL 6434	0.06243	BBB-
NPRL 6435	0.36458	BBB-
NPRL 6436	0.92121	BBB+
NPRL 6437	0.99196	BBB+
NPRL 6438	0.98868	BBB+
NPRL 6439	0.88147	BBB+
NPRL 644	0.98197	BBB+
NPRL 6440	0.88061	BBB+
NPRL 6441	0.92168	BBB+
NPRL 6442	0.72543	BBB+
NPRL 6443	0.81855	BBB+
NPRL 6444	0.86073	BBB+
NPRL 6445	0.57193	BBB+
NPRL 6446	0.89184	BBB+
NPRL 6447	0.90650	BBB+
NPRL 6448	0.93608	BBB+
NPRL 6449	0.85018	BBB+
NPRL 6450	0.67395	BBB+
NPRL 6451	0.84151	BBB+
NPRL 6452	0.73517	BBB+
NPRL 6453	0.21883	BBB-
NPRL 6454	0.80327	BBB+
NPRL 6455	0.64575	BBB+
NPRL 6456	0.09483	BBB-

NPRL 6457	0.52363	BBB+
NPRL 6458	0.98312	BBB+
NPRL 6459	0.82267	BBB+
NPRL 6460	0.47753	BBB-
NPRL 6461	0.55745	BBB+
NPRL 6463	0.25143	BBB-
NPRL 6464	0.89551	BBB+
NPRL 6465	0.85573	BBB+
NPRL 6466	0.63410	BBB+
NPRL 6467	0.77220	BBB+
NPRL 6468	0.98403	BBB+
NPRL 6469	0.77105	BBB+
NPRL 6470	0.89121	BBB+
NPRL 6471	0.88181	BBB+
NPRL 6472	0.98567	BBB+
NPRL 6473	0.66035	BBB+
NPRL 6474	0.71529	BBB+
NPRL 6486	0.99288	BBB+
NPRL 6487	0.70031	BBB+
NPRL 6488	0.99003	BBB+
NPRL 6489	0.63657	BBB+
NPRL 6490	0.81756	BBB+
NPRL 6491	0.67188	BBB+
NPRL 6492	0.85966	BBB+
NPRL 6493	0.60718	BBB+
NPRL 6494	0.77017	BBB+
NPRL 6495	0.14691	BBB-
NPRL 6496	0.87192	BBB+
NPRL 6497	0.25301	BBB-
NPRL 6498	0.88124	BBB+
NPRL 6499	0.96223	BBB+
NPRL 6500	0.98057	BBB+
NPRL 6501	0.98677	BBB+
NPRL 6502	0.14108	BBB-
NPRL 6503	0.55335	BBB+
NPRL 6504	0.17468	BBB-
NPRL 6511	0.52799	BBB+
NPRL 6512	0.62044	BBB+
NPRL 6513	0.35545	BBB-
NPRL 6514	0.01835	BBB-
NPRL 6515	0.98467	BBB+
NPRL 6516	0.98954	BBB+
NPRL 6517	0.92595	BBB+
NPRL 6518	0.72816	BBB+
NPRL 6519	0.89229	BBB+
NPRL 6520	0.90638	BBB+
NPRL 6521	0.56804	BBB+
NPRL 6522	0.95816	BBB+
NPRL 6523	0.92237	BBB+
NPRL 6524	0.98709	BBB+
NPRL 6525	0.12830	BBB-
NPRL 6526	0.90453	BBB+
NPRL 6527	0.75715	BBB+
NPRL 6528	0.99923	BBB+
NPRL 6529	0.43284	BBB-
NPRL 653	0.99372	BBB+
NPRL 6530	0.50284	BBB+

NPRL 6531	0.92777	BBB+
NPRL 6532	0.76781	BBB+
NPRL 6533	0.14704	BBB-
NPRL 6534	0.16221	BBB-
NPRL 6535	0.63615	BBB+
NPRL 6536	0.96084	BBB+
NPRL 6537	0.92210	BBB+
NPRL 6538	0.69603	BBB+
NPRL 6539	0.99122	BBB+
NPRL 6540	0.81724	BBB+
NPRL 6541	0.96583	BBB+
NPRL 6542	0.82740	BBB+
NPRL 6543	0.82942	BBB+
NPRL 6544	0.31121	BBB-
NPRL 6545	0.94574	BBB+
NPRL 6546	0.70772	BBB+
NPRL 6547	0.87603	BBB+
NPRL 6548	0.60117	BBB+
NPRL 6549	0.89286	BBB+
NPRL 6553	0.01711	BBB-
NPRL 6554	0.00847	BBB-
NPRL 6555	0.93396	BBB+
NPRL 6556	0.00388	BBB-
NPRL 6557	0.92541	BBB+
NPRL 6558	0.66165	BBB+
NPRL 6559	0.65618	BBB+
NPRL 656	0.19896	BBB-
NPRL 6560	0.52873	BBB+
NPRL 6561	0.52499	BBB+
NPRL 6562	0.01697	BBB-
NPRL 6563	0.97460	BBB+
NPRL 6567	0.97437	BBB+
NPRL 6568	0.86778	BBB+
NPRL 6569	0.99862	BBB+
NPRL 657	0.90015	BBB+
NPRL 6571	0.78975	BBB+
NPRL 6572	0.94913	BBB+
NPRL 6573	0.87491	BBB+
NPRL 6574	0.86133	BBB+
NPRL 6575	0.70980	BBB+
NPRL 658	0.86156	BBB+
NPRL 6585	0.06019	BBB-
NPRL 6586	0.07174	BBB-
NPRL 6587	0.01573	BBB-
NPRL 6588	0.00068	BBB-
NPRL 6589	0.82528	BBB+
NPRL 659	0.74572	BBB+
NPRL 66	0.42862	BBB-
NPRL 660	0.43643	BBB-
NPRL 661	0.96423	BBB+
NPRL 662	0.71168	BBB+
NPRL 663	0.95448	BBB+
NPRL 664	0.74560	BBB+
NPRL 665	0.99973	BBB+
NPRL 667	0.95050	BBB+
NPRL 669	0.97498	BBB+
NPRL 67	0.97987	BBB+

NPRL 670	0.92492	BBB+
NPRL 671	0.55757	BBB+
NPRL 675	0.89907	BBB+
NPRL 676	0.90120	BBB+
NPRL 677	0.95284	BBB+
NPRL 679	0.90489	BBB+
NPRL 68	0.18806	BBB-
NPRL 680	0.91965	BBB+
NPRL 682	0.95535	BBB+
NPRL 684	0.97613	BBB+
NPRL 685	0.96437	BBB+
NPRL 686	0.41527	BBB-
NPRL 688	0.42443	BBB-
NPRL 689	0.51969	BBB+
NPRL 69	0.78820	BBB+
NPRL 690	0.58025	BBB+
NPRL 691	0.84120	BBB+
NPRL 692	0.66610	BBB+
NPRL 693	0.55252	BBB+
NPRL 694	0.34317	BBB-
NPRL 695	0.71551	BBB+
NPRL 698	0.77869	BBB+
NPRL 7	0.96395	BBB+
NPRL 70	0.95143	BBB+
NPRL 700	0.82284	BBB+
NPRL 702	0.08128	BBB-
NPRL 703	0.99225	BBB+
NPRL 704	0.80018	BBB+
NPRL 707	0.98679	BBB+
NPRL 71	0.12443	BBB-
NPRL 72	0.98842	BBB+
NPRL 720	0.58961	BBB+
NPRL 723	0.94272	BBB+
NPRL 724	0.01967	BBB-
NPRL 725	0.03401	BBB-
NPRL 726	0.01031	BBB-
NPRL 727	0.01797	BBB-
NPRL 728	0.05503	BBB-
NPRL 729	0.10337	BBB-
NPRL 73	0.97144	BBB+
NPRL 730	0.10941	BBB-
NPRL 731	0.01747	BBB-
NPRL 732	0.03271	BBB-
NPRL 733	0.00237	BBB-
NPRL 734	0.09378	BBB-
NPRL 736	0.03210	BBB-
NPRL 737	0.10902	BBB-
NPRL 738	0.07600	BBB-
NPRL 739	0.04755	BBB-
NPRL 74	0.91477	BBB+
NPRL 740	0.09973	BBB-
NPRL 741	0.00201	BBB-
NPRL 742	0.01944	BBB-
NPRL 743	0.02518	BBB-
NPRL 744	0.01879	BBB-
NPRL 745	0.10316	BBB-
NPRL 746	0.05289	BBB-

NPRL 747	0.98793	BBB+
NPRL 748	0.13040	BBB-
NPRL 75	0.59050	BBB+
NPRL 751	0.41952	BBB-
NPRL 752	0.10579	BBB-
NPRL 753	0.88396	BBB+
NPRL 754	0.23299	BBB-
NPRL 755	0.91572	BBB+
NPRL 756	0.04363	BBB-
NPRL 757	0.42893	BBB-
NPRL 758	0.54158	BBB+
NPRL 759	0.64358	BBB+
NPRL 76	0.98463	BBB+
NPRL 760	0.11311	BBB-
NPRL 762	0.98674	BBB+
NPRL 763	0.91188	BBB+
NPRL 764	0.95595	BBB+
NPRL 765	0.66515	BBB+
NPRL 766	0.60728	BBB+
NPRL 767	0.84704	BBB+
NPRL 768	0.38620	BBB-
NPRL 769	0.53144	BBB+
NPRL 77	0.14228	BBB-
NPRL 770	0.98778	BBB+
NPRL 771	0.86314	BBB+
NPRL 772	0.97580	BBB+
NPRL 773	0.74348	BBB+
NPRL 774	0.86126	BBB+
NPRL 775	0.97938	BBB+
NPRL 776	0.78735	BBB+
NPRL 777	0.78023	BBB+
NPRL 778	0.39870	BBB-
NPRL 779	0.62232	BBB+
NPRL 78	0.42539	BBB-
NPRL 780	0.63185	BBB+
NPRL 781	0.40311	BBB-
NPRL 783	0.75049	BBB+
NPRL 784	0.96435	BBB+
NPRL 785	0.10606	BBB-
NPRL 786	0.54141	BBB+
NPRL 787	0.01746	BBB-
NPRL 788	0.73250	BBB+
NPRL 789	0.62937	BBB+
NPRL 79	0.46358	BBB-
NPRL 791	0.24231	BBB-
NPRL 792	0.05680	BBB-
NPRL 793	0.96249	BBB+
NPRL 794	0.89994	BBB+
NPRL 795	0.02379	BBB-
NPRL 796	0.87027	BBB+
NPRL 797	0.03370	BBB-
NPRL 799	0.02771	BBB-
NPRL 8	0.63254	BBB+
NPRL 80	0.32961	BBB-
NPRL 801	0.03655	BBB-
NPRL 802	0.72222	BBB+
NPRL 803	0.90938	BBB+

NPRL 804	0.04533	BBB-
NPRL 805	0.03809	BBB-
NPRL 806	0.92527	BBB+
NPRL 807	0.00032	BBB-
NPRL 808	0.79971	BBB+
NPRL 809	0.00977	BBB-
NPRL 811	0.06711	BBB-
NPRL 814	0.11015	BBB-
NPRL 815	0.99629	BBB+
NPRL 816	0.96095	BBB+
NPRL 817	0.92930	BBB+
NPRL 818	0.99886	BBB+
NPRL 819	0.98123	BBB+
NPRL 82	0.96210	BBB+
NPRL 820	0.99892	BBB+
NPRL 821	0.78462	BBB+
NPRL 822	0.52142	BBB+
NPRL 823	0.21742	BBB-
NPRL 824	0.67401	BBB+
NPRL 825	0.82097	BBB+
NPRL 826	0.92848	BBB+
NPRL 827	0.82702	BBB+
NPRL 828	0.99455	BBB+
NPRL 829	0.42581	BBB-
NPRL 830	0.91061	BBB+
NPRL 831	0.95677	BBB+
NPRL 832	0.73435	BBB+
NPRL 833	0.99891	BBB+
NPRL 834	0.98965	BBB+
NPRL 835	0.99959	BBB+
NPRL 836	0.99553	BBB+
NPRL 837	0.95727	BBB+
NPRL 838	0.97689	BBB+
NPRL 839	0.99610	BBB+
NPRL 840	0.74353	BBB+
NPRL 841	0.98627	BBB+
NPRL 842	0.99637	BBB+
NPRL 843	0.80184	BBB+
NPRL 844	0.84706	BBB+
NPRL 845	0.88430	BBB+
NPRL 846	0.58077	BBB+
NPRL 847	0.83733	BBB+
NPRL 848	0.00271	BBB-
NPRL 849	0.00130	BBB-
NPRL 85	0.22174	BBB-
NPRL 850	0.00820	BBB-
NPRL 851	0.35596	BBB-
NPRL 853	0.51897	BBB+
NPRL 854	0.99250	BBB+
NPRL 855	0.99647	BBB+
NPRL 856	0.98557	BBB+
NPRL 857	0.05889	BBB-
NPRL 859	0.21515	BBB-
NPRL 860	0.85022	BBB+
NPRL 861	0.62612	BBB+
NPRL 862	0.31818	BBB-
NPRL 863	0.28881	BBB-

NPRL 864	0.97219	BBB+
NPRL 865	0.98573	BBB+
NPRL 866	0.97120	BBB+
NPRL 867	0.99307	BBB+
NPRL 868	0.81801	BBB+
NPRL 869	0.94434	BBB+
NPRL 87	0.89297	BBB+
NPRL 870	0.90658	BBB+
NPRL 871	0.52002	BBB+
NPRL 872	0.99464	BBB+
NPRL 873	0.92762	BBB+
NPRL 874	0.99315	BBB+
NPRL 875	0.58603	BBB+
NPRL 876	0.58044	BBB+
NPRL 877	0.91218	BBB+
NPRL 88	0.97093	BBB+
NPRL 89	0.85746	BBB+
NPRL 9	0.98110	BBB+
NPRL 91	0.87978	BBB+
NPRL 95	0.39881	BBB-
NPRL 96	0.95330	BBB+
NPRL 97	0.19011	BBB-
NPRL 98	0.47031	BBB-
NPRL 99	0.99135	BBB+
NPRL1000	0.99205	BBB+
NPRL1001	0.02501	BBB-
NPRL1002	0.00339	BBB-
NPRL1003	0.07381	BBB-
NPRL1004	0.21468	BBB-
NPRL1005	0.92094	BBB+
NPRL1006	0.00523	BBB-
NPRL1007	0.00242	BBB-
NPRL1008	0.03609	BBB-
NPRL1009	0.97744	BBB+
NPRL1010	0.00366	BBB-
NPRL1011	0.00439	BBB-
NPRL1012	0.98796	BBB+
NPRL1013	0.95478	BBB+
NPRL1014	0.00647	BBB-
NPRL1015	0.00144	BBB-
NPRL1016	0.00837	BBB-
NPRL1017	0.98565	BBB+
NPRL1018	0.34240	BBB-
NPRL1019	0.86697	BBB+
NPRL1020	0.88369	BBB+
NPRL1021	0.53153	BBB+
NPRL1022	0.98892	BBB+
NPRL1023	0.92729	BBB+
NPRL1024	0.96803	BBB+
NPRL1026	0.00896	BBB-
NPRL1027	0.99285	BBB+
NPRL1028	0.00138	BBB-
NPRL1029	0.99813	BBB+
NPRL1030	0.00472	BBB-
NPRL1031	0.00580	BBB-
NPRL1032	0.04151	BBB-
NPRL1033	0.00510	BBB-

NPRL1034	0.00398	BBB-
NPRL1035	0.01921	BBB-
NPRL1036	0.01592	BBB-
NPRL1037	0.00101	BBB-
NPRL1038	0.01173	BBB-
NPRL1039	0.00202	BBB-
NPRL1040	0.82171	BBB+
NPRL1041	0.24051	BBB-
NPRL1042	0.97124	BBB+
NPRL1043	0.80158	BBB+
NPRL1044	0.05362	BBB-
NPRL1045	0.00891	BBB-
NPRL1046	0.07905	BBB-
NPRL1047	0.90314	BBB+
NPRL1052	0.73315	BBB+
NPRL1055	0.02941	BBB-
NPRL1056	0.00096	BBB-
NPRL1058	0.87780	BBB+
NPRL1059	0.97394	BBB+
NPRL1060	0.26941	BBB-
NPRL1061	0.11576	BBB-
NPRL1062	0.28526	BBB-
NPRL1067	0.94537	BBB+
NPRL1068	0.69431	BBB+
NPRL1069	0.52963	BBB+
NPRL1071	0.74057	BBB+
NPRL1072	0.96773	BBB+
NPRL1073	0.05071	BBB-
NPRL1076	0.99053	BBB+
NPRL1083	0.45814	BBB-
NPRL1084	0.40052	BBB-
NPRL1085	0.00184	BBB-
NPRL1086	0.96743	BBB+
NPRL1087	0.82605	BBB+
NPRL1088	0.00493	BBB-
NPRL1089	0.99921	BBB+
NPRL1090	0.99704	BBB+
NPRL1092	0.90469	BBB+
NPRL1093	0.98444	BBB+
NPRL1094	0.01857	BBB-
NPRL1096	0.73585	BBB+
NPRL1098	0.77756	BBB+
NPRL1099	0.36928	BBB-
NPRL1101	0.69443	BBB+
NPRL1103	0.87540	BBB+
NPRL1104	0.53069	BBB+
NPRL1105	0.41243	BBB-
NPRL1106	0.90012	BBB+
NPRL1107	0.74640	BBB+
NPRL1109	0.00252	BBB-
NPRL1110	0.93114	BBB+
NPRL1111	0.66294	BBB+
NPRL1112	0.00533	BBB-
NPRL1113	0.00412	BBB-
NPRL1114	0.99635	BBB+
NPRL1115	0.88191	BBB+
NPRL1116	0.90661	BBB+

NPRL1118	0.92321	BBB+
NPRL1119	0.98120	BBB+
NPRL1120	0.94468	BBB+
NPRL1121	0.85535	BBB+
NPRL1122	0.83085	BBB+
NPRL1124	0.95159	BBB+
NPRL1125	0.88880	BBB+
NPRL1127	0.90654	BBB+
NPRL1128	0.83918	BBB+
NPRL1129	0.91268	BBB+
NPRL1130	0.71323	BBB+
NPRL1131	0.92931	BBB+
NPRL1132	0.00067	BBB-
NPRL1133	0.91636	BBB+
NPRL1134	0.72366	BBB+
NPRL1135	0.81118	BBB+
NPRL1136	0.74748	BBB+
NPRL1137	0.96419	BBB+
NPRL1142	0.91284	BBB+
NPRL1143	0.06561	BBB-
NPRL1144	0.91215	BBB+
NPRL1145	0.83182	BBB+
NPRL1146	0.30954	BBB-
NPRL1147	0.67583	BBB+
NPRL1149	0.88456	BBB+
NPRL1150	0.86176	BBB+
NPRL1151	0.68517	BBB+
NPRL1152	0.70880	BBB+
NPRL1153	0.38114	BBB-
NPRL1157	0.54708	BBB+
NPRL1159	0.77672	BBB+
NPRL1160	0.86777	BBB+
NPRL1161	0.80768	BBB+
NPRL1162	0.92632	BBB+
NPRL1163	0.22877	BBB-
NPRL1165	0.83152	BBB+
NPRL1168	0.53828	BBB+
NPRL1169	0.83055	BBB+
NPRL1171	0.28044	BBB-
NPRL1173	0.70641	BBB+
NPRL1175	0.50480	BBB+
NPRL1176	0.96905	BBB+
NPRL1177	0.96518	BBB+
NPRL1178	0.96689	BBB+
NPRL1179	0.75674	BBB+
NPRL1180	0.99334	BBB+
NPRL1181	0.99158	BBB+
NPRL1182	0.98789	BBB+
NPRL1183	0.98018	BBB+
NPRL1184	0.81023	BBB+
NPRL1185	0.99958	BBB+
NPRL1186	0.99789	BBB+
NPRL1187	0.85615	BBB+
NPRL1188	0.99738	BBB+
NPRL1189	0.99062	BBB+
NPRL1190	0.99308	BBB+
NPRL1191	0.97403	BBB+

NPRL1192	0.99232	BBB+
NPRL1193	0.99185	BBB+
NPRL1194	0.94135	BBB+
NPRL1195	0.99897	BBB+
NPRL1196	0.99057	BBB+
NPRL1197	0.93861	BBB+
NPRL1198	0.61676	BBB+
NPRL1199	0.98061	BBB+
NPRL1200	0.92886	BBB+
NPRL1201	0.94887	BBB+
NPRL1202	0.94941	BBB+
NPRL1203	0.96705	BBB+
NPRL1204	0.47390	BBB-
NPRL1205	0.95406	BBB+
NPRL1207	0.91485	BBB+
NPRL1208	0.34167	BBB-
NPRL1209	0.97234	BBB+
NPRL1210	0.94643	BBB+
NPRL1211	0.97725	BBB+
NPRL1212	0.75503	BBB+
NPRL1213	0.97527	BBB+
NPRL1214	0.98623	BBB+
NPRL1215	0.21503	BBB-
NPRL1216	0.92463	BBB+
NPRL1217	0.25814	BBB-
NPRL1218	0.97211	BBB+
NPRL1222	0.96651	BBB+
NPRL1223	0.99394	BBB+
NPRL1224	0.90447	BBB+
NPRL1225	0.84817	BBB+
NPRL1226	0.93390	BBB+
NPRL1228	0.47829	BBB-
NPRL1229	0.76117	BBB+
NPRL1232	0.90689	BBB+
NPRL1235	0.98701	BBB+
NPRL1237	0.33356	BBB-
NPRL1238	0.94945	BBB+
NPRL1239	0.99645	BBB+
NPRL1240	0.98162	BBB+
NPRL1241	0.99599	BBB+
NPRL1242	0.97461	BBB+
NPRL1243	0.99100	BBB+
NPRL1244	0.99741	BBB+
NPRL1245	0.30118	BBB-
NPRL1247	0.95724	BBB+
NPRL1250	0.86318	BBB+
NPRL1251	0.99853	BBB+
NPRL1252	0.99863	BBB+
NPRL1253	0.42480	BBB-
NPRL1254	0.95699	BBB+
NPRL1255	0.97432	BBB+
NPRL1259	0.96084	BBB+
NPRL1260	0.59242	BBB+
NPRL1261	0.87608	BBB+
NPRL1262	0.87206	BBB+
NPRL1263	0.97567	BBB+
NPRL1264	0.96762	BBB+

NPRL1265	0.10661	BBB-
NPRL1266	0.18441	BBB-
NPRL1267	0.16089	BBB-
NPRL1268	0.74246	BBB+
NPRL1270	0.27552	BBB-
NPRL1271	0.51298	BBB+
NPRL1272	0.83107	BBB+
NPRL1273	0.85799	BBB+
NPRL1275	0.86132	BBB+
NPRL1277	0.58319	BBB+
NPRL1278	0.20165	BBB-
NPRL1280	0.56755	BBB+
NPRL1281	0.28449	BBB-
NPRL1282	0.02786	BBB-
NPRL1284	0.04319	BBB-
NPRL1285	0.01935	BBB-
NPRL1286	0.20250	BBB-
NPRL1287	0.72378	BBB+
NPRL1288	0.88251	BBB+
NPRL1289	0.91489	BBB+
NPRL1290	0.08748	BBB-
NPRL1292	0.93426	BBB+
NPRL1294	0.76440	BBB+
NPRL1295	0.64928	BBB+
NPRL1296	0.08439	BBB-
NPRL1297	0.07110	BBB-
NPRL1299	0.99201	BBB+
NPRL1300	0.99249	BBB+
NPRL1301	0.97329	BBB+
NPRL1302	0.35672	BBB-
NPRL1303	0.94540	BBB+
NPRL1305	0.37709	BBB-
NPRL1306	0.89450	BBB+
NPRL1307	0.89445	BBB+
NPRL1309	0.97202	BBB+
NPRL1310	0.93855	BBB+
NPRL1311	0.68064	BBB+
NPRL1312	0.28251	BBB-
NPRL1313	0.98569	BBB+
NPRL1314	0.69732	BBB+
NPRL1315	0.92094	BBB+
NPRL1316	0.52896	BBB+
NPRL1317	0.68590	BBB+
NPRL1320	0.14953	BBB-
NPRL1322	0.68722	BBB+
NPRL1323	0.26020	BBB-
NPRL1325	0.09975	BBB-
NPRL1326	0.94016	BBB+
NPRL1327	0.44982	BBB-
NPRL1328	0.60054	BBB+
NPRL1329	0.34718	BBB-
NPRL1330	0.66135	BBB+
NPRL1331	0.76007	BBB+
NPRL1334	0.67964	BBB+
NPRL1335	0.98290	BBB+
NPRL1336	0.98863	BBB+
NPRL1337	0.68695	BBB+

NPRL1339	0.78362	BBB+
NPRL1340	0.67355	BBB+
NPRL1341	0.63432	BBB+
NPRL1342	0.66318	BBB+
NPRL1343	0.59732	BBB+
NPRL1344	0.55805	BBB+
NPRL1346	0.95703	BBB+
NPRL1347	0.15278	BBB-
NPRL1348	0.29579	BBB-
NPRL1349	0.37376	BBB-
NPRL1350	0.21295	BBB-
NPRL1351	0.49913	BBB-
NPRL1352	0.68262	BBB+
NPRL1353	0.64855	BBB+
NPRL1354	0.25881	BBB-
NPRL1355	0.98047	BBB+
NPRL1356	0.82138	BBB+
NPRL1357	0.97047	BBB+
NPRL1358	0.99054	BBB+
NPRL1359	0.95452	BBB+
NPRL1360	0.34489	BBB-
NPRL1361	0.31063	BBB-
NPRL1362	0.98083	BBB+
NPRL1363	0.73332	BBB+
NPRL1364	0.21942	BBB-
NPRL1365	0.13690	BBB-
NPRL1366	0.53626	BBB+
NPRL1367	0.97273	BBB+
NPRL1368	0.80756	BBB+
NPRL1369	0.48965	BBB-
NPRL1370	0.59241	BBB+
NPRL1371	0.50793	BBB+
NPRL1372	0.93603	BBB+
NPRL1373	0.94682	BBB+
NPRL1374	0.34934	BBB-
NPRL1375	0.53500	BBB+
NPRL1377	0.91530	BBB+
NPRL1378	0.91376	BBB+
NPRL1379	0.20332	BBB-
NPRL1381	0.23427	BBB-
NPRL1382	0.01477	BBB-
NPRL1383	0.91091	BBB+
NPRL1384	0.76915	BBB+
NPRL1386	0.67533	BBB+
NPRL1388	0.11922	BBB-
NPRL1391	0.97442	BBB+
NPRL1392	0.95706	BBB+
NPRL1393	0.96202	BBB+
NPRL1394	0.85569	BBB+
NPRL1396	0.98688	BBB+
NPRL1397	0.98583	BBB+
NPRL1398	0.76804	BBB+
NPRL1399	0.94980	BBB+
NPRL1400	0.91561	BBB+
NPRL1401	0.03562	BBB-
NPRL1402	0.44861	BBB-
NPRL1404	0.62977	BBB+

NPRL1405	0.99699	BBB+
NPRL1406	0.53469	BBB+
NPRL1407	0.12443	BBB-
NPRL1408	0.08867	BBB-
NPRL1409	0.96114	BBB+
NPRL1410	0.95902	BBB+
NPRL1411	0.18238	BBB-
NPRL1412	0.96857	BBB+
NPRL1415	0.94522	BBB+
NPRL1416	0.02897	BBB-
NPRL1417	0.90825	BBB+
NPRL1419	0.96201	BBB+
NPRL1420	0.84907	BBB+
NPRL1421	0.93695	BBB+
NPRL1422	0.89335	BBB+
NPRL1423	0.49062	BBB-
NPRL1424	0.00202	BBB-
NPRL1425	0.06800	BBB-
NPRL1426	0.03222	BBB-
NPRL1427	0.10821	BBB-
NPRL1428	0.04495	BBB-
NPRL1429	0.01772	BBB-
NPRL1430	0.02495	BBB-
NPRL1431	0.00019	BBB-
NPRL1432	0.00064	BBB-
NPRL1433	0.00026	BBB-
NPRL1434	0.00079	BBB-
NPRL1435	0.91649	BBB+
NPRL1436	0.58634	BBB+
NPRL1437	0.02984	BBB-
NPRL1438	0.80634	BBB+
NPRL1439	0.96953	BBB+
NPRL1440	0.53983	BBB+
NPRL1443	0.70202	BBB+
NPRL1444	0.15751	BBB-
NPRL1445	0.71324	BBB+
NPRL1446	0.58864	BBB+
NPRL1447	0.80173	BBB+
NPRL1448	0.91102	BBB+
NPRL1449	0.99203	BBB+
NPRL1450	0.00228	BBB-
NPRL1451	0.32478	BBB-
NPRL1452	0.41389	BBB-
NPRL1453	0.95391	BBB+
NPRL1454	0.01501	BBB-
NPRL1455	0.69783	BBB+
NPRL1456	0.73142	BBB+
NPRL1457	0.84649	BBB+
NPRL1458	0.85683	BBB+
NPRL1460	0.96098	BBB+
NPRL1462	0.99175	BBB+
NPRL1463	0.92656	BBB+
NPRL1464	0.81182	BBB+
NPRL1465	0.32411	BBB-
NPRL1466	0.00073	BBB-
NPRL1468	0.96892	BBB+
NPRL1469	0.25777	BBB-

NPRL1470	0.08633	BBB-
NPRL1471	0.99574	BBB+
NPRL1472	0.85446	BBB+
NPRL1473	0.40478	BBB-
NPRL1474	0.95412	BBB+
NPRL1475	0.49885	BBB-
NPRL1476	0.66447	BBB+
NPRL1477	0.99843	BBB+
NPRL1478	0.99849	BBB+
NPRL1480	0.89807	BBB+
NPRL1481	0.92637	BBB+
NPRL1482	0.09876	BBB-
NPRL1483	0.93752	BBB+
NPRL1484	0.00144	BBB-
NPRL1485	0.11236	BBB-
NPRL1486	0.15501	BBB-
NPRL1487	0.00151	BBB-
NPRL1488	0.00080	BBB-
NPRL1489	0.00056	BBB-
NPRL1490	0.33173	BBB-
NPRL1491	0.98368	BBB+
NPRL1492	0.94228	BBB+
NPRL1493	0.18411	BBB-
NPRL1494	0.31710	BBB-
NPRL1495	0.09028	BBB-
NPRL1496	0.34590	BBB-
NPRL1497	0.30635	BBB-
NPRL1498	0.02257	BBB-
NPRL1499	0.05224	BBB-
NPRL1500	0.99502	BBB+
NPRL1502	0.09261	BBB-
NPRL1503	0.00133	BBB-
NPRL1505	0.31684	BBB-
NPRL1506	0.22786	BBB-
NPRL1507	0.07085	BBB-
NPRL1508	0.94246	BBB+
NPRL1509	0.92996	BBB+
NPRL1510	0.06664	BBB-
NPRL1511	0.99202	BBB+
NPRL1512	0.97142	BBB+
NPRL1513	0.92024	BBB+
NPRL1514	0.50252	BBB+
NPRL1515	0.85918	BBB+
NPRL1516	0.98414	BBB+
NPRL1517	0.00851	BBB-
NPRL1518	0.00979	BBB-
NPRL1519	0.99556	BBB+
NPRL1520	0.84554	BBB+
NPRL1521	0.97024	BBB+
NPRL1522	0.76382	BBB+
NPRL1523	0.98483	BBB+
NPRL1524	0.71389	BBB+
NPRL1525	0.97966	BBB+
NPRL1526	0.88140	BBB+
NPRL1528	0.02585	BBB-
NPRL1530	0.87046	BBB+
NPRL1531	0.53689	BBB+

NPRL1532	0.96190	BBB+
NPRL1533	0.43967	BBB-
NPRL1535	0.24790	BBB-
NPRL1536	0.71337	BBB+
NPRL1537	0.96879	BBB+
NPRL1538	0.96951	BBB+
NPRL1539	0.96484	BBB+
NPRL1540	0.82549	BBB+
NPRL1541	0.83041	BBB+
NPRL1542	0.98874	BBB+
NPRL1543	0.45556	BBB-
NPRL1544	0.90280	BBB+
NPRL1545	0.02280	BBB-
NPRL1546	0.70187	BBB+
NPRL1547	0.52462	BBB+
NPRL1550	0.94374	BBB+
NPRL1551	0.67305	BBB+
NPRL1552	0.66344	BBB+
NPRL1553	0.38025	BBB-
NPRL1555	0.78875	BBB+
NPRL1556	0.80449	BBB+
NPRL1557	0.88610	BBB+
NPRL1558	0.00356	BBB-
NPRL1559	0.99014	BBB+
NPRL1560	0.39366	BBB-
NPRL1561	0.57662	BBB+
NPRL1562	0.30970	BBB-
NPRL1563	0.40354	BBB-
NPRL1564	0.93245	BBB+
NPRL1565	0.49372	BBB-
NPRL1566	0.05246	BBB-
NPRL1567	0.89437	BBB+
NPRL1568	0.58817	BBB+
NPRL1569	0.19733	BBB-
NPRL1570	0.77928	BBB+
NPRL1571	0.06880	BBB-
NPRL1572	0.78554	BBB+
NPRL1573	0.99153	BBB+
NPRL1574	0.33805	BBB-
NPRL1577	0.09283	BBB-
NPRL1578	0.90797	BBB+
NPRL1579	0.07231	BBB-
NPRL1580	0.09666	BBB-
NPRL1582	0.31891	BBB-
NPRL1583	0.36996	BBB-
NPRL1586	0.73332	BBB+
NPRL1589	0.81355	BBB+
NPRL1590	0.21660	BBB-
NPRL1593	0.48680	BBB-
NPRL1594	0.32372	BBB-
NPRL1595	0.79531	BBB+
NPRL1596	0.01404	BBB-
NPRL1597	0.65193	BBB+
NPRL1598	0.33717	BBB-
NPRL1599	0.97611	BBB+
NPRL1601	0.96521	BBB+
NPRL1602	0.81391	BBB+

NPRL1603	0.01170	BBB-
NPRL1604	0.44816	BBB-
NPRL1606	0.61063	BBB+
NPRL1607	0.84461	BBB+
NPRL1609	0.14734	BBB-
NPRL1614	0.91533	BBB+
NPRL1616	0.94433	BBB+
NPRL1618	0.35597	BBB-
NPRL1620	0.71284	BBB+
NPRL1623	0.21470	BBB-
NPRL1625	0.60014	BBB+
NPRL1627	0.04892	BBB-
NPRL1628	0.16366	BBB-
NPRL1629	0.79716	BBB+
NPRL1630	0.05165	BBB-
NPRL1633	0.89241	BBB+
NPRL1636	0.68620	BBB+
NPRL1637	0.05751	BBB-
NPRL1639	0.09765	BBB-
NPRL1640	0.76155	BBB+
NPRL1641	0.06110	BBB-
NPRL1642	0.07846	BBB-
NPRL1643	0.52085	BBB+
NPRL1644	0.77020	BBB+
NPRL1646	0.75853	BBB+
NPRL1649	0.94504	BBB+
NPRL1651	0.83577	BBB+
NPRL1653	0.82614	BBB+
NPRL1657	0.70495	BBB+
NPRL1658	0.82269	BBB+
NPRL1659	0.60567	BBB+
NPRL1660	0.36843	BBB-
NPRL1661	0.07090	BBB-
NPRL1662	0.06422	BBB-
NPRL1663	0.20722	BBB-
NPRL1664	0.35589	BBB-
NPRL1665	0.63208	BBB+
NPRL1666	0.98351	BBB+
NPRL1667	0.68837	BBB+
NPRL1668	0.93408	BBB+
NPRL1669	0.48150	BBB-
NPRL1670	0.23653	BBB-
NPRL1671	0.33629	BBB-
NPRL1672	0.16344	BBB-
NPRL1673	0.14767	BBB-
NPRL1674	0.03223	BBB-
NPRL1675	0.09728	BBB-
NPRL1676	0.13105	BBB-
NPRL1677	0.08539	BBB-
NPRL1678	0.47747	BBB-
NPRL1679	0.45755	BBB-
NPRL1680	0.34027	BBB-
NPRL1681	0.04000	BBB-
NPRL1682	0.16096	BBB-
NPRL1683	0.20318	BBB-
NPRL1684	0.76347	BBB+
NPRL1685	0.34642	BBB-

NPRL1686	0.05806	BBB-
NPRL1687	0.00354	BBB-
NPRL1688	0.28569	BBB-
NPRL1689	0.60748	BBB+
NPRL1690	0.15988	BBB-
NPRL1691	0.08867	BBB-
NPRL1692	0.01144	BBB-
NPRL1693	0.01904	BBB-
NPRL1694	0.04830	BBB-
NPRL1695	0.01442	BBB-
NPRL1696	0.25476	BBB-
NPRL1697	0.13772	BBB-
NPRL1698	0.92528	BBB+
NPRL1699	0.35795	BBB-
NPRL1700	0.03112	BBB-
NPRL1701	0.02070	BBB-
NPRL1702	0.16091	BBB-
NPRL1703	0.08076	BBB-
NPRL1704	0.00611	BBB-
NPRL1705	0.25910	BBB-
NPRL1706	0.22185	BBB-
NPRL1711	0.06059	BBB-
NPRL1712	0.09316	BBB-
NPRL1713	0.09025	BBB-
NPRL1716	0.12663	BBB-
NPRL1717	0.06110	BBB-
NPRL1718	0.02307	BBB-
NPRL1719	0.02182	BBB-
NPRL1720	0.06213	BBB-
NPRL1721	0.24486	BBB-
NPRL1722	0.08093	BBB-
NPRL1723	0.22623	BBB-
NPRL1724	0.73133	BBB+
NPRL1725	0.12380	BBB-
NPRL1726	0.11842	BBB-
NPRL1727	0.01032	BBB-
NPRL1728	0.18874	BBB-
NPRL1729	0.16330	BBB-
NPRL1730	0.04757	BBB-
NPRL1731	0.15797	BBB-
NPRL1732	0.03521	BBB-
NPRL1733	0.00466	BBB-
NPRL1734	0.01949	BBB-
NPRL1735	0.12295	BBB-
NPRL1736	0.02862	BBB-
NPRL1737	0.06315	BBB-
NPRL1738	0.12691	BBB-
NPRL1739	0.02982	BBB-
NPRL1740	0.02030	BBB-
NPRL1741	0.02952	BBB-
NPRL1742	0.02713	BBB-
NPRL1743	0.76373	BBB+
NPRL1744	0.53373	BBB+
NPRL1748	0.08409	BBB-
NPRL1751	0.00419	BBB-
NPRL1752	0.01065	BBB-
NPRL1753	0.44890	BBB-

NPRL1754	0.32619	BBB-
NPRL1755	0.15896	BBB-
NPRL1756	0.23633	BBB-
NPRL1757	0.23513	BBB-
NPRL1758	0.05873	BBB-
NPRL1759	0.03395	BBB-
NPRL1760	0.14533	BBB-
NPRL1761	0.41075	BBB-
NPRL1762	0.18653	BBB-
NPRL1763	0.21196	BBB-
NPRL1764	0.01007	BBB-
NPRL1765	0.82419	BBB+
NPRL1766	0.01911	BBB-
NPRL1767	0.04059	BBB-
NPRL1768	0.03084	BBB-
NPRL1769	0.01037	BBB-
NPRL1770	0.01826	BBB-
NPRL1771	0.30730	BBB-
NPRL1772	0.00226	BBB-
NPRL1773	0.01578	BBB-
NPRL1774	0.00264	BBB-
NPRL1775	0.01489	BBB-
NPRL1776	0.00291	BBB-
NPRL1777	0.00877	BBB-
NPRL1778	0.01433	BBB-
NPRL1779	0.19552	BBB-
NPRL1780	0.11626	BBB-
NPRL1781	0.01077	BBB-
NPRL1782	0.01828	BBB-
NPRL1783	0.00062	BBB-
NPRL1784	0.00020	BBB-
NPRL1785	0.00070	BBB-
NPRL1786	0.00009	BBB-
NPRL1787	0.00371	BBB-
NPRL1788	0.00275	BBB-
NPRL1789	0.00023	BBB-
NPRL1790	0.00098	BBB-
NPRL1791	0.37218	BBB-
NPRL1792	0.57573	BBB+
NPRL1793	0.32252	BBB-
NPRL1794	0.31052	BBB-
NPRL1795	0.06895	BBB-
NPRL1796	0.03361	BBB-
NPRL1797	0.00006	BBB-
NPRL1798	0.00002	BBB-
NPRL1799	0.51544	BBB+
NPRL1800	0.36244	BBB-
NPRL1801	0.80148	BBB+
NPRL1802	0.97030	BBB+
NPRL1803	0.95864	BBB+
NPRL1804	0.82353	BBB+
NPRL1805	0.95361	BBB+
NPRL1806	0.92331	BBB+
NPRL1807	0.00053	BBB-
NPRL1808	0.00242	BBB-
NPRL1809	0.65096	BBB+
NPRL1810	0.93325	BBB+

NPRL1811	0.66009	BBB+
NPRL1812	0.76653	BBB+
NPRL2208	0.78130	BBB+
NPRL2209	0.14413	BBB-
NPRL2210	0.98993	BBB+
NPRL2211	0.96324	BBB+
NPRL2212	0.95821	BBB+
NPRL2213	0.87960	BBB+
NPRL2214	0.98110	BBB+
NPRL2215	0.54430	BBB+
NPRL2216	0.79410	BBB+
NPRL2217	0.96519	BBB+
NPRL2218	0.51827	BBB+
NPRL2219	0.52689	BBB+
NPRL2220	0.00260	BBB-
NPRL2221	0.03699	BBB-
NPRL2222	0.11771	BBB-
NPRL2223	0.03911	BBB-
NPRL2224	0.24975	BBB-
NPRL2225	0.08389	BBB-
NPRL2226	0.00872	BBB-
NPRL2227	0.01475	BBB-
NPRL2228	0.18832	BBB-
NPRL2229	0.03359	BBB-
NPRL2230	0.25686	BBB-
NPRL2231	0.23614	BBB-
NPRL2232	0.34377	BBB-
NPRL2233	0.26122	BBB-
NPRL2234	0.00437	BBB-
NPRL2235	0.49136	BBB-
NPRL2236	0.41426	BBB-
NPRL2237	0.78724	BBB+
NPRL2238	0.70129	BBB+
NPRL2239	0.16578	BBB-
NPRL2240	0.48802	BBB-
NPRL2241	0.13881	BBB-
NPRL2242	0.28455	BBB-
NPRL2243	0.98180	BBB+
NPRL2244	0.02950	BBB-
NPRL2245	0.70664	BBB+
NPRL2246	0.41692	BBB-
NPRL2247	0.10568	BBB-
NPRL2248	0.14243	BBB-
NPRL2249	0.33843	BBB-
NPRL2250	0.04951	BBB-
NPRL2251	0.01119	BBB-
NPRL2252	0.22395	BBB-
NPRL2253	0.40486	BBB-
NPRL2254	0.29344	BBB-
NPRL2255	0.24124	BBB-
NPRL2256	0.26949	BBB-
NPRL2257	0.99208	BBB+
NPRL2258	0.95999	BBB+
NPRL2259	0.03501	BBB-
NPRL2260	0.99286	BBB+
NPRL2261	0.31305	BBB-
NPRL2262	0.55133	BBB+

NPRL2263	0.02454	BBB-
NPRL2266	0.44990	BBB-
NPRL2267	0.99568	BBB+
NPRL2268	0.80197	BBB+
NPRL2269	0.63495	BBB+
NPRL2270	0.43431	BBB-
NPRL2271	0.99981	BBB+
NPRL2272	0.90074	BBB+
NPRL2273	0.46580	BBB-
NPRL2274	0.98554	BBB+
NPRL2275	0.00018	BBB-
NPRL2276	0.98833	BBB+
NPRL2277	0.35413	BBB-
NPRL2278	0.32749	BBB-
NPRL2279	0.05433	BBB-
NPRL2280	0.08426	BBB-
NPRL2282	0.02548	BBB-
NPRL2283	0.79280	BBB+
NPRL2284	0.92262	BBB+
NPRL2285	0.47363	BBB-
NPRL2286	0.89620	BBB+
NPRL2287	0.60992	BBB+
NPRL2288	0.56445	BBB+
NPRL2289	0.42641	BBB-
NPRL2290	0.40504	BBB-
NPRL2291	0.99985	BBB+
NPRL2293	0.00632	BBB-
NPRL2294	0.19232	BBB-
NPRL2295	0.51158	BBB+
NPRL2296	0.94199	BBB+
NPRL2297	0.17266	BBB-
NPRL2298	0.00587	BBB-
NPRL2299	0.78884	BBB+
NPRL2300	0.98298	BBB+
NPRL2301	0.00032	BBB-
NPRL2302	0.72987	BBB+
NPRL2303	0.71365	BBB+
NPRL2304	0.00592	BBB-
NPRL2305	0.10944	BBB-
NPRL2306	0.53568	BBB+
NPRL2307	0.87744	BBB+
NPRL2308	0.01163	BBB-
NPRL2309	0.98495	BBB+
NPRL2310	0.37646	BBB-
NPRL2311	0.83982	BBB+
NPRL2312	0.57743	BBB+
NPRL2313	0.92295	BBB+
NPRL2314	0.71474	BBB+
NPRL2315	0.99761	BBB+
NPRL2316	0.96856	BBB+
NPRL2317	0.57538	BBB+
NPRL2318	0.89896	BBB+
NPRL2319	0.88895	BBB+
NPRL2320	0.89904	BBB+
NPRL2321	0.99607	BBB+
NPRL2322	0.22863	BBB-
NPRL2323	0.44589	BBB-

NPRL2324	0.94355	BBB+
NPRL2325	0.95951	BBB+
NPRL2326	0.90836	BBB+
NPRL2327	0.95714	BBB+
NPRL2328	0.87270	BBB+
NPRL2329	0.60938	BBB+
NPRL2330	0.00010	BBB-
NPRL2331	0.11483	BBB-
NPRL2332	0.99301	BBB+
NPRL2333	0.02605	BBB-
NPRL2334	0.94592	BBB+
NPRL2335	0.00065	BBB-
NPRL2336	0.04001	BBB-
NPRL2337	0.01272	BBB-
NPRL2338	0.03013	BBB-
NPRL2339	0.20190	BBB-
NPRL2340	0.00787	BBB-
NPRL2341	0.00337	BBB-
NPRL2343	0.27231	BBB-
NPRL2347	0.83033	BBB+
NPRL2348	0.00265	BBB-
NPRL2349	0.02799	BBB-
NPRL2350	0.00039	BBB-
NPRL2352	0.03613	BBB-
NPRL2354	0.05638	BBB-
NPRL2355	0.95565	BBB+
NPRL2356	0.00065	BBB-
NPRL2357	0.00039	BBB-
NPRL2358	0.00363	BBB-
NPRL2359	0.00001	BBB-
NPRL2360	0.00001	BBB-
NPRL2361	0.00006	BBB-
NPRL2362	0.00793	BBB-
NPRL2363	0.39932	BBB-
NPRL2364	0.81847	BBB+
NPRL2365	0.00102	BBB-
NPRL2366	0.02694	BBB-
NPRL2367	0.00172	BBB-
NPRL2368	0.00179	BBB-
NPRL2369	0.97103	BBB+
NPRL2370	0.01202	BBB-
NPRL2371	0.00500	BBB-
NPRL2372	0.00100	BBB-
NPRL2373	0.70292	BBB+
NPRL2374	0.01585	BBB-
NPRL2375	0.00014	BBB-
NPRL2376	0.43400	BBB-
NPRL2377	0.00320	BBB-
NPRL2378	0.54317	BBB+
NPRL2379	0.00086	BBB-
NPRL2380	0.00130	BBB-
NPRL2381	0.00003	BBB-
NPRL2382	0.06771	BBB-
NPRL2383	0.00358	BBB-
NPRL2384	0.00399	BBB-
NPRL2385	0.17978	BBB-
NPRL2386	0.00252	BBB-

NPRL2387	0.25580	BBB-
NPRL2388	0.11882	BBB-
NPRL2389	0.00137	BBB-
NPRL2390	0.31251	BBB-
NPRL2391	0.00320	BBB-
NPRL2392	0.00529	BBB-
NPRL2393	0.01348	BBB-
NPRL2395	0.99552	BBB+
NPRL2397	0.13469	BBB-
NPRL2398	0.98170	BBB+
NPRL2399	0.33598	BBB-
NPRL2400	0.13148	BBB-
NPRL2401	0.83169	BBB+
NPRL2402	0.00835	BBB-
NPRL2403	0.12489	BBB-
NPRL2404	0.05011	BBB-
NPRL2405	0.04204	BBB-
NPRL2406	0.19472	BBB-
NPRL2407	0.01016	BBB-
NPRL2408	0.02860	BBB-
NPRL2409	0.02987	BBB-
NPRL2410	0.00022	BBB-
NPRL2411	0.90228	BBB+
NPRL2414	0.10666	BBB-
NPRL2415	0.12366	BBB-
NPRL2416	0.32668	BBB-
NPRL2417	0.03305	BBB-
NPRL2418	0.42457	BBB-
NPRL2419	0.88024	BBB+
NPRL2420	0.98481	BBB+
NPRL2421	0.07356	BBB-
NPRL2422	0.26924	BBB-
NPRL2423	0.00029	BBB-
NPRL2424	0.00024	BBB-
NPRL2425	0.41829	BBB-
NPRL2426	0.02818	BBB-
NPRL2427	0.00016	BBB-
NPRL2428	0.00173	BBB-
NPRL2429	0.60574	BBB+
NPRL2430	0.32238	BBB-
NPRL2431	0.02693	BBB-
NPRL2432	0.78949	BBB+
NPRL2434	0.01269	BBB-
NPRL2435	0.00873	BBB-
NPRL2436	0.01488	BBB-
NPRL2437	0.00350	BBB-
NPRL2438	0.01261	BBB-
NPRL2439	0.33324	BBB-
NPRL2440	0.95456	BBB+
NPRL2441	0.06818	BBB-
NPRL2442	0.84324	BBB+
NPRL2443	0.10252	BBB-
NPRL2444	0.15677	BBB-
NPRL2445	0.69494	BBB+
NPRL2446	0.70905	BBB+
NPRL2447	0.93395	BBB+
NPRL2448	0.03824	BBB-

NPRL2449	0.18111	BBB-
NPRL2450	0.01187	BBB-
NPRL2452	0.99209	BBB+
NPRL2453	0.03513	BBB-
NPRL2455	0.99918	BBB+
NPRL2456	0.00005	BBB-
NPRL2457	0.01491	BBB-
NPRL2458	0.00005	BBB-
NPRL2460	0.00022	BBB-
NPRL2461	0.00746	BBB-
NPRL2462	0.01441	BBB-
NPRL2463	0.00048	BBB-
NPRL2464	0.00117	BBB-
NPRL2465	0.23413	BBB-
NPRL2466	0.43249	BBB-
NPRL2467	0.00043	BBB-
NPRL2468	0.56933	BBB+
NPRL2469	0.07522	BBB-
NPRL2470	0.01406	BBB-
NPRL2471	0.29817	BBB-
NPRL2472	0.56786	BBB+
NPRL2473	0.89217	BBB+
NPRL2474	0.03672	BBB-
NPRL2475	0.29217	BBB-
NPRL2476	0.86188	BBB+
NPRL2477	0.63973	BBB+
NPRL2478	0.21780	BBB-
NPRL2479	0.08047	BBB-
NPRL2480	0.14911	BBB-
NPRL2481	0.96624	BBB+
NPRL2482	0.96510	BBB+
NPRL2484	0.00113	BBB-
NPRL2485	0.00109	BBB-
NPRL2486	0.00054	BBB-
NPRL2487	0.00041	BBB-
NPRL2488	0.00819	BBB-
NPRL2489	0.00083	BBB-
NPRL2490	0.02549	BBB-
NPRL2491	0.00086	BBB-
NPRL2492	0.00123	BBB-
NPRL2493	0.04157	BBB-
NPRL2494	0.00321	BBB-
NPRL2495	0.02873	BBB-
NPRL2496	0.92358	BBB+
NPRL2497	0.15457	BBB-
NPRL2498	0.96933	BBB+
NPRL2499	0.02493	BBB-
NPRL2500	0.12380	BBB-
NPRL2501	0.16594	BBB-
NPRL2502	0.02076	BBB-
NPRL2503	0.10995	BBB-
NPRL2504	0.14469	BBB-
NPRL2505	0.01263	BBB-
NPRL2506	0.04153	BBB-
NPRL2507	0.18388	BBB-
NPRL2508	0.00600	BBB-
NPRL2509	0.13092	BBB-

NPRL2510	0.11084	BBB-
NPRL2511	0.06315	BBB-
NPRL2512	0.36628	BBB-
NPRL2513	0.02213	BBB-
NPRL2514	0.02050	BBB-
NPRL2515	0.08319	BBB-
NPRL2516	0.15908	BBB-
NPRL2517	0.63880	BBB+
NPRL2518	0.11780	BBB-
NPRL2519	0.02286	BBB-
NPRL2520	0.40902	BBB-
NPRL2521	0.16543	BBB-
NPRL2522	0.86130	BBB+
NPRL2523	0.99139	BBB+
NPRL2524	0.06060	BBB-
NPRL2525	0.16368	BBB-
NPRL2526	0.23010	BBB-
NPRL2527	0.17381	BBB-
NPRL2528	0.15219	BBB-
NPRL2529	0.39580	BBB-
NPRL2530	0.15028	BBB-
NPRL2531	0.18975	BBB-
NPRL2532	0.16807	BBB-
NPRL2533	0.28271	BBB-
NPRL2534	0.58114	BBB+
NPRL2535	0.37358	BBB-
NPRL2536	0.18235	BBB-
NPRL2537	0.07567	BBB-
NPRL2538	0.06092	BBB-
NPRL2539	0.03806	BBB-
NPRL2540	0.12081	BBB-
NPRL2541	0.27710	BBB-
NPRL2542	0.64052	BBB+
NPRL2543	0.97546	BBB+
NPRL2544	0.00345	BBB-
NPRL2545	0.77549	BBB+
NPRL2546	0.46089	BBB-
NPRL2547	0.74692	BBB+
NPRL2548	0.23006	BBB-
NPRL2549	0.74753	BBB+
NPRL2550	0.96218	BBB+
NPRL2551	0.00143	BBB-
NPRL2552	0.01135	BBB-
NPRL2554	0.00040	BBB-
NPRL2555	0.00148	BBB-
NPRL2556	0.15859	BBB-
NPRL2557	0.00113	BBB-
NPRL2558	0.00142	BBB-
NPRL2559	0.00072	BBB-
NPRL2561	0.08978	BBB-
NPRL2562	0.12991	BBB-
NPRL2564	0.01123	BBB-
NPRL2565	0.03786	BBB-
NPRL2566	0.42349	BBB-
NPRL2567	0.44715	BBB-
NPRL2569	0.74676	BBB+
NPRL2570	0.64353	BBB+

NPRL2571	0.00674	BBB-
NPRL2573	0.00220	BBB-
NPRL2574	0.00062	BBB-
NPRL2575	0.00086	BBB-
NPRL2576	0.00001	BBB-
NPRL2577	0.00012	BBB-
NPRL2578	0.00038	BBB-
NPRL2579	0.00089	BBB-
NPRL2580	0.00030	BBB-
NPRL2581	0.00032	BBB-
NPRL2582	0.09810	BBB-
NPRL2583	0.99805	BBB+
NPRL2584	0.00375	BBB-
NPRL2585	0.06489	BBB-
NPRL2586	0.99592	BBB+
NPRL2587	0.99970	BBB+
NPRL2588	0.90804	BBB+
NPRL2589	0.66248	BBB+
NPRL2591	0.98332	BBB+
NPRL2592	0.97840	BBB+
NPRL2593	0.99827	BBB+
NPRL2594	0.99962	BBB+
NPRL2595	0.99929	BBB+
NPRL2596	0.34941	BBB-
NPRL2597	0.87280	BBB+
NPRL2598	0.00530	BBB-
NPRL2599	0.00239	BBB-
NPRL2600	0.00489	BBB-
NPRL2603	0.08425	BBB-
NPRL2604	0.00492	BBB-
NPRL2605	0.00811	BBB-
NPRL2606	0.01697	BBB-
NPRL2607	0.02158	BBB-
NPRL2608	0.05549	BBB-
NPRL2609	0.37086	BBB-
NPRL2610	0.07454	BBB-
NPRL2611	0.90196	BBB+
NPRL2612	0.87037	BBB+
NPRL2613	0.46328	BBB-
NPRL2614	0.96204	BBB+
NPRL2615	0.91297	BBB+
NPRL2616	0.89468	BBB+
NPRL2617	0.88556	BBB+
NPRL2618	0.94992	BBB+
NPRL2619	0.72888	BBB+
NPRL2620	0.96951	BBB+
NPRL2621	0.99320	BBB+
NPRL2622	0.07340	BBB-
NPRL2623	0.83259	BBB+
NPRL2624	0.90910	BBB+
NPRL2625	0.00092	BBB-
NPRL2626	0.01923	BBB-
NPRL2627	0.02144	BBB-
NPRL2628	0.00042	BBB-
NPRL2629	0.00833	BBB-
NPRL2630	0.00114	BBB-
NPRL2631	0.00005	BBB-

NPRL2632	0.01932	BBB-
NPRL2633	0.26034	BBB-
NPRL2634	0.87516	BBB+
NPRL2635	0.00287	BBB-
NPRL2636	0.36212	BBB-
NPRL2637	0.00971	BBB-
NPRL2639	0.00094	BBB-
NPRL2640	0.03864	BBB-
NPRL2641	0.01142	BBB-
NPRL2642	0.08979	BBB-
NPRL2643	0.41584	BBB-
NPRL2644	0.00706	BBB-
NPRL2645	0.00070	BBB-
NPRL2646	0.00001	BBB-
NPRL2647	0.00030	BBB-
NPRL2648	0.77414	BBB+
NPRL2649	0.61354	BBB+
NPRL2650	0.00038	BBB-
NPRL2651	0.45628	BBB-
NPRL2652	0.02038	BBB-
NPRL2653	0.01289	BBB-
NPRL2654	0.04989	BBB-
NPRL2655	0.00210	BBB-
NPRL2657	0.00021	BBB-
NPRL2658	0.00210	BBB-
NPRL2659	0.00090	BBB-
NPRL2660	0.00129	BBB-
NPRL2661	0.00139	BBB-
NPRL2662	0.03404	BBB-
NPRL2663	0.85319	BBB+
NPRL2664	0.16101	BBB-
NPRL2665	0.68100	BBB+
NPRL2666	0.88585	BBB+
NPRL2667	0.24067	BBB-
NPRL2668	0.04668	BBB-
NPRL2669	0.01289	BBB-
NPRL2670	0.85799	BBB+
NPRL2671	0.69996	BBB+
NPRL2672	0.81549	BBB+
NPRL2673	0.94905	BBB+
NPRL2674	0.90583	BBB+
NPRL2675	0.86248	BBB+
NPRL2676	0.95193	BBB+
NPRL2677	0.04433	BBB-
NPRL2678	0.53503	BBB+
NPRL2679	0.17774	BBB-
NPRL2680	0.17782	BBB-
NPRL2681	0.25376	BBB-
NPRL2682	0.08501	BBB-
NPRL2683	0.24116	BBB-
NPRL2684	0.06740	BBB-
NPRL2685	0.02954	BBB-
NPRL2686	0.02669	BBB-
NPRL2687	0.02470	BBB-
NPRL2688	0.18248	BBB-
NPRL2689	0.01601	BBB-
NPRL2690	0.07813	BBB-

NPRL2691	0.50935	BBB+
NPRL2692	0.16228	BBB-
NPRL2693	0.11512	BBB-
NPRL2694	0.24188	BBB-
NPRL2695	0.18867	BBB-
NPRL2696	0.11088	BBB-
NPRL2697	0.08862	BBB-
NPRL2698	0.03480	BBB-
NPRL2699	0.13047	BBB-
NPRL26A27:I2738	0.06112	BBB-
NPRL2700	0.19968	BBB-
NPRL2701	0.19268	BBB-
NPRL2702	0.01264	BBB-
NPRL2703	0.02517	BBB-
NPRL2704	0.02054	BBB-
NPRL2705	0.05824	BBB-
NPRL2706	0.09957	BBB-
NPRL2707	0.50461	BBB+
NPRL2708	0.02662	BBB-
NPRL2709	0.72529	BBB+
NPRL2710	0.81979	BBB+
NPRL2711	0.01864	BBB-
NPRL2712	0.18917	BBB-
NPRL2713	0.06475	BBB-
NPRL2714	0.03655	BBB-
NPRL2715	0.52180	BBB+
NPRL2716	0.13035	BBB-
NPRL2717	0.36832	BBB-
NPRL2718	0.04645	BBB-
NPRL2719	0.20525	BBB-
NPRL2720	0.70135	BBB+
NPRL2721	0.74829	BBB+
NPRL2722	0.02170	BBB-
NPRL2723	0.05846	BBB-
NPRL2724	0.01151	BBB-
NPRL2725	0.65361	BBB+
NPRL2726	0.00950	BBB-
NPRL2728	0.05398	BBB-
NPRL2729	0.94238	BBB+
NPRL2730	0.02425	BBB-
NPRL2731	0.00444	BBB-
NPRL2732	0.00931	BBB-
NPRL2733	0.00684	BBB-
NPRL2734	0.02090	BBB-
NPRL2735	0.00185	BBB-
NPRL2736	0.00691	BBB-
NPRL2737	0.03334	BBB-
NPRL2738	0.00576	BBB-
NPRL2739	0.04423	BBB-
NPRL2740	0.10622	BBB-
NPRL2741	0.00986	BBB-
NPRL2742	0.11033	BBB-
NPRL2743	0.54836	BBB+
NPRL2745	0.00717	BBB-
NPRL2746	0.01275	BBB-
NPRL2747	0.05049	BBB-
NPRL2748	0.90855	BBB+

NPRL2749	0.88108	BBB+
NPRL2750	0.90900	BBB+
NPRL2751	0.00012	BBB-
NPRL2752	0.00022	BBB-
NPRL2753	0.03941	BBB-
NPRL2754	0.00160	BBB-
NPRL2755	0.41176	BBB-
NPRL2756	0.05217	BBB-
NPRL2757	0.00162	BBB-
NPRL2758	0.00369	BBB-
NPRL2759	0.01072	BBB-
NPRL2760	0.06272	BBB-
NPRL2761	0.00251	BBB-
NPRL2762	0.01498	BBB-
NPRL2763	0.00164	BBB-
NPRL2764	0.13764	BBB-
NPRL2765	0.19303	BBB-
NPRL2766	0.30047	BBB-
NPRL2767	0.00030	BBB-
NPRL2768	0.01599	BBB-
NPRL2769	0.00257	BBB-
NPRL2770	0.81563	BBB+
NPRL2771	0.02433	BBB-
NPRL2772	0.21214	BBB-
NPRL2773	0.00234	BBB-
NPRL2774	0.02288	BBB-
NPRL2775	0.29449	BBB-
NPRL2776	0.02709	BBB-
NPRL2777	0.00397	BBB-
NPRL2778	0.00812	BBB-
NPRL2779	0.01154	BBB-
NPRL2780	0.01146	BBB-
NPRL2782	0.85005	BBB+
NPRL2783	0.99470	BBB+
NPRL2784	0.98564	BBB+
NPRL2785	0.63798	BBB+
NPRL2786	0.99372	BBB+
NPRL2787	0.94531	BBB+
NPRL2788	0.78138	BBB+
NPRL2789	0.94676	BBB+
NPRL2790	0.93347	BBB+
NPRL2791	0.99907	BBB+
NPRL2792	0.83091	BBB+
NPRL2793	0.60076	BBB+
NPRL2794	0.63557	BBB+
NPRL2795	0.99950	BBB+
NPRL2796	0.97534	BBB+
NPRL2797	0.99717	BBB+
NPRL2798	0.59669	BBB+
NPRL2799	0.00460	BBB-
NPRL2800	0.04859	BBB-
NPRL2801	0.42769	BBB-
NPRL2802	0.52020	BBB+
NPRL2803	0.78277	BBB+
NPRL2804	0.33847	BBB-
NPRL2805	0.08994	BBB-
NPRL2806	0.99069	BBB+

NPRL2807	0.99377	BBB+
NPRL2808	0.97984	BBB+
NPRL2809	0.99825	BBB+
NPRL2810	0.03517	BBB-
NPRL2811	0.01720	BBB-
NPRL2813	0.81081	BBB+
NPRL2814	0.93396	BBB+
NPRL2815	0.88660	BBB+
NPRL2816	0.05765	BBB-
NPRL2817	0.37824	BBB-
NPRL2818	0.04129	BBB-
NPRL2819	0.62634	BBB+
NPRL2820	0.22829	BBB-
NPRL2821	0.53565	BBB+
NPRL2822	0.05069	BBB-
NPRL2823	0.29266	BBB-
NPRL2824	0.03312	BBB-
NPRL2825	0.05011	BBB-
NPRL2826	0.02839	BBB-
NPRL2827	0.46882	BBB-
NPRL2828	0.15369	BBB-
NPRL2829	0.40275	BBB-
NPRL2830	0.34543	BBB-
NPRL2831	0.70453	BBB+
NPRL2832	0.91813	BBB+
NPRL2833	0.00010	BBB-
NPRL2834	0.00005	BBB-
NPRL2835	0.00171	BBB-
NPRL2836	0.00038	BBB-
NPRL2837	0.00008	BBB-
NPRL2838	0.00005	BBB-
NPRL2839	0.00100	BBB-
NPRL2840	0.00012	BBB-
NPRL2841	0.00010	BBB-
NPRL2842	0.00012	BBB-
NPRL2843	0.00006	BBB-
NPRL2844	0.00004	BBB-
NPRL2845	0.00056	BBB-
NPRL2846	0.00001	BBB-
NPRL2847	0.00065	BBB-
NPRL2848	0.00032	BBB-
NPRL2849	0.00015	BBB-
NPRL2850	0.00100	BBB-
NPRL2851	0.00036	BBB-
NPRL2852	0.00039	BBB-
NPRL2853	0.06721	BBB-
NPRL2854	0.02185	BBB-
NPRL2856	0.35304	BBB-
NPRL2858	0.18685	BBB-
NPRL2859	0.42514	BBB-
NPRL2860	0.00020	BBB-
NPRL2861	0.00345	BBB-
NPRL2862	0.23980	BBB-
NPRL2863	0.98600	BBB+
NPRL2864	0.33083	BBB-
NPRL2865	0.00027	BBB-
NPRL2866	0.95544	BBB+

NPRL2867	0.01267	BBB-
NPRL2868	0.00094	BBB-
NPRL2869	0.00031	BBB-
NPRL2870	0.00056	BBB-
NPRL2871	0.00096	BBB-
NPRL2872	0.66005	BBB+
NPRL2873	0.00055	BBB-
NPRL2875	0.00477	BBB-
NPRL2877	0.00146	BBB-
NPRL2878	0.00007	BBB-
NPRL2879	0.00008	BBB-
NPRL2880	0.00019	BBB-
NPRL2881	0.00391	BBB-
NPRL2882	0.00056	BBB-
NPRL2883	0.00049	BBB-
NPRL2884	0.00063	BBB-
NPRL2885	0.00016	BBB-
NPRL2886	0.00016	BBB-
NPRL2887	0.00149	BBB-
NPRL2888	0.00126	BBB-
NPRL2889	0.00024	BBB-
NPRL2890	0.00047	BBB-
NPRL2891	0.00087	BBB-
NPRL2892	0.00215	BBB-
NPRL2893	0.00199	BBB-
NPRL2894	0.00006	BBB-
NPRL2895	0.00044	BBB-
NPRL2896	0.00065	BBB-
NPRL2897	0.00007	BBB-
NPRL2898	0.00012	BBB-
NPRL2899	0.00025	BBB-
NPRL2900	0.00046	BBB-
NPRL2901	0.00007	BBB-
NPRL2902	0.00038	BBB-
NPRL2904	0.02345	BBB-
NPRL2905	0.00036	BBB-
NPRL2906	0.01004	BBB-
NPRL2907	0.86119	BBB+
NPRL2908	0.00007	BBB-
NPRL2909	0.00008	BBB-
NPRL2910	0.00025	BBB-
NPRL2911	0.00003	BBB-
NPRL2912	0.01179	BBB-
NPRL2913	0.00122	BBB-
NPRL2914	0.77954	BBB+
NPRL2915	0.40954	BBB-
NPRL2916	0.52291	BBB+
NPRL2917	0.10272	BBB-
NPRL2918	0.39268	BBB-
NPRL2919	0.84858	BBB+
NPRL2920	0.62934	BBB+
NPRL2921	0.53025	BBB+
NPRL2922	0.75411	BBB+
NPRL2923	0.36593	BBB-
NPRL2924	0.04048	BBB-
NPRL2925	0.00839	BBB-
NPRL2926	0.00887	BBB-

NPRL2928	0.01122	BBB-
NPRL2929	0.02236	BBB-
NPRL2930	0.00300	BBB-
NPRL2933	0.13581	BBB-
NPRL2937	0.00005	BBB-
NPRL2938	0.99209	BBB+
NPRL2939	0.88477	BBB+
NPRL2940	0.10341	BBB-
NPRL2943	0.72700	BBB+
NPRL2944	0.88284	BBB+
NPRL2945	0.91057	BBB+
NPRL2946	0.00318	BBB-
NPRL2947	0.61606	BBB+
NPRL2948	0.98377	BBB+
NPRL2949	0.16351	BBB-
NPRL2950	0.57907	BBB+
NPRL2951	0.02513	BBB-
NPRL2952	0.00197	BBB-
NPRL2953	0.51883	BBB+
NPRL2954	0.00218	BBB-
NPRL2955	0.00105	BBB-
NPRL2956	0.25802	BBB-
NPRL2957	0.26997	BBB-
NPRL2958	0.07546	BBB-
NPRL2959	0.00011	BBB-
NPRL2961	0.99923	BBB+
NPRL2962	0.00511	BBB-
NPRL2963	0.87534	BBB+
NPRL2964	0.90347	BBB+
NPRL2965	0.13448	BBB-
NPRL2966	0.00810	BBB-
NPRL2968	0.99689	BBB+
NPRL2969	0.00110	BBB-
NPRL2970	0.85345	BBB+
NPRL2971	0.01158	BBB-
NPRL2972	0.93990	BBB+
NPRL2973	0.95159	BBB+
NPRL2974	0.00437	BBB-
NPRL2975	0.00173	BBB-
NPRL2976	0.00124	BBB-
NPRL2977	0.02453	BBB-
NPRL2978	0.03556	BBB-
NPRL2979	0.65916	BBB+
NPRL2980	0.96031	BBB+
NPRL2981	0.10146	BBB-
NPRL2983	0.09054	BBB-
NPRL2984	0.36139	BBB-
NPRL2985	0.82863	BBB+
NPRL2988	0.29619	BBB-
NPRL2990	0.03286	BBB-
NPRL2992	0.88668	BBB+
NPRL2993	0.63475	BBB+
NPRL2994	0.95261	BBB+
NPRL2995	0.94475	BBB+
NPRL2996	0.69398	BBB+
NPRL2997	0.99956	BBB+
NPRL2998	0.61171	BBB+

NPRL2999	0.80347	BBB+
NPRL3000	0.30119	BBB-
NPRL3001	0.71679	BBB+
NPRL3002	0.01996	BBB-
NPRL3003	0.29755	BBB-
NPRL3004	0.43857	BBB-
NPRL3005	0.00210	BBB-
NPRL3006	0.00147	BBB-
NPRL3007	0.04806	BBB-
NPRL3009	0.98004	BBB+
NPRL3010	0.01382	BBB-
NPRL3011	0.83180	BBB+
NPRL3012	0.98634	BBB+
NPRL3013	0.66725	BBB+
NPRL3014	0.00799	BBB-
NPRL3015	0.81909	BBB+
NPRL3016	0.00100	BBB-
NPRL3017	0.02041	BBB-
NPRL3018	0.01241	BBB-
NPRL3019	0.02225	BBB-
NPRL3020	0.82088	BBB+
NPRL3021	0.02304	BBB-
NPRL3022	0.00552	BBB-
NPRL3023	0.00460	BBB-
NPRL3024	0.05233	BBB-
NPRL3025	0.59787	BBB+
NPRL3026	0.00175	BBB-
NPRL3027	0.23639	BBB-
NPRL3029	0.00007	BBB-
NPRL3030	0.13679	BBB-
NPRL3031	0.00010	BBB-
NPRL3033	0.45444	BBB-
NPRL3034	0.92500	BBB+
NPRL3035	0.81701	BBB+
NPRL3036	0.02185	BBB-
NPRL3037	0.11230	BBB-
NPRL3038	0.00044	BBB-
NPRL3039	0.29345	BBB-
NPRL3040	0.79988	BBB+
NPRL3041	0.28772	BBB-
NPRL3043	0.35935	BBB-
NPRL3045	0.20072	BBB-
NPRL3046	0.52099	BBB+
NPRL3047	0.64034	BBB+
NPRL3048	0.24967	BBB-
NPRL3049	0.73382	BBB+
NPRL3050	0.00016	BBB-
NPRL3051	0.00015	BBB-
NPRL3052	0.00041	BBB-
NPRL3053	0.00018	BBB-
NPRL3054	0.93082	BBB+
NPRL3055	0.47075	BBB-
NPRL3056	0.62857	BBB+
NPRL3057	0.00263	BBB-
NPRL3058	0.00002	BBB-
NPRL3061	0.00003	BBB-
NPRL3062	0.01286	BBB-

NPRL3063	0.00015	BBB-
NPRL3064	0.00006	BBB-
NPRL3065	0.00033	BBB-
NPRL3066	0.05976	BBB-
NPRL3067	0.00121	BBB-
NPRL3069	0.00048	BBB-
NPRL3070	0.00081	BBB-
NPRL3071	0.00029	BBB-
NPRL3072	0.00157	BBB-
NPRL3073	0.46802	BBB-
NPRL3074	0.00095	BBB-
NPRL3075	0.00068	BBB-
NPRL3076	0.00482	BBB-
NPRL3077	0.00017	BBB-
NPRL3078	0.00732	BBB-
NPRL3079	0.00015	BBB-
NPRL3081	0.01000	BBB-
NPRL3082	0.00010	BBB-
NPRL3083	0.00080	BBB-
NPRL3084	0.00073	BBB-
NPRL3085	0.00060	BBB-
NPRL3086	0.00027	BBB-
NPRL3087	0.03851	BBB-
NPRL3088	0.17021	BBB-
NPRL3089	0.00788	BBB-
NPRL3090	0.58155	BBB+
NPRL3092	0.60979	BBB+
NPRL3093	0.25123	BBB-
NPRL3094	0.10022	BBB-
NPRL3095	0.85745	BBB+
NPRL3096	0.62410	BBB+
NPRL3098	0.00001	BBB-
NPRL3099	0.00029	BBB-
NPRL3100	0.00296	BBB-
NPRL3101	0.00432	BBB-
NPRL3102	0.62734	BBB+
NPRL3103	0.00181	BBB-
NPRL3104	0.00177	BBB-
NPRL3105	0.00145	BBB-
NPRL3106	0.82252	BBB+
NPRL3109	0.00117	BBB-
NPRL3111	0.38994	BBB-
NPRL3112	0.00312	BBB-
NPRL3113	0.99918	BBB+
NPRL3114	0.00010	BBB-
NPRL3115	0.00454	BBB-
NPRL3116	0.00160	BBB-
NPRL3117	0.00086	BBB-
NPRL3118	0.00299	BBB-
NPRL3119	0.89921	BBB+
NPRL3120	0.97468	BBB+
NPRL3121	0.92336	BBB+
NPRL3122	0.08567	BBB-
NPRL3123	0.50630	BBB+
NPRL3124	0.99554	BBB+
NPRL3125	0.88979	BBB+
NPRL3126	0.20189	BBB-

NPRL3127	0.93473	BBB+
NPRL3128	0.98710	BBB+
NPRL3129	0.00261	BBB-
NPRL3130	0.00195	BBB-
NPRL3131	0.81099	BBB+
NPRL3132	0.72224	BBB+
NPRL3133	0.97363	BBB+
NPRL3134	0.31938	BBB-
NPRL3135	0.91365	BBB+
NPRL3136	0.00407	BBB-
NPRL3137	0.45043	BBB-
NPRL3138	0.01529	BBB-
NPRL3139	0.95642	BBB+
NPRL3140	0.57399	BBB+
NPRL3141	0.91468	BBB+
NPRL3142	0.99305	BBB+
NPRL3143	0.19746	BBB-
NPRL3144	0.85753	BBB+
NPRL3145	0.81043	BBB+
NPRL3146	0.01722	BBB-
NPRL3147	0.00395	BBB-
NPRL3148	0.00554	BBB-
NPRL3149	0.01538	BBB-
NPRL3150	0.07806	BBB-
NPRL3151	0.26922	BBB-
NPRL3152	0.06688	BBB-
NPRL3153	0.05937	BBB-
NPRL3154	0.99985	BBB+
NPRL3155	0.99984	BBB+
NPRL3156	0.99986	BBB+
NPRL3157	0.99985	BBB+
NPRL3158	0.94581	BBB+
NPRL3159	0.00293	BBB-
NPRL3160	0.98860	BBB+
NPRL3162	0.98432	BBB+
NPRL3163	0.98390	BBB+
NPRL3164	0.96699	BBB+
NPRL3165	0.98942	BBB+
NPRL3166	0.98867	BBB+
NPRL3169	0.41565	BBB-
NPRL3170	0.91860	BBB+
NPRL3171	0.99477	BBB+
NPRL3172	0.82048	BBB+
NPRL3173	0.63969	BBB+
NPRL3174	0.09159	BBB-
NPRL3175	0.38585	BBB-
NPRL3176	0.11241	BBB-
NPRL3177	0.96086	BBB+
NPRL3179	0.11473	BBB-
NPRL3181	0.89082	BBB+
NPRL3182	0.95034	BBB+
NPRL3183	0.00002	BBB-
NPRL3184	0.00457	BBB-
NPRL3185	0.01022	BBB-
NPRL3186	0.61112	BBB+
NPRL3187	0.99892	BBB+
NPRL3188	0.11173	BBB-

NPRL3189	0.82178	BBB+
NPRL3190	0.85877	BBB+
NPRL3191	0.93364	BBB+
NPRL3192	0.94498	BBB+
NPRL3193	0.99366	BBB+
NPRL3194	0.99250	BBB+
NPRL3195	0.88052	BBB+
NPRL3196	0.38682	BBB-
NPRL3197	0.00117	BBB-
NPRL3198	0.01318	BBB-
NPRL3199	0.00271	BBB-
NPRL3200	0.08250	BBB-
NPRL3202	0.01752	BBB-
NPRL3203	0.95929	BBB+
NPRL3204	0.02743	BBB-
NPRL3205	0.01826	BBB-
NPRL3206	0.64282	BBB+
NPRL3207	0.03533	BBB-
NPRL3208	0.13442	BBB-
NPRL3209	0.00199	BBB-
NPRL3210	0.08672	BBB-
NPRL3211	0.23695	BBB-
NPRL3212	0.00235	BBB-
NPRL3213	0.99960	BBB+
NPRL3214	0.12237	BBB-
NPRL3215	0.14140	BBB-
NPRL3217	0.83596	BBB+
NPRL3218	0.36242	BBB-
NPRL3220	0.01488	BBB-
NPRL3222	0.00005	BBB-
NPRL3223	0.68403	BBB+
NPRL3224	0.00402	BBB-
NPRL3225	0.13852	BBB-
NPRL3226	0.16650	BBB-
NPRL3227	0.07023	BBB-
NPRL3228	0.20660	BBB-
NPRL3229	0.29300	BBB-
NPRL3231	0.26205	BBB-
NPRL3232	0.00121	BBB-
NPRL3233	0.03311	BBB-
NPRL3234	0.05762	BBB-
NPRL3235	0.94592	BBB+
NPRL3236	0.94678	BBB+
NPRL3237	0.45636	BBB-
NPRL3238	0.66445	BBB+
NPRL3241	0.12913	BBB-
NPRL3242	0.51211	BBB+
NPRL3245	0.04978	BBB-
NPRL3246	0.00922	BBB-
NPRL3247	0.02943	BBB-
NPRL3251	0.12984	BBB-
NPRL3252	0.95456	BBB+
NPRL3253	0.19919	BBB-
NPRL3254	0.00742	BBB-
NPRL3256	0.43552	BBB-
NPRL3258	0.00427	BBB-
NPRL3259	0.00555	BBB-

NPRL3261	0.01086	BBB-
NPRL3262	0.67834	BBB+
NPRL3263	0.31866	BBB-
NPRL3264	0.33733	BBB-
NPRL3265	0.29359	BBB-
NPRL3266	0.81532	BBB+
NPRL3267	0.41467	BBB-
NPRL3268	0.01151	BBB-
NPRL3269	0.02630	BBB-
NPRL3270	0.00047	BBB-
NPRL3271	0.00058	BBB-
NPRL3272	0.00089	BBB-
NPRL3274	0.01104	BBB-
NPRL3275	0.12092	BBB-
NPRL3276	0.00293	BBB-
NPRL3279	0.06509	BBB-
NPRL3280	0.06125	BBB-
NPRL3283	0.37565	BBB-
NPRL3284	0.31613	BBB-
NPRL3285	0.98664	BBB+
NPRL3286	0.97901	BBB+
NPRL3288	0.00027	BBB-
NPRL3291	0.96790	BBB+
NPRL3292	0.80517	BBB+
NPRL3295	0.99330	BBB+
NPRL3296	0.71079	BBB+
NPRL3297	0.21723	BBB-
NPRL3298	0.98286	BBB+
NPRL3299	0.48566	BBB-
NPRL3302	0.01367	BBB-
NPRL3303	0.03230	BBB-
NPRL3305	0.00450	BBB-
NPRL3306	0.20271	BBB-
NPRL3307	0.00220	BBB-
NPRL3308	0.35303	BBB-
NPRL3309	0.01798	BBB-
NPRL3310	0.93878	BBB+
NPRL3311	0.06233	BBB-
NPRL3312	0.29009	BBB-
NPRL3313	0.05900	BBB-
NPRL3315	0.00042	BBB-
NPRL3316	0.00449	BBB-
NPRL3317	0.03115	BBB-
NPRL3319	0.00028	BBB-
NPRL3321	0.00380	BBB-
NPRL3323	0.98739	BBB+
NPRL3324	0.97950	BBB+
NPRL3325	0.00031	BBB-
NPRL3328	0.00009	BBB-
NPRL3332	0.69128	BBB+
NPRL3333	0.09879	BBB-
NPRL3334	0.00073	BBB-
NPRL3335	0.93390	BBB+
NPRL3336	0.79027	BBB+
NPRL3337	0.00392	BBB-
NPRL3338	0.00901	BBB-
NPRL3339	0.02060	BBB-

NPRL3340	0.18829	BBB-
NPRL3341	0.80426	BBB+
NPRL3342	0.16148	BBB-
NPRL3343	0.64251	BBB+
NPRL3344	0.63458	BBB+
NPRL3346	0.99762	BBB+
NPRL3347	0.98691	BBB+
NPRL3348	0.99774	BBB+
NPRL3349	0.99871	BBB+
NPRL3350	0.91638	BBB+
NPRL3352	0.98158	BBB+
NPRL3353	0.99823	BBB+
NPRL3354	0.99622	BBB+
NPRL3355	0.99802	BBB+
NPRL3356	0.99851	BBB+
NPRL3357	0.96470	BBB+
NPRL3359	0.86872	BBB+
NPRL3360	0.99335	BBB+
NPRL3361	0.01712	BBB-
NPRL3362	0.09598	BBB-
NPRL3363	0.26413	BBB-
NPRL3364	0.01950	BBB-
NPRL3365	0.05679	BBB-
NPRL3366	0.13044	BBB-
NPRL3367	0.00507	BBB-
NPRL3368	0.35348	BBB-
NPRL3369	0.32007	BBB-
NPRL3370	0.48150	BBB-
NPRL3372	0.80605	BBB+
NPRL3373	0.88022	BBB+
NPRL3374	0.00616	BBB-
NPRL3375	0.10392	BBB-
NPRL3376	0.30339	BBB-
NPRL3377	0.00077	BBB-
NPRL3378	0.66910	BBB+
NPRL3379	0.78555	BBB+
NPRL3380	0.30490	BBB-
NPRL3381	0.67824	BBB+
NPRL3382	0.59922	BBB+
NPRL3383	0.79610	BBB+
NPRL3386	0.92396	BBB+
NPRL3390	0.11870	BBB-
NPRL3391	0.36982	BBB-
NPRL3393	0.98181	BBB+
NPRL3394	0.84155	BBB+
NPRL3395	0.00142	BBB-
NPRL3396	0.02247	BBB-
NPRL3397	0.53286	BBB+
NPRL3399	0.75473	BBB+
NPRL3400	0.02096	BBB-
NPRL3401	0.89425	BBB+
NPRL3402	0.01502	BBB-
NPRL3403	0.01740	BBB-
NPRL3404	0.00731	BBB-
NPRL3405	0.30511	BBB-
NPRL3406	0.07938	BBB-
NPRL3407	0.02467	BBB-

NPRL3416	0.95637	BBB+
NPRL3417	0.84872	BBB+
NPRL3426	0.22678	BBB-
NPRL3429	0.11914	BBB-
NPRL3430	0.03285	BBB-
NPRL3431	0.03119	BBB-
NPRL3434	0.00883	BBB-
NPRL3437	0.00048	BBB-
NPRL3441	0.00395	BBB-
NPRL3448	0.07129	BBB-
NPRL3449	0.04891	BBB-
NPRL3459	0.97965	BBB+
NPRL3462	0.44509	BBB-
NPRL3463	0.85344	BBB+
NPRL3464	0.55125	BBB+
NPRL3465	0.06148	BBB-
NPRL3466	0.83826	BBB+
NPRL3467	0.16636	BBB-
NPRL3468	0.98180	BBB+
NPRL3469	0.87459	BBB+
NPRL3470	0.93573	BBB+
NPRL3471	0.92971	BBB+
NPRL3472	0.04641	BBB-
NPRL3473	0.08759	BBB-
NPRL3474	0.58136	BBB+
NPRL3475	0.72464	BBB+
NPRL3476	0.41644	BBB-
NPRL3478	0.62066	BBB+
NPRL3479	0.77966	BBB+
NPRL3480	0.55331	BBB+
NPRL3481	0.37301	BBB-
NPRL3482	0.79066	BBB+
NPRL3483	0.79164	BBB+
NPRL3484	0.05342	BBB-
NPRL3485	0.14157	BBB-
NPRL3486	0.64434	BBB+
NPRL3487	0.20832	BBB-
NPRL3488	0.08943	BBB-
NPRL3489	0.01488	BBB-
NPRL3491	0.92546	BBB+
NPRL3492	0.93988	BBB+
NPRL3494	0.93176	BBB+
NPRL3495	0.68722	BBB+
NPRL3496	0.82070	BBB+
NPRL3498	0.97551	BBB+
NPRL3499	0.89892	BBB+
NPRL3500+A79:K79	0.69584	BBB+
NPRL3501	0.99989	BBB+
NPRL3502	0.96127	BBB+
NPRL3503	0.98328	BBB+
NPRL3504	0.71078	BBB+
NPRL3505	0.24988	BBB-
NPRL3506	0.95408	BBB+
NPRL3507	0.92698	BBB+
NPRL3508	0.99883	BBB+
NPRL3509	0.96884	BBB+
NPRL3510	0.94995	BBB+

NPRL3511	0.99795	BBB+
NPRL3512	0.99008	BBB+
NPRL3513	0.95424	BBB+
NPRL3514	0.84682	BBB+
NPRL3515	0.99947	BBB+
NPRL3516	0.99456	BBB+
NPRL3517	0.96595	BBB+
NPRL3518	0.96174	BBB+
NPRL3519	0.99559	BBB+
NPRL3520	0.13224	BBB-
NPRL3521	0.49588	BBB-
NPRL3522	0.04155	BBB-
NPRL3523	0.17181	BBB-
NPRL3524	0.23305	BBB-
NPRL3525	0.79091	BBB+
NPRL3526	0.51154	BBB+
NPRL3527	0.64101	BBB+
NPRL3528	0.87696	BBB+
NPRL3529	0.89873	BBB+
NPRL3530	0.46309	BBB-
NPRL3531	0.91777	BBB+
NPRL3532	0.37194	BBB-
NPRL3533	0.06627	BBB-
NPRL3536	0.16622	BBB-
NPRL3537	0.00072	BBB-
NPRL3538	0.81554	BBB+
NPRL3539	0.28208	BBB-
NPRL3540	0.78919	BBB+
NPRL3541	0.56277	BBB+
NPRL3542	0.78161	BBB+
NPRL3543	0.75892	BBB+
NPRL3544	0.18587	BBB-
NPRL3545	0.00311	BBB-
NPRL3547	0.53266	BBB+
NPRL3549	0.05411	BBB-
NPRL3551	0.00840	BBB-
NPRL3554	0.12550	BBB-
NPRL3555	0.09342	BBB-
NPRL3556	0.19136	BBB-
NPRL3557	0.37996	BBB-
NPRL3558	0.40546	BBB-
NPRL3559	0.28231	BBB-
NPRL3561	0.14611	BBB-
NPRL3562	0.01949	BBB-
NPRL3564	0.00126	BBB-
NPRL3565	0.00132	BBB-
NPRL3566	0.02965	BBB-
NPRL3567	0.03754	BBB-
NPRL3568	0.02409	BBB-
NPRL3569	0.05696	BBB-
NPRL3572	0.60808	BBB+
NPRL3573	0.37896	BBB-
NPRL3574	0.90130	BBB+
NPRL3575	0.93467	BBB+
NPRL3576	0.00145	BBB-
NPRL3577	0.97874	BBB+
NPRL3578	0.93307	BBB+

NPRL3579	0.99520	BBB+
NPRL3580	0.74140	BBB+
NPRL3581	0.96000	BBB+
NPRL3582	0.69936	BBB+
NPRL3583	0.98101	BBB+
NPRL3584	0.80118	BBB+
NPRL3585	0.88578	BBB+
NPRL3586	0.99245	BBB+
NPRL3587	0.82425	BBB+
NPRL3588	0.87796	BBB+
NPRL3589	0.33434	BBB-
NPRL3590	0.95214	BBB+
NPRL3591	0.92858	BBB+
NPRL3592	0.61969	BBB+
NPRL3593	0.90651	BBB+
NPRL3594	0.00368	BBB-
NPRL3595	0.12147	BBB-
NPRL3597	0.00010	BBB-
NPRL3599	0.00687	BBB-
NPRL3600	0.00125	BBB-
NPRL3601	0.01468	BBB-
NPRL3602	0.01034	BBB-
NPRL3603	0.00003	BBB-
NPRL3604	0.00129	BBB-
NPRL3605	0.00188	BBB-
NPRL3606	0.00087	BBB-
NPRL3607	0.05893	BBB-
NPRL3608	0.69520	BBB+
NPRL3609	0.01755	BBB-
NPRL3610	0.02205	BBB-
NPRL3612	0.99921	BBB+
NPRL3613	0.99689	BBB+
NPRL3614	0.99813	BBB+
NPRL3616	0.39959	BBB-
NPRL3617	0.99238	BBB+
NPRL3618	0.93210	BBB+
NPRL3619	0.67298	BBB+
NPRL3620	0.12757	BBB-
NPRL3622	0.97700	BBB+
NPRL3623	0.57287	BBB+
NPRL3624	0.88336	BBB+
NPRL3625	0.86026	BBB+
NPRL3627	0.91574	BBB+
NPRL3628	0.01966	BBB-
NPRL3629	0.00124	BBB-
NPRL3630	0.65003	BBB+
NPRL3631	0.87850	BBB+
NPRL3632	0.50246	BBB+
NPRL3633	0.00210	BBB-
NPRL3634	0.94717	BBB+
NPRL3635	0.22526	BBB-
NPRL3636	0.03469	BBB-
NPRL3637	0.00260	BBB-
NPRL3638	0.67597	BBB+
NPRL3639	0.54311	BBB+
NPRL3641	0.74437	BBB+
NPRL3642	0.97761	BBB+

NPRL3644	0.99543	BBB+
NPRL3645	0.86062	BBB+
NPRL3646	0.25106	BBB-
NPRL3647	0.78166	BBB+
NPRL3648	0.58074	BBB+
NPRL3649	0.95700	BBB+
NPRL3650	0.95145	BBB+
NPRL3652	0.03785	BBB-
NPRL3653	0.37251	BBB-
NPRL3654	0.09584	BBB-
NPRL3655	0.07052	BBB-
NPRL3656	0.00032	BBB-
NPRL3659	0.99594	BBB+
NPRL3660	0.96871	BBB+
NPRL3661	0.93537	BBB+
NPRL3665	0.84052	BBB+
NPRL3667	0.46020	BBB-
NPRL3669	0.79339	BBB+
NPRL3671	0.97476	BBB+
NPRL3673	0.92631	BBB+
NPRL3674	0.99811	BBB+
NPRL3677	0.75410	BBB+
NPRL3678	0.90466	BBB+
NPRL3679	0.89841	BBB+
NPRL3680	0.97224	BBB+
NPRL3682	0.60299	BBB+
NPRL3684	0.56288	BBB+
NPRL3685	0.01209	BBB-
NPRL3686	0.12414	BBB-
NPRL3687	0.94737	BBB+
NPRL3688	0.40159	BBB-
NPRL3690	0.04030	BBB-
NPRL3691	0.00320	BBB-
NPRL3692	0.05744	BBB-
NPRL3693	0.00211	BBB-
NPRL3694	0.14641	BBB-
NPRL3695	0.02435	BBB-
NPRL3696	0.09609	BBB-
NPRL3697	0.09536	BBB-
NPRL3698	0.89260	BBB+
NPRL3699	0.92040	BBB+
NPRL3700	0.91377	BBB+
NPRL3702	0.46512	BBB-
NPRL3703	0.85562	BBB+
NPRL3704	0.08501	BBB-
NPRL3705	0.85347	BBB+
NPRL3706	0.99683	BBB+
NPRL3707	0.06265	BBB-
NPRL3708	0.81276	BBB+
NPRL3709	0.97448	BBB+
NPRL3710	0.98208	BBB+
NPRL3711	0.61101	BBB+
NPRL3712	0.94636	BBB+
NPRL3713	0.99307	BBB+
NPRL3714	0.99220	BBB+
NPRL3715	0.77007	BBB+
NPRL3716	0.93906	BBB+

NPRL3717	0.70899	BBB+
NPRL3718	0.87532	BBB+
NPRL3719	0.99684	BBB+
NPRL3720	0.77125	BBB+
NPRL3721	0.96562	BBB+
NPRL3722	0.29900	BBB-
NPRL3723	0.25707	BBB-
NPRL3724	0.99042	BBB+
NPRL3725	0.42828	BBB-
NPRL3726	0.11972	BBB-
NPRL3727	0.93853	BBB+
NPRL3728	0.98406	BBB+
NPRL3729	0.98932	BBB+
NPRL3730	0.99452	BBB+
NPRL3731	0.31918	BBB-
NPRL3732	0.99463	BBB+
NPRL3733	0.99405	BBB+
NPRL3734	0.87496	BBB+
NPRL3735	0.97959	BBB+
NPRL3736	0.73468	BBB+
NPRL3737	0.05921	BBB-
NPRL3739	0.23099	BBB-
NPRL3740	0.25247	BBB-
NPRL3741	0.98416	BBB+
NPRL3742	0.35544	BBB-
NPRL3743	0.95091	BBB+
NPRL3744	0.99424	BBB+
NPRL3746	0.95177	BBB+
NPRL3747	0.94342	BBB+
NPRL3748	0.02398	BBB-
NPRL3749	0.82890	BBB+
NPRL3750	0.92811	BBB+
NPRL3751	0.31342	BBB-
NPRL3752	0.54087	BBB+
NPRL3753	0.89705	BBB+
NPRL3754	0.00132	BBB-
NPRL3755	0.95098	BBB+
NPRL3756	0.93083	BBB+
NPRL3758	0.98571	BBB+
NPRL3759	0.99357	BBB+
NPRL3760	0.91367	BBB+
NPRL3761	0.99875	BBB+
NPRL3762	0.98743	BBB+
NPRL3763	0.99707	BBB+
NPRL3764	0.93773	BBB+
NPRL3765	0.52921	BBB+
NPRL3766	0.24431	BBB-
NPRL3767	0.99658	BBB+
NPRL3768	0.88073	BBB+
NPRL3769	0.99157	BBB+
NPRL3770	0.99866	BBB+
NPRL3771	0.99422	BBB+
NPRL3772	0.99279	BBB+
NPRL3773	0.05715	BBB-
NPRL3774	0.99066	BBB+
NPRL3775	0.99213	BBB+
NPRL3776	0.99821	BBB+

NPRL3777	0.99855	BBB+
NPRL3778	0.99456	BBB+
NPRL3779	0.98918	BBB+
NPRL3781	0.57055	BBB+
NPRL3782	0.32602	BBB-
NPRL3783	0.38777	BBB-
NPRL3785	0.86654	BBB+
NPRL3786	0.00134	BBB-
NPRL3787	0.00475	BBB-
NPRL3788	0.92865	BBB+
NPRL3792	0.92001	BBB+
NPRL3793	0.00025	BBB-
NPRL3795	0.05617	BBB-
NPRL3796	0.56382	BBB+
NPRL3797	0.00039	BBB-
NPRL3798	0.98346	BBB+
NPRL3799	0.15001	BBB-
NPRL3800	0.88659	BBB+
NPRL3801	0.00997	BBB-
NPRL3802	0.00726	BBB-
NPRL3803	0.00434	BBB-
NPRL3804	0.68571	BBB+
NPRL3805	0.33422	BBB-
NPRL3806	0.00834	BBB-
NPRL3807	0.99670	BBB+
NPRL3808	0.00020	BBB-
NPRL3809	0.00077	BBB-
NPRL3812	0.00974	BBB-
NPRL3813	0.00190	BBB-
NPRL3814	0.00039	BBB-
NPRL3815	0.00072	BBB-
NPRL3817	0.00728	BBB-
NPRL3818	0.31385	BBB-
NPRL3819	0.26588	BBB-
NPRL3820	0.10119	BBB-
NPRL3821	0.85930	BBB+
NPRL3825	0.02367	BBB-
NPRL3826	0.04296	BBB-
NPRL3827	0.06840	BBB-
NPRL3828	0.78090	BBB+
NPRL3829	0.00960	BBB-
NPRL3830	0.00426	BBB-
NPRL3831	0.04822	BBB-
NPRL3832	0.01487	BBB-
NPRL3833	0.01891	BBB-
NPRL3834	0.01653	BBB-
NPRL3835	0.33535	BBB-
NPRL3837	0.31204	BBB-
NPRL3839	0.00570	BBB-
NPRL3840	0.00568	BBB-
NPRL3841	0.03025	BBB-
NPRL3842	0.02648	BBB-
NPRL3843	0.00204	BBB-
NPRL3844	0.73903	BBB+
NPRL3845	0.11935	BBB-
NPRL3846	0.10615	BBB-
NPRL3847	0.01064	BBB-

NPRL3848	0.00757	BBB-
NPRL3849	0.00710	BBB-
NPRL3850	0.05683	BBB-
NPRL3851	0.00166	BBB-
NPRL3852	0.00161	BBB-
NPRL3853	0.00114	BBB-
NPRL3854	0.00067	BBB-
NPRL3855	0.18990	BBB-
NPRL3856	0.00056	BBB-
NPRL3857	0.01592	BBB-
NPRL3858	0.95724	BBB+
NPRL3863	0.98063	BBB+
NPRL3864	0.93827	BBB+
NPRL3865	0.93455	BBB+
NPRL3866	0.36938	BBB-
NPRL3867	0.03946	BBB-
NPRL3868	0.19211	BBB-
NPRL3869	0.27469	BBB-
NPRL3870	0.00323	BBB-
NPRL3871	0.14326	BBB-
NPRL3872	0.98191	BBB+
NPRL3873	0.96620	BBB+
NPRL3874	0.05403	BBB-
NPRL3875	0.42505	BBB-
NPRL3876	0.30295	BBB-
NPRL3877	0.05278	BBB-
NPRL3878	0.03366	BBB-
NPRL3879	0.55932	BBB+
NPRL3880	0.23601	BBB-
NPRL3881	0.05482	BBB-
NPRL3883	0.01632	BBB-
NPRL3885	0.00808	BBB-
NPRL3888	0.07560	BBB-
NPRL3889	0.02315	BBB-
NPRL3890	0.89448	BBB+
NPRL3891	0.00017	BBB-
NPRL3892	0.14962	BBB-
NPRL3893	0.09328	BBB-
NPRL3894	0.00501	BBB-
NPRL3895	0.57387	BBB+
NPRL3896	0.82541	BBB+
NPRL3897	0.56998	BBB+
NPRL3898	0.85626	BBB+
NPRL3899	0.91503	BBB+
NPRL3900	0.75341	BBB+
NPRL3901	0.95752	BBB+
NPRL3902	0.94685	BBB+
NPRL3903	0.00293	BBB-
NPRL3904	0.54661	BBB+
NPRL3905	0.13992	BBB-
NPRL3906	0.11139	BBB-
NPRL3908	0.03323	BBB-
NPRL3909	0.00244	BBB-
NPRL3910	0.74887	BBB+
NPRL3911	0.04425	BBB-
NPRL3914	0.00865	BBB-
NPRL3915	0.99355	BBB+

NPRL3916	0.96932	BBB+
NPRL3917	0.99375	BBB+
NPRL3918	0.99817	BBB+
NPRL3919	0.97231	BBB+
NPRL3920	0.83756	BBB+
NPRL3921	0.99559	BBB+
NPRL3922	0.99486	BBB+
NPRL3923	0.98483	BBB+
NPRL3924	0.05887	BBB-
NPRL3925	0.00041	BBB-
NPRL3926	0.00037	BBB-
NPRL3927	0.00031	BBB-
NPRL3930	0.19350	BBB-
NPRL3931	0.54216	BBB+
NPRL3932	0.60171	BBB+
NPRL3933	0.39294	BBB-
NPRL3936	0.01255	BBB-
NPRL3937	0.02447	BBB-
NPRL3939	0.00220	BBB-
NPRL3940	0.00031	BBB-
NPRL3941	0.04867	BBB-
NPRL3942	0.01556	BBB-
NPRL3943	0.03737	BBB-
NPRL3944	0.11682	BBB-
NPRL3945	0.01768	BBB-
NPRL3946	0.00092	BBB-
NPRL3947	0.00926	BBB-
NPRL3949	0.05785	BBB-
NPRL3951	0.09914	BBB-
NPRL3953	0.04622	BBB-
NPRL3954	0.00173	BBB-
NPRL3955	0.00949	BBB-
NPRL3956	0.12670	BBB-
NPRL3957	0.93449	BBB+
NPRL3958	0.01327	BBB-
NPRL3960	0.06355	BBB-
NPRL3961	0.49487	BBB-
NPRL3962	0.02298	BBB-
NPRL3963	0.87914	BBB+
NPRL3964	0.56937	BBB+
NPRL3965	0.02263	BBB-
NPRL3966	0.45116	BBB-
NPRL3967	0.00708	BBB-
NPRL3968	0.09864	BBB-
NPRL3969	0.00107	BBB-
NPRL3970	0.95734	BBB+
NPRL3971	0.90599	BBB+
NPRL3972	0.09945	BBB-
NPRL3973	0.12821	BBB-
NPRL3974	0.00338	BBB-
NPRL3975	0.66130	BBB+
NPRL3976	0.03673	BBB-
NPRL3977	0.05628	BBB-
NPRL3978	0.02511	BBB-
NPRL3979	0.00046	BBB-
NPRL3980	0.20280	BBB-
NPRL3982	0.00042	BBB-

NPRL3983	0.07647	BBB-
NPRL3984	0.92346	BBB+
NPRL3985	0.00080	BBB-
NPRL3986	0.00128	BBB-
NPRL3987	0.00169	BBB-
NPRL3988	0.00073	BBB-
NPRL3989	0.00052	BBB-
NPRL3990	0.63219	BBB+
NPRL3992	0.00015	BBB-
NPRL3994	0.41035	BBB-
NPRL3995	0.04576	BBB-
NPRL3996	0.03507	BBB-
NPRL3997	0.06100	BBB-
NPRL3998	0.13006	BBB-
NPRL3999	0.08542	BBB-
NPRL4000	0.18632	BBB-
NPRL4001	0.00018	BBB-
NPRL4002	0.30561	BBB-
NPRL4003	0.71589	BBB+
NPRL4004	0.10618	BBB-
NPRL4006	0.00467	BBB-
NPRL4007	0.00908	BBB-
NPRL4008	0.00943	BBB-
NPRL4009	0.39454	BBB-
NPRL4010	0.91712	BBB+
NPRL4011	0.00276	BBB-
NPRL4012	0.30164	BBB-
NPRL4013	0.43179	BBB-
NPRL4014	0.61741	BBB+
NPRL4016	0.02210	BBB-
NPRL4017	0.35706	BBB-
NPRL4018	0.01530	BBB-
NPRL4019	0.42890	BBB-
NPRL4020	0.87586	BBB+
NPRL4021	0.98304	BBB+
NPRL4022	0.00445	BBB-
NPRL4023	0.08210	BBB-
NPRL4025	0.86885	BBB+
NPRL4027	0.10585	BBB-
NPRL4028	0.11893	BBB-
NPRL4030	0.01475	BBB-
NPRL4031	0.45484	BBB-
NPRL4032	0.28226	BBB-
NPRL4033	0.44440	BBB-
NPRL4034	0.89844	BBB+
NPRL4035	0.36040	BBB-
NPRL4036	0.35500	BBB-
NPRL4037	0.43553	BBB-
NPRL4038	0.72835	BBB+
NPRL4039	0.87881	BBB+
NPRL4040	0.04739	BBB-
NPRL4041	0.51208	BBB+
NPRL4042	0.66459	BBB+
NPRL4043	0.54514	BBB+
NPRL4044	0.92532	BBB+
NPRL4046	0.94330	BBB+
NPRL4047	0.88222	BBB+

NPRL4048	0.94929	BBB+
NPRL4049	0.16499	BBB-
NPRL4050	0.21729	BBB-
NPRL4051	0.90749	BBB+
NPRL4052	0.45754	BBB-
NPRL4054	0.63236	BBB+
NPRL4055	0.90582	BBB+
NPRL4056	0.92750	BBB+
NPRL4057	0.89091	BBB+
NPRL4058	0.74238	BBB+
NPRL4059	0.85178	BBB+
NPRL4060	0.87906	BBB+
NPRL4061	0.94217	BBB+
NPRL4063	0.67231	BBB+
NPRL4064	0.05636	BBB-
NPRL4065	0.03463	BBB-
NPRL4066	0.90902	BBB+
NPRL4067	0.07484	BBB-
NPRL4068	0.74263	BBB+
NPRL4069	0.74367	BBB+
NPRL4070	0.57625	BBB+
NPRL4071	0.99480	BBB+
NPRL4072	0.02609	BBB-
NPRL4073	0.15521	BBB-
NPRL4074	0.28306	BBB-
NPRL4075	0.20431	BBB-
NPRL4076	0.93035	BBB+
NPRL4077	0.59080	BBB+
NPRL4078	0.93470	BBB+
NPRL4079	0.91944	BBB+
NPRL4080	0.99932	BBB+
NPRL4082	0.74615	BBB+
NPRL4083	0.69119	BBB+
NPRL4085	0.46398	BBB-
NPRL4086	0.57453	BBB+
NPRL4087	0.76959	BBB+
NPRL4088	0.79866	BBB+
NPRL4089	0.76395	BBB+
NPRL4090	0.75966	BBB+
NPRL4091	0.71021	BBB+
NPRL4092	0.75926	BBB+
NPRL4093	0.89565	BBB+
NPRL4094	0.86977	BBB+
NPRL4095	0.99449	BBB+
NPRL4096	0.74828	BBB+
NPRL4097	0.54361	BBB+
NPRL4098	0.98470	BBB+
NPRL4099	0.71006	BBB+
NPRL4100	0.87395	BBB+
NPRL4102	0.49151	BBB-
NPRL4103	0.42943	BBB-
NPRL4104	0.23633	BBB-
NPRL4108	0.98362	BBB+
NPRL4112	0.48382	BBB-
NPRL4113	0.99285	BBB+
NPRL4114	0.97699	BBB+
NPRL4115	0.40159	BBB-

NPRL4117	0.15750	BBB-
NPRL4118	0.29237	BBB-
NPRL4119	0.42090	BBB-
NPRL4120	0.18055	BBB-
NPRL4121	0.13124	BBB-
NPRL4123	0.42732	BBB-
NPRL4124	0.02917	BBB-
NPRL4127	0.37628	BBB-
NPRL4128	0.51558	BBB+
NPRL4129	0.00971	BBB-
NPRL4130	0.03452	BBB-
NPRL4131	0.85931	BBB+
NPRL4132	0.55781	BBB+
NPRL4133	0.06823	BBB-
NPRL4134	0.76983	BBB+
NPRL4135	0.74745	BBB+
NPRL4137	0.13149	BBB-
NPRL4138	0.83815	BBB+
NPRL4139	0.02699	BBB-
NPRL4140	0.80264	BBB+
NPRL4141	0.81697	BBB+
NPRL4142	0.20076	BBB-
NPRL4143	0.06834	BBB-
NPRL4144	0.45582	BBB-
NPRL4145	0.97433	BBB+
NPRL4148	0.98451	BBB+
NPRL4149	0.00147	BBB-
NPRL4150	0.05937	BBB-
NPRL4152	0.00651	BBB-
NPRL4155	0.63485	BBB+
NPRL4156	0.01248	BBB-
NPRL4157	0.82758	BBB+
NPRL4158	0.68861	BBB+
NPRL4159	0.98992	BBB+
NPRL4160	0.99647	BBB+
NPRL4161	0.98784	BBB+
NPRL4162	0.50387	BBB+
NPRL4163	0.04696	BBB-
NPRL4164	0.74730	BBB+
NPRL4165	0.82626	BBB+
NPRL4166	0.86972	BBB+
NPRL4167	0.83080	BBB+
NPRL4169	0.12867	BBB-
NPRL4170	0.85918	BBB+
NPRL4171	0.09026	BBB-
NPRL4172	0.16817	BBB-
NPRL4173	0.08041	BBB-
NPRL4175	0.02075	BBB-
NPRL4176	0.01049	BBB-
NPRL4177	0.40582	BBB-
NPRL4178	0.21777	BBB-
NPRL4180	0.11105	BBB-
NPRL4182	0.76226	BBB+
NPRL4184	0.03047	BBB-
NPRL4185	0.06612	BBB-
NPRL4186	0.18543	BBB-
NPRL4187	0.21906	BBB-

NPRL4188	0.18693	BBB-
NPRL4189	0.46447	BBB-
NPRL4190	0.05935	BBB-
NPRL4191	0.64199	BBB+
NPRL4192	0.06718	BBB-
NPRL4193	0.86680	BBB+
NPRL4194	0.79681	BBB+
NPRL4195	0.05468	BBB-
NPRL4197	0.73116	BBB+
NPRL4198	0.73751	BBB+
NPRL4199	0.62531	BBB+
NPRL4200	0.19811	BBB-
NPRL4201	0.05772	BBB-
NPRL4202	0.68845	BBB+
NPRL4203	0.42191	BBB-
NPRL4204	0.21774	BBB-
NPRL4206	0.26321	BBB-
NPRL4207	0.91549	BBB+
NPRL4208	0.67200	BBB+
NPRL4209	0.13993	BBB-
NPRL4210	0.16284	BBB-
NPRL4211	0.72596	BBB+
NPRL4212	0.56857	BBB+
NPRL4213	0.48042	BBB-
NPRL4214	0.28580	BBB-
NPRL4215	0.74200	BBB+
NPRL4216	0.68991	BBB+
NPRL4217	0.96658	BBB+
NPRL4218	0.79433	BBB+
NPRL4219	0.20081	BBB-
NPRL4220	0.12525	BBB-
NPRL4221	0.86773	BBB+
NPRL4222	0.55308	BBB+
NPRL4223	0.85474	BBB+
NPRL4224	0.64000	BBB+
NPRL4225	0.56177	BBB+
NPRL4226	0.78983	BBB+
NPRL4227	0.80630	BBB+
NPRL4228	0.70945	BBB+
NPRL4229	0.85191	BBB+
NPRL4230	0.15658	BBB-
NPRL4231	0.45327	BBB-
NPRL4232	0.18226	BBB-
NPRL4233	0.41479	BBB-
NPRL4234	0.82113	BBB+
NPRL4235	0.89294	BBB+
NPRL4237	0.92713	BBB+
NPRL4238	0.03770	BBB-
NPRL4239	0.17309	BBB-
NPRL4240	0.29258	BBB-
NPRL4241	0.20594	BBB-
NPRL4242	0.55192	BBB+
NPRL4243	0.19513	BBB-
NPRL4252	0.32862	BBB-
NPRL4255	0.99987	BBB+
NPRL4256	0.99764	BBB+
NPRL4257	0.99782	BBB+

NPRL4260	0.00151	BBB-
NPRL4261	0.51842	BBB+
NPRL4263	0.00346	BBB-
NPRL4264	0.45759	BBB-
NPRL4265	0.00284	BBB-
NPRL4266	0.69047	BBB+
NPRL4267	0.00011	BBB-
NPRL4271	0.99629	BBB+
NPRL4274	0.91644	BBB+
NPRL4275	0.24469	BBB-
NPRL4277	0.55232	BBB+
NPRL4278	0.74207	BBB+
NPRL4279	0.78599	BBB+
NPRL4280	0.96334	BBB+
NPRL4281	0.87871	BBB+
NPRL4283	0.11788	BBB-
NPRL4284	0.65419	BBB+
NPRL4285	0.98976	BBB+
NPRL4286	0.99478	BBB+
NPRL4287	0.54969	BBB+
NPRL4288	0.93217	BBB+
NPRL4289	0.36536	BBB-
NPRL4290	0.95864	BBB+
NPRL4292	0.50357	BBB+
NPRL4293	0.89897	BBB+
NPRL4294	0.33783	BBB-
NPRL4295	0.99451	BBB+
NPRL4296	0.99011	BBB+
NPRL4298	0.88663	BBB+
NPRL4299	0.84822	BBB+
NPRL4300	0.96130	BBB+
NPRL4301	0.54873	BBB+
NPRL4302	0.53852	BBB+
NPRL4303	0.71813	BBB+
NPRL4304	0.52768	BBB+
NPRL4305	0.99632	BBB+
NPRL4306	0.91362	BBB+
NPRL4307	0.96152	BBB+
NPRL4309	0.99199	BBB+
NPRL4310	0.81640	BBB+
NPRL4311	0.97844	BBB+
NPRL4312	0.99614	BBB+
NPRL4313	0.10736	BBB-
NPRL4315	0.99543	BBB+
NPRL4316	0.92516	BBB+
NPRL4317	0.95726	BBB+
NPRL4318	0.96879	BBB+
NPRL4319	0.99883	BBB+
NPRL4320	0.96699	BBB+
NPRL4321	0.99591	BBB+
NPRL4322	0.29964	BBB-
NPRL4324	0.97106	BBB+
NPRL4328	0.42825	BBB-
NPRL4329	0.95395	BBB+
NPRL4330	0.84242	BBB+
NPRL4331	0.04730	BBB-
NPRL4334	0.97911	BBB+

NPRL4335	0.99592	BBB+
NPRL4337	0.84290	BBB+
NPRL4338	0.45631	BBB-
NPRL4339	0.33738	BBB-
NPRL4340	0.49852	BBB-
NPRL4341	0.72233	BBB+
NPRL4342	0.26032	BBB-
NPRL4343	0.90245	BBB+
NPRL4345	0.07988	BBB-
NPRL4346	0.20149	BBB-
NPRL4347	0.32890	BBB-
NPRL4348	0.43199	BBB-
NPRL4349	0.03449	BBB-
NPRL4350	0.74095	BBB+
NPRL4351	0.19552	BBB-
NPRL4352	0.76825	BBB+
NPRL4353	0.00935	BBB-
NPRL4354	0.88307	BBB+
NPRL4355	0.74887	BBB+
NPRL4356	0.41112	BBB-
NPRL4357	0.31812	BBB-
NPRL4358	0.33973	BBB-
NPRL4359	0.43589	BBB-
NPRL4360	0.50321	BBB+
NPRL4361	0.86484	BBB+
NPRL4362	0.90445	BBB+
NPRL4363	0.90925	BBB+
NPRL4364	0.75470	BBB+
NPRL4365	0.98047	BBB+
NPRL4366	0.72109	BBB+
NPRL4367	0.47960	BBB-
NPRL4369	0.58096	BBB+
NPRL4370	0.80176	BBB+
NPRL4372	0.22599	BBB-
NPRL4373	0.97593	BBB+
NPRL4374	0.35927	BBB-
NPRL4375	0.80090	BBB+
NPRL4376	0.62165	BBB+
NPRL4377	0.09094	BBB-
NPRL4378	0.87013	BBB+
NPRL4379	0.06726	BBB-
NPRL4380	0.79786	BBB+
NPRL4381	0.73377	BBB+
NPRL4382	0.72277	BBB+
NPRL4383	0.89806	BBB+
NPRL4384	0.66955	BBB+
NPRL4385	0.98083	BBB+
NPRL4386	0.00270	BBB-
NPRL4387	0.21950	BBB-
NPRL4388	0.08749	BBB-
NPRL4389	0.01653	BBB-
NPRL4391	0.90127	BBB+
NPRL4392	0.00058	BBB-
NPRL4393	0.89076	BBB+
NPRL4394	0.00078	BBB-
NPRL4395	0.00041	BBB-
NPRL4396	0.00377	BBB-

NPRL4397	0.55573	BBB+
NPRL4398	0.00150	BBB-
NPRL4399	0.28745	BBB-
NPRL4400	0.51389	BBB+
NPRL4401	0.54242	BBB+
NPRL4402	0.43549	BBB-
NPRL4403	0.86849	BBB+
NPRL4404	0.57885	BBB+
NPRL4405	0.93584	BBB+
NPRL4406	0.94760	BBB+
NPRL4407	0.95577	BBB+
NPRL4408	0.93649	BBB+
NPRL4409	0.98586	BBB+
NPRL4410	0.93789	BBB+
NPRL4411	0.97338	BBB+
NPRL4413	0.97832	BBB+
NPRL4414	0.40227	BBB-
NPRL4415	0.90462	BBB+
NPRL4416	0.89769	BBB+
NPRL4417	0.92398	BBB+
NPRL4418	0.95361	BBB+
NPRL4419	0.90049	BBB+
NPRL4420	0.95185	BBB+
NPRL4421	0.89099	BBB+
NPRL4422	0.82808	BBB+
NPRL4423	0.67422	BBB+
NPRL4424	0.92782	BBB+
NPRL4425	0.55048	BBB+
NPRL4426	0.58501	BBB+
NPRL4427	0.41571	BBB-
NPRL4428	0.43920	BBB-
NPRL4429	0.86836	BBB+
NPRL4430	0.42967	BBB-
NPRL4431	0.00095	BBB-
NPRL4432	0.00564	BBB-
NPRL4433	0.99138	BBB+
NPRL4434	0.00389	BBB-
NPRL4435	0.98672	BBB+
NPRL4436	0.00094	BBB-
NPRL4437	0.95837	BBB+
NPRL4438	0.99112	BBB+
NPRL4439	0.95789	BBB+
NPRL4440	0.99568	BBB+
NPRL4441	0.94700	BBB+
NPRL4443	0.20537	BBB-
NPRL4444	0.79548	BBB+
NPRL4446	0.88321	BBB+
NPRL4447	0.44232	BBB-
NPRL4448	0.73846	BBB+
NPRL4449	0.42619	BBB-
NPRL4450	0.89615	BBB+
NPRL4451	0.94214	BBB+
NPRL4452	0.59729	BBB+
NPRL4453	0.52187	BBB+
NPRL4454	0.11551	BBB-
NPRL4455	0.81085	BBB+
NPRL4456	0.59479	BBB+

NPRL4457	0.98887	BBB+
NPRL4458	0.30352	BBB-
NPRL4459	0.94064	BBB+
NPRL4460	0.69152	BBB+
NPRL4461	0.13839	BBB-
NPRL4463	0.02179	BBB-
NPRL4464	0.66462	BBB+
NPRL4465	0.90779	BBB+
NPRL4466	0.98824	BBB+
NPRL4468	0.51920	BBB+
NPRL4469	0.90396	BBB+
NPRL4471	0.79551	BBB+
NPRL4472	0.98691	BBB+
NPRL4474	0.90147	BBB+
NPRL4475	0.99299	BBB+
NPRL4476	0.89225	BBB+
NPRL4477	0.93668	BBB+
NPRL4480	0.99093	BBB+
NPRL4483	0.27171	BBB-
NPRL4484	0.04920	BBB-
NPRL4485	0.57253	BBB+
NPRL4486	0.88013	BBB+
NPRL4487	0.42071	BBB-
NPRL4488	0.08402	BBB-
NPRL4489	0.27565	BBB-
NPRL4490	0.47974	BBB-
NPRL4491	0.13495	BBB-
NPRL4492	0.03369	BBB-
NPRL4493	0.70009	BBB+
NPRL4494	0.95178	BBB+
NPRL4495	0.67945	BBB+
NPRL4496	0.98245	BBB+
NPRL4497	0.94992	BBB+
NPRL4499	0.09385	BBB-
NPRL4500	0.21140	BBB-
NPRL4501	0.94399	BBB+
NPRL4502	0.45936	BBB-
NPRL4503	0.96573	BBB+
NPRL4504	0.64268	BBB+
NPRL4505	0.39445	BBB-
NPRL4506	0.00372	BBB-
NPRL4507	0.08888	BBB-
NPRL4508	0.41376	BBB-
NPRL4510	0.00562	BBB-
NPRL4513	0.88611	BBB+
NPRL4515	0.33408	BBB-
NPRL4518	0.35489	BBB-
NPRL4519	0.20094	BBB-
NPRL4520	0.02036	BBB-
NPRL4521	0.00008	BBB-
NPRL4522	0.09637	BBB-
NPRL4524	0.00084	BBB-
NPRL4526	0.22548	BBB-
NPRL4527	0.09796	BBB-
NPRL4528	0.35555	BBB-
NPRL4529	0.49109	BBB-
NPRL4530	0.00208	BBB-

NPRL4531	0.96089	BBB+
NPRL4532	0.17685	BBB-
NPRL4533	0.68535	BBB+
NPRL4534	0.00794	BBB-
NPRL4535	0.95385	BBB+
NPRL4536	0.24624	BBB-
NPRL4537	0.99426	BBB+
NPRL4538	0.71705	BBB+
NPRL4539	0.79118	BBB+
NPRL4540	0.94677	BBB+
NPRL4541	0.76061	BBB+
NPRL4542	0.98959	BBB+
NPRL4543	0.93072	BBB+
NPRL4544	0.91735	BBB+
NPRL4545	0.60930	BBB+
NPRL4546	0.00490	BBB-
NPRL4547	0.79226	BBB+
NPRL4548	0.12983	BBB-
NPRL4549	0.56029	BBB+
NPRL4550	0.47152	BBB-
NPRL4551	0.34051	BBB-
NPRL4552	0.18262	BBB-
NPRL4553	0.48072	BBB-
NPRL4554	0.23507	BBB-
NPRL4555	0.10443	BBB-
NPRL4556	0.62502	BBB+
NPRL4557	0.82824	BBB+
NPRL4558	0.80099	BBB+
NPRL4559	0.60758	BBB+
NPRL4560	0.46376	BBB-
NPRL4561	0.96999	BBB+
NPRL4562	0.94883	BBB+
NPRL4564	0.69551	BBB+
NPRL4565	0.04749	BBB-
NPRL4566	0.05673	BBB-
NPRL4567	0.53267	BBB+
NPRL4568	0.93883	BBB+
NPRL4569	0.18627	BBB-
NPRL4572	0.64933	BBB+
NPRL4573	0.01646	BBB-
NPRL4574	0.00656	BBB-
NPRL4575	0.85111	BBB+
NPRL4577	0.12251	BBB-
NPRL4578	0.99952	BBB+
NPRL4581	0.99793	BBB+
NPRL4583	0.00335	BBB-
NPRL4584	0.99458	BBB+
NPRL4589	0.00210	BBB-
NPRL4590	0.01011	BBB-
NPRL4591	0.00146	BBB-
NPRL4592	0.00246	BBB-
NPRL4593	0.59141	BBB+
NPRL4594	0.62706	BBB+
NPRL4595	0.03096	BBB-
NPRL4596	0.00593	BBB-
NPRL4597	0.01805	BBB-
NPRL4598	0.00329	BBB-

NPRL4599	0.00127	BBB-
NPRL4600	0.00028	BBB-
NPRL4601	0.01625	BBB-
NPRL4602	0.00252	BBB-
NPRL4603	0.00189	BBB-
NPRL4604	0.00113	BBB-
NPRL4605	0.00204	BBB-
NPRL4607	0.85933	BBB+
NPRL4608	0.00170	BBB-
NPRL4609	0.00120	BBB-
NPRL4610	0.00437	BBB-
NPRL4611	0.00037	BBB-
NPRL4612	0.00045	BBB-
NPRL4613	0.00069	BBB-
NPRL4614	0.00211	BBB-
NPRL4615	0.00019	BBB-
NPRL4616	0.00328	BBB-
NPRL4617	0.00138	BBB-
NPRL4618	0.00076	BBB-
NPRL4619	0.95709	BBB+
NPRL4620	0.98733	BBB+
NPRL4621	0.98052	BBB+
NPRL4622	0.82629	BBB+
NPRL4623	0.00036	BBB-
NPRL4624	0.00629	BBB-
NPRL4625	0.84189	BBB+
NPRL4626	0.09256	BBB-
NPRL4628	0.71449	BBB+
NPRL4629	0.98404	BBB+
NPRL4630	0.25952	BBB-
NPRL4631	0.96790	BBB+
NPRL4632	0.84800	BBB+
NPRL4633	0.90594	BBB+
NPRL4634	0.36585	BBB-
NPRL4635	0.00455	BBB-
NPRL4636	0.92312	BBB+
NPRL4637	0.02636	BBB-
NPRL4638	0.13832	BBB-
NPRL4639	0.01216	BBB-
NPRL6590	0.35690	BBB-
NPRL6591	0.23111	BBB-
NPRL6592	0.06097	BBB-
NPRL6593	0.08896	BBB-
NPRL6594	0.26927	BBB-
NPRL6597	0.01995	BBB-
NPRL6599	0.69608	BBB+
NPRL6600	0.00382	BBB-
NPRL6601	0.00058	BBB-
NPRL6602	0.00541	BBB-
NPRL6603	0.13609	BBB-
NPRL6605	0.02791	BBB-
NPRL6606	0.96722	BBB+
NPRL6607	0.68004	BBB+
NPRL6608	0.15102	BBB-
NPRL6609	0.72630	BBB+
NPRL6610	0.05184	BBB-
NPRL6611	0.98397	BBB+

NPRL6613	0.94790	BBB+
NPRL6614	0.97769	BBB+
NPRL6615	0.98631	BBB+
NPRL6616	0.97228	BBB+
NPRL6617	0.99527	BBB+
NPRL6618	0.98694	BBB+
NPRL6620	0.93678	BBB+
NPRL6622	0.99340	BBB+
NPRL6623	0.00349	BBB-
NPRL6624	0.27429	BBB-
NPRL6625	0.96988	BBB+
NPRL6626	0.87049	BBB+
NPRL6628	0.00859	BBB-
NPRL6629	0.48061	BBB-
NPRL6630	0.08425	BBB-
NPRL6632	0.31201	BBB-
NPRL6633	0.77122	BBB+
NPRL6635	0.71418	BBB+
NPRL6637	0.72268	BBB+
NPRL6638	0.98055	BBB+
NPRL6639	0.99979	BBB+
NPRL6640	0.99778	BBB+
NPRL6641	0.72551	BBB+
NPRL6644	0.90114	BBB+
NPRL6645	0.60682	BBB+
NPRL6646	0.05813	BBB-
NPRL6647	0.15101	BBB-
NPRL6648	0.95063	BBB+
NPRL6649	0.59658	BBB+
NPRL6650	0.17506	BBB-
NPRL6651	0.54777	BBB+
NPRL6652	0.77126	BBB+
NPRL6653	0.79700	BBB+
NPRL6654	0.93842	BBB+
NPRL6655	0.96696	BBB+
NPRL6656	0.95426	BBB+
NPRL6657	0.18602	BBB-
NPRL6658	0.99687	BBB+
NPRL6659	0.95396	BBB+
NPRL6660	0.21014	BBB-
NPRL6661	0.00545	BBB-
NPRL6662	0.93108	BBB+
NPRL6663	0.04357	BBB-
NPRL6664	0.22898	BBB-
NPRL6665	0.87352	BBB+
NPRL6666	0.58048	BBB+
NPRL6667	0.03369	BBB-
NPRL6668	0.00304	BBB-
NPRL6669	0.07152	BBB-
NPRL6670	0.99749	BBB+
NPRL6671	0.97529	BBB+
NPRL6672	0.98699	BBB+
NPRL6673	0.14597	BBB-
NPRL6674	0.84582	BBB+
NPRL6675	0.98180	BBB+
NPRL6676	0.90579	BBB+
NPRL6677	0.88371	BBB+

NPRL6678	0.99962	BBB+
NPRL6679	0.99439	BBB+
NPRL6680	0.98487	BBB+
NPRL6681	0.76974	BBB+
NPRL6682	0.69831	BBB+
NPRL6683	0.11165	BBB-
NPRL6684	0.85156	BBB+
NPRL6686	0.98363	BBB+
NPRL6688	0.99757	BBB+
NPRL6689	0.93319	BBB+
NPRL6692	0.97802	BBB+
NPRL6694	0.99802	BBB+
NPRL6699	0.54080	BBB+
NPRL6701	0.95323	BBB+
NPRL6703	0.38108	BBB-
NPRL6704	0.98975	BBB+
NPRL6709	0.61126	BBB+
NPRL6710	0.99358	BBB+
NPRL6711	0.95776	BBB+
NPRL6712	0.97830	BBB+
NPRL6713	0.04361	BBB-
NPRL6714	0.93190	BBB+
NPRL6715	0.97718	BBB+
NPRL6719	0.49683	BBB-
NPRL6720	0.32331	BBB-
NPRL6724	0.99491	BBB+
NPRL6728	0.93104	BBB+
NPRL6732	0.47680	BBB-
NPRL6733	0.45399	BBB-
NPRL6734	0.99454	BBB+
NPRL6737	0.62892	BBB+
NPRL6738	0.00116	BBB-
NPRL6739	0.43116	BBB-
NPRL6741	0.59115	BBB+
NPRL6742	0.93041	BBB+
NPRL6743	0.92543	BBB+
NPRL6744	0.99052	BBB+
NPRL6745	0.00414	BBB-
NPRL6748	0.95606	BBB+
NPRL6749	0.79351	BBB+
NPRL6750	0.92481	BBB+
NPRL6751	0.99332	BBB+
NPRL6752	0.94238	BBB+
NPRL6753	0.79734	BBB+
NPRL6759	0.63661	BBB+
NPRL6760	0.99902	BBB+
NPRL6761	0.46869	BBB-
NPRL6763	0.95216	BBB+
NPRL6769	0.88020	BBB+
NPRL6770	0.55887	BBB+
NPRL6771	0.89609	BBB+
NPRL6774	0.89863	BBB+
NPRL6775	0.97273	BBB+
NPRL6776	0.77564	BBB+
NPRL6777	0.99933	BBB+
NPRL6778	0.99035	BBB+
NPRL6779	0.68859	BBB+

NPRL6780	0.99955	BBB+
NPRL6782	0.97103	BBB+
NPRL880	0.93135	BBB+
NPRL881	0.99380	BBB+
NPRL882	0.84905	BBB+
NPRL883	0.94611	BBB+
NPRL884	0.33670	BBB-
NPRL885	0.38359	BBB-
NPRL886	0.91031	BBB+
NPRL887	0.30461	BBB-
NPRL891	0.52341	BBB+
NPRL892	0.48024	BBB-
NPRL894	0.98749	BBB+
NPRL895	0.20016	BBB-
NPRL898	0.99230	BBB+
NPRL899	0.79746	BBB+
NPRL901	0.20136	BBB-
NPRL902	0.02254	BBB-
NPRL903	0.01456	BBB-
NPRL904	0.02048	BBB-
NPRL906	0.00153	BBB-
NPRL908	0.28221	BBB-
NPRL910	0.25753	BBB-
NPRL912	0.17984	BBB-
NPRL913	0.49927	BBB-
NPRL914	0.02011	BBB-
NPRL916	0.31739	BBB-
NPRL920	0.93298	BBB+
NPRL921	0.80231	BBB+
NPRL922	0.12157	BBB-
NPRL923	0.95214	BBB+
NPRL924	0.95577	BBB+
NPRL925	0.95649	BBB+
NPRL927	0.97281	BBB+
NPRL929	0.55780	BBB+
NPRL930	0.85148	BBB+
NPRL931	0.82672	BBB+
NPRL933	0.07027	BBB-
NPRL934	0.37147	BBB-
NPRL935	0.06169	BBB-
NPRL936	0.05551	BBB-
NPRL941	0.73517	BBB+
NPRL942	0.01781	BBB-
NPRL943	0.73453	BBB+
NPRL944	0.00057	BBB-
NPRL945	0.01220	BBB-
NPRL946	0.08504	BBB-
NPRL947	0.00085	BBB-
NPRL948	0.50521	BBB+
NPRL950	0.22470	BBB-
NPRL951	0.03467	BBB-
NPRL952	0.95860	BBB+
NPRL953	0.17211	BBB-
NPRL955	0.00607	BBB-
NPRL956	0.01266	BBB-
NPRL957	0.01594	BBB-
NPRL958	0.75195	BBB+

NPRL959	0.91485	BBB+
NPRL960	0.48397	BBB-
NPRL961	0.47950	BBB-
NPRL967	0.65590	BBB+
NPRL968	0.54103	BBB+
NPRL969	0.63684	BBB+
NPRL970	0.44128	BBB-
NPRL971	0.97612	BBB+
NPRL972	0.93999	BBB+
NPRL973	0.98837	BBB+
NPRL974	0.69664	BBB+
NPRL977	0.39889	BBB-
NPRL978	0.02367	BBB-
NPRL979	0.65860	BBB+
NPRL981	0.94922	BBB+
NPRL982	0.98652	BBB+
NPRL983	0.63734	BBB+
NPRL984	0.90813	BBB+
NPRL985	0.53502	BBB+
NPRL986	0.47448	BBB-
NPRL987	0.20573	BBB-
NPRL988	0.56352	BBB+
NPRL989	0.25759	BBB-
NPRL990	0.37355	BBB-
NPRL991	0.87675	BBB+
NPRL992	0.11134	BBB-
NPRL994	0.89732	BBB+
NPRL995	0.96213	BBB+
NPRL996	0.99383	BBB+
NPRL997	0.00398	BBB-
